# Lack of Neuronal IFN-β-IFNAR Causes Lewy Body- and Parkinson’s Disease-like Dementia

**DOI:** 10.1016/j.cell.2015.08.069

**Published:** 2015-10-08

**Authors:** Patrick Ejlerskov, Jeanette Göransdotter Hultberg, JunYang Wang, Robert Carlsson, Malene Ambjørn, Martin Kuss, Yawei Liu, Giovanna Porcu, Kateryna Kolkova, Carsten Friis Rundsten, Karsten Ruscher, Bente Pakkenberg, Tobias Goldmann, Desiree Loreth, Marco Prinz, David C. Rubinsztein, Shohreh Issazadeh-Navikas

**Affiliations:** 1 Biotech Research and Innovation Centre, University of Copenhagen, 2200 Copenhagen, Denmark; 2 Department of Clinical Sciences, Lund University, 22100 Lund, Sweden; 3 Research Laboratory for Stereology and Neuroscience, Bispebjerg University Hospital, 2200 Copenhagen, Denmark; 4 Institute for Neuropathology, University of Freiburg, 79106 Freiburg, Germany; 5 Department of Neurology, University of Freiburg, 79106 Freiburg, Germany; 6 Centre for Biological Signaling Studies, University of Freiburg, 79106 Freiburg, Germany; 7 Department of Medical Genetics, Cambridge Institute for Medical Research, Cambridge CB2 0XY, UK

## Abstract

Neurodegenerative diseases have been linked to inflammation, but whether altered immunomodulation plays a causative role in neurodegeneration is not clear. We show that lack of cytokine interferon-β (IFN-β) signaling causes spontaneous neurodegeneration in the absence of neurodegenerative disease-causing mutant proteins. Mice lacking *Ifnb* function exhibited motor and cognitive learning impairments with accompanying α-synuclein-containing Lewy bodies in the brain, as well as a reduction in dopaminergic neurons and defective dopamine signaling in the nigrostriatal region. Lack of IFN-β signaling caused defects in neuronal autophagy prior to α-synucleinopathy, which was associated with accumulation of senescent mitochondria. Recombinant IFN-β promoted neurite growth and branching, autophagy flux, and α-synuclein degradation in neurons. In addition, lentiviral IFN-β overexpression prevented dopaminergic neuron loss in a familial Parkinson’s disease model. These results indicate a protective role for IFN-β in neuronal homeostasis and validate *Ifnb* mutant mice as a model for sporadic Lewy body and Parkinson’s disease dementia.

## Highlights

• Lack of neuronal IFN-β-IFNAR signaling causes brain Lewy body accumulation
• IFN-β deficiency causes late-stage autophagy block and thereby α-synuclein aggregation
• IFN-β promotes neuronal autophagy and α-synuclein clearance
• *Ifnb* gene therapy prevents dopaminergic neuron loss in a familial PD model

## Graphical Abstract

**Figure:**
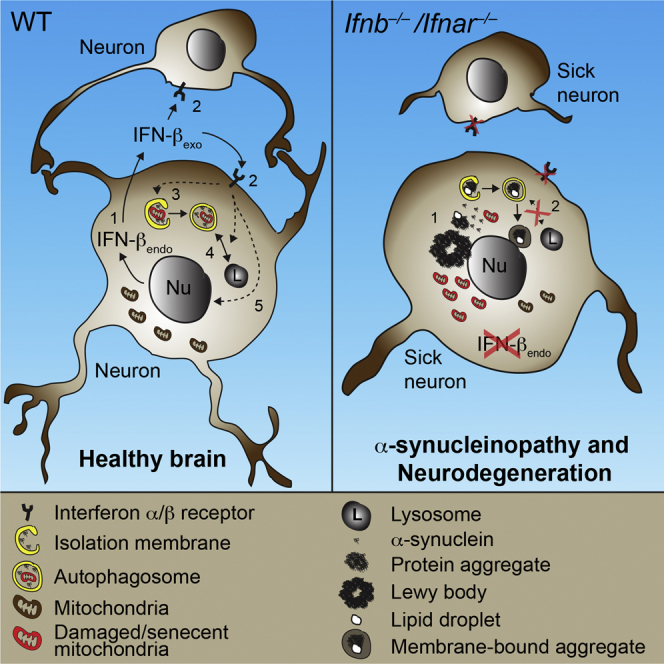

## Teaser

Lack of the immunomodulatory cytokine interferon-β (IFN-β) causes spontaneous neurodegeneration resembling sporadic Lewy body and Parkinson’s disease dementia due to defects in neuronal autophagy.

## Introduction

Neurodegenerative diseases have disrupted neuronal homeostasis and their pathologies often overlap. Protein aggregates containing α-synuclein (α-syn), which eventually forms larger Lewy bodies (LBs), are seen in Parkinson’s disease (PD), dementia with Lewy bodies (DLB), multiple system atrophy, and in some forms of Alzheimer’s disease (AD), all neurodegenerative diseases associated with aging (Arima et al., 1999; Francis, 2009; Lippa et al., 1998). Often, the protein aggregates contain hyperphosphorylated tau and ubiquitin (Jellinger and Attems, 2008).

Neurodegenerative events in these diseases are linked to inflammation (Mrak and Griffin, 2007; Tansey et al., 2008), but, despite this link, defects in genes regulating inflammation do not have an established causative role in neurodegeneration. We report that deletion of *Ifnb*, which encodes interferon-β (IFN-β), is sufficient to cause a cascade of neurodegenerative events. IFN-β belongs to the type I interferon family of cytokines and binding to its receptor, interferon-α/β receptor (IFNAR), results in immunoregulation including anti-viral and anti-inflammatory effects (Prinz et al., 2008; Teige et al., 2003, 2006) and benefits for multiple sclerosis patients (Liu et al., 2014; Yong et al., 1998). However, the role of IFN-β in classic neurodegenerative diseases is unknown. We report that *Ifnb*^*–/–*^ mice developed spontaneous pathologies mimicking major aspects of human neurodegeneration such as PD and DLB. *Ifnb*^*–/–*^ mice had age-associated motor learning defects, neuromuscular deficiencies, and cognitive impairment. *Ifnb*^*–/–*^ pathology was associated with LBs resulting from defective neuronal autophagy. Autophagy, a pathway that degrades long-lived proteins, organelles, lipids, and protein aggregates, is essential for neuronal homeostasis (Harris and Rubinsztein, 2012), and deleting neural autophagy-regulating genes leads to neurodegeneration (Hara et al., 2006; Komatsu et al., 2006).

Our findings indicate a central role for IFN-β in neuronal homeostasis as a regulator of autophagy-mediated protein degradation and accentuates *Ifnb*^*–/–*^ mice as a model for neurodegenerative diseases with α-synucleinopathy and dementia such as PD and DLB.

## Results

### *Ifnb*^*–/–*^ Mice Exhibit Behavioral and Cognitive Impairments and Neurodegeneration

**Figure 1:**
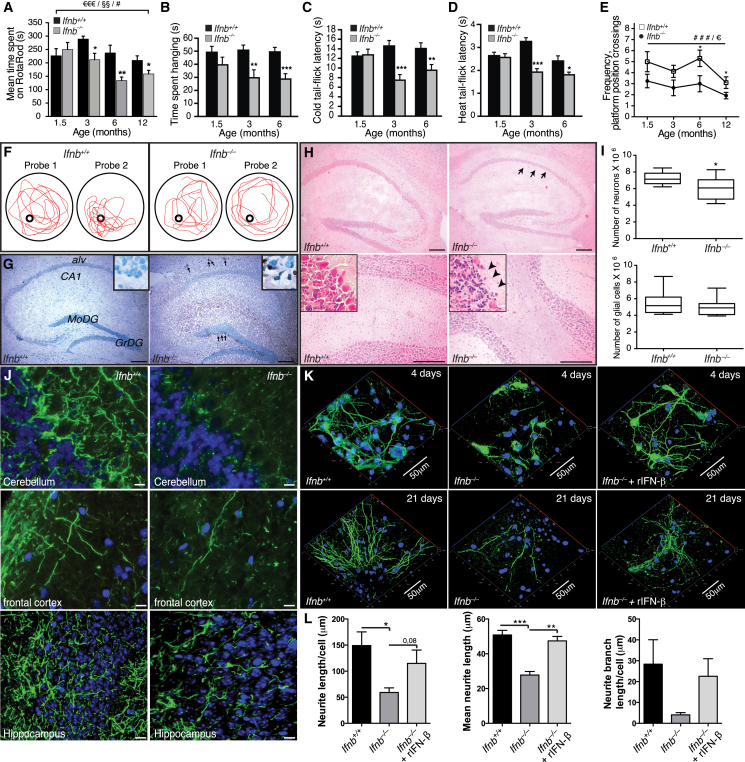
*Ifnb*^*–/–*^ Mice Exhibit Spontaneous Behavioral and Cognitive Impairments and Neurodegeneration (A) Motor coordination by RotaRod. Data are mean second(s) ± SEM, n = 8–13/group. ^∗^p < 0.05, ^∗∗^p < 0.01 for genotype effect per time point by unpaired Student’s t test; ^#^p < 0.05 for aging effect. ^€€€^p < 0.001 and ^§§^p < 0.01 for genotype and aging effect, respectively, over time, by two-way ANOVA. (B) Wire suspension performance. Data are mean ± SEM, 9–13/group. (C and D) (C) Cold and (D) heat tail-flick latency. Data are mean ± SEM, 9–11/group. (B–D) Unpaired Student’s t test. ^∗^p < 0.05, ^∗∗^p < 0.01, ^∗∗∗^p < 0.001. (E) Morris water maze test. Data are mean ± SEM, 8–21 mice/group. ^###^p < 0.001 shows genotype effect; ^€^p < 0.05 shows age effect; ^∗^p < 0.05 comparing genotypes by two-way ANOVA and (^∗^) with Bonferroni post hoc test. (F) Representative maze swimming pattern on probes 1 and 2. (G) TUNEL staining of hippocampus from 1.5-month-old mice with methyl green nuclear counterstaining. MoDG, molecular dentate gyrus; GrDG, granular dentate gyrus; alv, alveus. Scale bar, 200 μm. (H) H&E brain staining showing loss of granular cell layer and Purkinje cells (arrows) in *Ifnb*^*–/–*^ hippocampus (upper) and cerebellum (lower). Scale bar, 200 μm. (I) Number of neurons and glial cells in hippocampus of 3- to 6-month-old mice. Data are mean ± SEM, n = 6 mice/group. ^∗^p < 0.05 using Student’s t test. (J) IF of 3-month-old brain sections. Scale bars, 10 μm. (K) Z stack projections of CNs cultured for 4 and 21 days with or without rIFN-β. Scale bar, 50 μm. (L) Quantified neurite length from 4-day-old CNs. Data are mean ± SEM, n = 3–4, counting 11–32 cells per experiment. ^∗^p < 0.05, ^∗∗^p < 0.01, ^∗∗∗^p < 0.001 by one-way ANOVA. See also Figure S1 and Table S1.

**Figure S1:**
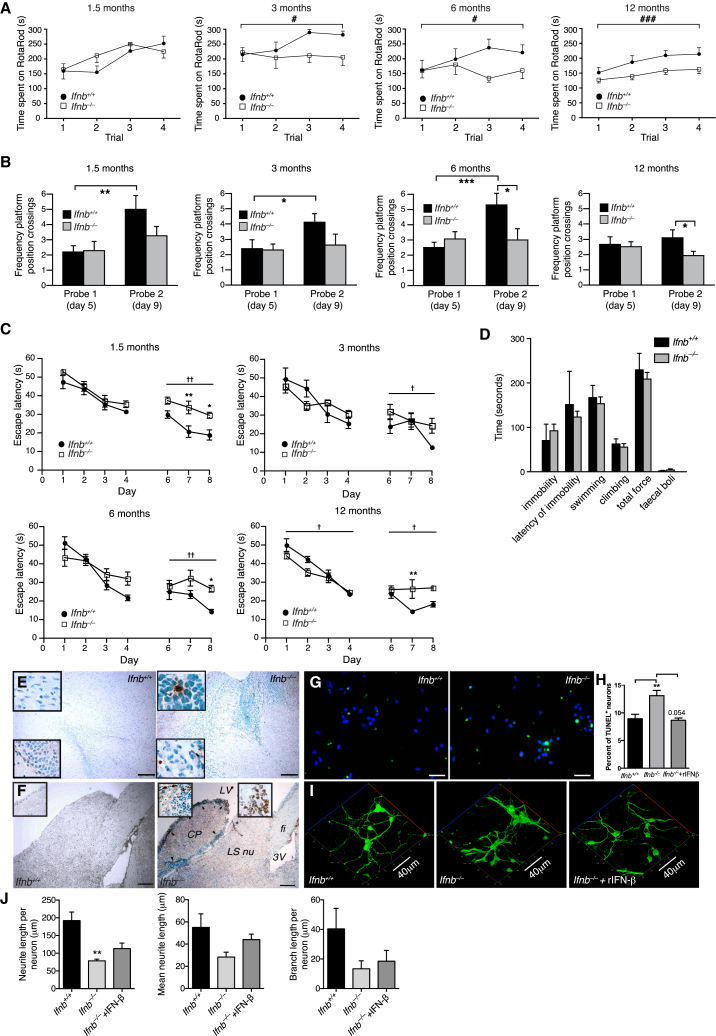
*Ifnb*^*–/–*^ Mice Show Behavioral Deficits, Neuronal Apoptosis, and Neurite Retraction, Related to Figure 1 (A) Motor-coordination and -learning as assessed by seconds (s) spent on RotaRod for each trial. Data are mean ± SEM, 8–13 mice per group. Two-way ANOVA; ^#^p < 0.05, and ^###^p < 0.001 were conducted to assess the effects of genotype and trials. (B and C) Performance of *Ifnb*^*+/+*^ and *Ifnb*^*–/–*^ mice at different ages in Morris Water Maze on probe day 1 and 2. (B) Graphs show mean frequency of platform position crossings. Data are mean ± SEM of 8–21 mice per group. Student’s t test was used to assess differences in frequency of platform position crossings between probe trials within each genotype and age group, and between genotypes of the same age within a probe trial. ^∗^p < 0.05; ^∗∗^p < 0.01; ^∗∗∗^p < 0.001. (C) Escape latency in seconds (s). ^†^p < 0.05 and ^††^p < 0.01 by two-way repeated-measures ANOVA comparing learning curves by genotype; ^∗^p < 0.05 by Bonferroni post hoc test shows significant genotype effect within each learning day (trials). Data are mean ± SEM of 8–21 mice per group. (D) Forced swimming test of *Ifnb*^*+/+*^ and *Ifnb*^*–/–*^ mice. Data represent mean ± SEM. Student’s t test showed no significant differences between the genotypes. (E and F) TUNEL staining of brain with methyl green counterstaining for nuclei showing (E) 1.5-month-old olfactory bulb areas and (F) 12-month-old *caudate putamen*. TUNEL-positive staining (arrows) are present in *Ifnb*^*–/–*^ but not in *Ifnb*^*+/+*^. CP: caudate putamen; LS nu: lateral septal nucleus; LV: left ventricle; 3V:3^rd^ ventricle; fi: fimbria. Scale bar, 200 μm. (G) IF images showing TUNEL staining of cerebellar granular neurons (CGNs) from *Ifnb*^*+/+*^ and *Ifnb*^*–/–*^ mice. Scale bar, 50 μm. (H) Percentage of TUNEL-positive neurons (in vitro cultured CGNs) representing mean ± SEM from n = 3; 6-10 images were quantified from each experiment. ^∗∗^p < 0.01 by unpaired Student’s t test. (I) 3D projected confocal images of CGNs with or without 24 hr of recombinant(r)IFN-β treatment (100 U/ml) showing NF200 (green). Scale bar, 40 μm. (J) Quantification of neurite length per neuron, mean neurite length, and mean branching length of cultured CGN from *Ifnb*^*+/+*^, *Ifnb*^*–/–*^, and *Ifnb*^*–/–*^ treated with rIFN-β (30U/ml) for the whole culture period. Graphs represents mean ± SEM of one representative experiment where 4-5 images containing 3-21 individual neurons were quantified. ^∗^p < 0.05 by Student’s t test.

We examined the effect of *Ifnb* gene deletion on motor coordination, learning, and grip strength. *Ifnb*^*–/–*^ mice were significantly impaired in motor coordination and learning from 3 months compared to age-, sex-, and weight-matched wild-type (WT) *Ifnb*^*+/+*^ littermates and in latency-to-fall time in a wire-suspension test (Figures 1A and 1B). We saw no differences in 1.5-month-old mice, suggesting that impaired motor coordination, balance, and grip strength were associated with age. During repeated motor-learning trials, retention time improved significantly in older *Ifnb*^*+/+*^ mice compared to *Ifnb*^*–/–*^ mice from age 3 months (Figure S1A), showing that reduced motor-learning in *Ifnb*^*–/–*^ mice was associated with aging.

We assessed somatosensory function with nociception cold- and heat-induced tail-flick tests. Latency to tail flick was significantly shorter in *Ifnb*^*–/–*^ than *Ifnb*^*+/+*^ mice (Figures 1C and 1D), indicating hyperalgesia and defective nociception toward temperature-induced pain.

Forced swimming tests found no differences between *Ifnb*^*–/–*^ and *Ifnb*^*+/+*^ mice in swimming pattern, climbing effort, or immobility (Figure S1), so *Ifnb*^*–/–*^ mice were not defective in locomotor activity in water in contrast to land. In water maze tests, *Ifnb*^*–/–*^ mice had significant spatial- and memory-learning deficits that increased with age. During second tests, 6- and 12-month-old, *Ifnb*^*–/–*^ mice had significantly fewer platform position crossings compared to *Ifnb*^*+/+*^ mice, indicating impaired reference memory (Figures 1E and 1F). *Ifnb*^*+/+*^ mice improved between the first and second tests at all ages except 12 months; *Ifnb*^*–/–*^ mice did not improve, indicating impaired spatial learning. This was seen at all ages in *Ifnb*^*–/–*^ mice measured by escape latency time in the second learning block (Figures S1B and S1C). Thus, although *Ifnb*^*–/–*^ mice began with no major behavioral and cognitive defects, they developed age-dependent deficits with increased penetrance (Table S1).

### IFN-β Is Essential for Neuronal Survival, Neurite Outgrowth, and Branching

We investigated whether *Ifnb*^*–/–*^ behavioral deficits were associated with neurodegeneration. Apoptotic cells were detected in 1.5-month-old *Ifnb*^*–/–*^ granular layers of olfactory bulbs (Figure S1E), the granular dentate gyrus of hippocampus and the subventricular zone (Figure 1G), and the striatum (STR) caudate putamen including the ependymal cell layer in 12-month-old *Ifnb*^*–/–*^ mice (Figure S1F), but not detected in *Ifnb*^*+/+*^ sagittal brain sections at similar ages. Apoptotic neurons increased in cultured *Ifnb*^*–/–*^ primary cerebellar granular neurons (CGNs) and was reversed by recombinant (r)IFN-β (Figures S1G and S1H). Neurons were significantly reduced in the hippocampal CA1 region in 3- to 6-month-old *Ifnb*^*–/–*^ mice and decreased in Purkinje cells of cerebellum. Glial cell counts were unchanged (Figures 1H and 1I).

Supporting in vivo deficits in neuronal circuits, *Ifnb*^*–/–*^ mice had reduced neurite network formation in the cerebellum, frontal cortex, and hippocampus granular cell layer (Figure 1J). This was confirmed in cultured *Ifnb*^*–/–*^ primary cortical neurons (CNs) that had reduced neurite length per cell, mean length, and branch length per cell after 4 days, with restoration by rIFN-β (Figures 1K and 1L). A similar trend was seen after 21 days of culture (Figure 1K). Cultured primary *Ifnb*^*–/–*^ CGNs mimicked the findings of CNs (Figures S1I and S1J). Thus, behavioral defects in old *Ifnb*^*–/–*^ mice were associated with neuron death and reduced neurite circuits.

### *Ifnb*^*–/–*^ Neuron Gene Profiling Reveals Neurodegenerative Paths

**Figure 2:**
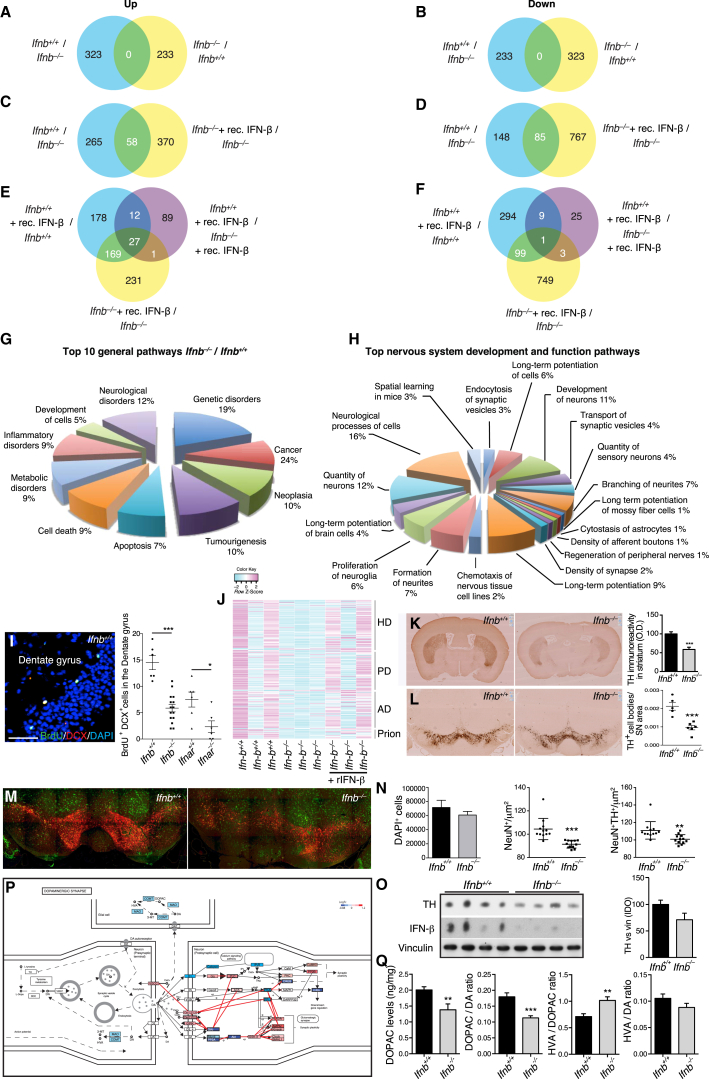
Gene Profiling of *Ifnb*^*–/–*^ Neurons Shows Association with Neurodegenerative Diseases Mouse Affymetrix 430 2.0 arrays. (A–F) Venn diagrams for gene expression analysis of *Ifnb*^*–/–*^, *Ifnb*^*+/+*^ CGNs with or without rIFN-β, n = 3. Genes differentially regulated by 1.5-fold, *^∗^*p < 0.05. (A, C, and E) Upregulated. (B, D, and F) downregulated genes. (G and H) Gene expression analyzed for (G) top ten general signaling pathways and (H) nervous system development and function pathways. (I) Neurogenesis in hippocampus dentate gyrus showing IF of an *Ifnb*^*+/+*^ mouse and quantifying all groups: n = 4 mice/group and six to 12 sagittal sections were counted for BrdU^+^DCX^+^ neurons. (J) GSEA Heatmaps of core-enriched pathways; HD, PD, AD, and prion disease pathways were the top four neurologic disease pathways without or with rIFN-β, n = 3. (K and L) IHC TH staining of coronal (K) STR and (L) SN sections. Data are (K) mean optical density (OD) ± SEM and (L) TH^+^ cell bodies per SN area ± SEM, n = 4 mice. (M and N) Stitched IF images showing NeuN (green) and TH (red) (M) immunoreactivity and (N) quantifications of n = 4 mice/group in three to four ventral midbrain regions. (O) WB of BG from 8-month-old mice (n = 4). Graph, mean integral optical density (IOD) ± SEM of TH bands. (P) Overview of the DA system. Red, upregulated; blue, downregulated genes from comparative microarray analysis of *Ifnb*^*+/+*^ and *Ifnb*^*−/−*^ CGNs. (Q) High-performance liquid chromatography (HPLC) analysis of DA, DOPAC, and HVA in STR of 3- to 6-month-old mice, n = 9–10/group. For (I), (K), (L), (N), and (Q), ^∗^p < 0.05, *^∗∗^*p *<* 0.01, ^∗∗∗^p < 0.001, by Student’s t test. See also Figure S2 and Data S1.

**Figure S2:**
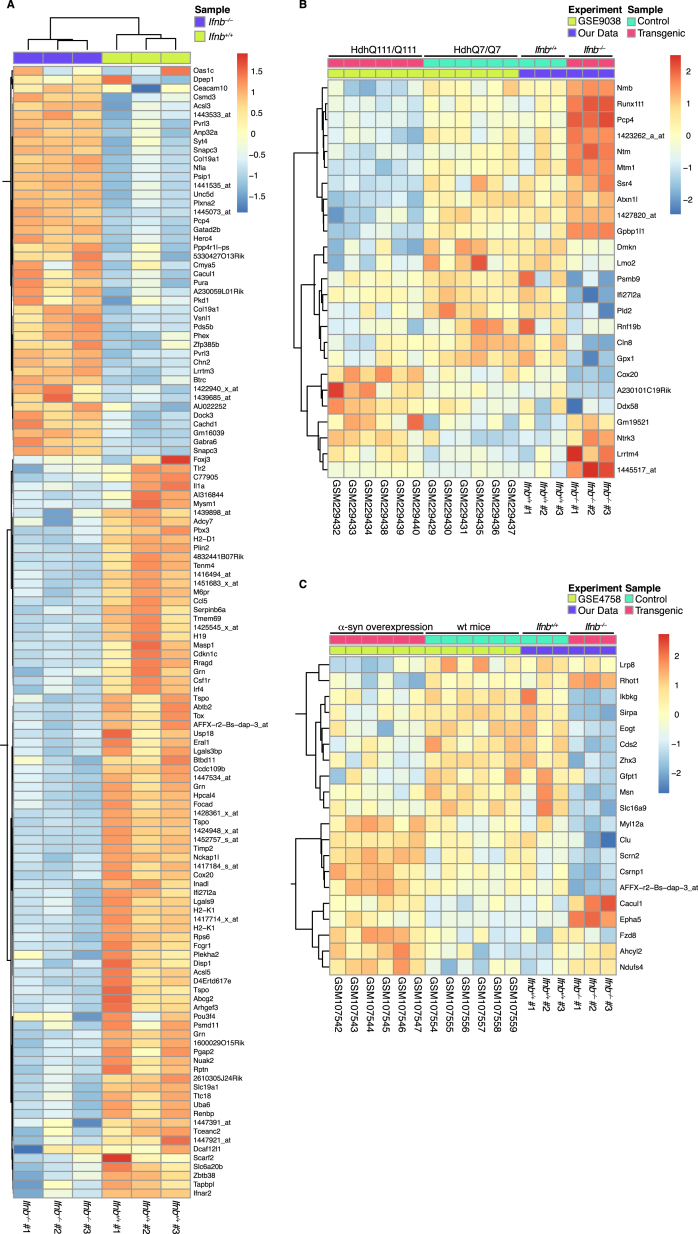
Gene Signature of *Ifnb*^*–/–*^ Neurons and Heatmaps Comparing *Ifnb*^*–/–*^ Mice with Mice Models of Huntington’s and Parkinson’s Disease, Related to Figure 2 (A) Heatmap of genes differentially expressed in *Ifnb*^*–/–*^ and *Ifnb*^*+/+*^ cerebellar granular neurons (CGNs). (B and C) Heatmaps comparing differentially expressed genes in *Ifnb*^*–/–*^ and *Ifnb*^*+/+*^ CGNs with (B) Huntington’s disease affymetrix data (GSE9038) and (C) Parkinson’s disease affymetrix data (GSE4758). Identification of differential expressed genes was performed individually within each experiment (in (A) p < 0.001; in B-C) p < 0.01); n = 3-5.

We identified neuron-specific signaling and disease pathways caused by IFN-β deficiency with expression microarrays on highly pure (>98%) primary CGNs from *Ifnb*^*–/–*^ and *Ifnb*^*+/+*^ mice plus or minus rIFN-β. *Ifnb*^*–/–*^ neurons had 323 upregulated and 233 downregulated genes (Figures 2A, 2B, and S2A). rIFN-β treatment had differential effects on *Ifnb*^*+/+*^ versus *Ifnb*^*–/–*^ CGNs with approximately equal numbers of downregulated and upregulated genes, but with relatively few overlaps (Figures 2C–2F). Multiple genes in the *Ifnb*^*–/–*^ data set annotated as neuronal degeneration and the top ten pathways included cell death and neurological disorders. Genes in neurite formation and branching suggested compromised neurogenesis pathways in *Ifnb*^*–/–*^ neurons, supporting the in vitro and in vivo phenotypes (Figures 2G and 2H). To investigate whether *Ifnb* and signaling via IFNAR affected neurogenesis in vivo, 3-month-old *Ifnb*^*–/–*^ and IFNAR knockout (KO) mice (*Ifnar*^*–/–*^) and WT littermates were injected with bromodeoxyuridine (BrdU). Lack of *Ifnb* or receptor was associated with reduced neurogenesis in the hippocampus; dentate gyrus (Figure 2I), supporting the microarray data. IFN-β might directly affect prosurvival mechanisms as neurotrophin was among the top hits in gene set enrichment analysis (GSEA) (Table S2) and mRNA for the corticogenesis-regulating transcription factor Tox (Artegiani et al., 2015) was reduced in *Ifnb*^*–/–*^ neurons (Figure S2A). Increased apoptosis and reduced neurogenesis might contribute to impaired cognition and reduced hippocampal neuron numbers. GSEA of *Ifnb*^*+/+*^ and *Ifnb*^*–/–*^ showed enrichment of genes associated with Huntington’s disease (HD), PD, AD, and prion diseases, and rIFN-β caused the *Ifnb*^–/–^ profile to resemble WT (Figure 2J), supporting that common pathways link neurodegenerative diseases (Shulman and De Jager, 2009).

We compared the *Ifnb*^*–/–*^ neuronal gene profile with PD and HD mouse models (Fossale et al., 2011). Three sets of differentially expressed genes were generated: differentially expressed in our data (p < 0.001), in our data and the HD model (p < 0.01), or our data and the PD model (p < 0.01) (Figures S2A–S2C; Data S1). Clustered genes in *Ifnb*^*–/–*^ neurons and the HD model were both similarly and oppositely regulated. In contrast, most clustered genes in *Ifnb*^*–/–*^ neurons and the PD model were regulated similarly, thus indicating a higher gene signature resemblance with PD than HD (Figures S2B and S2C).

### Lack of *Ifnb* Causes Defects in the Nigrostriatal Dopaminergic Pathway

Based on the possible PD-related defect in *Ifnb*^*–/–*^ neurons, we investigated nigrostriatal region integrity. Tyrosine hydroxylase (TH)^+^ fiber density and TH^+^ (dopamine-producing) neurons were significantly reduced in the STR and substantia nigra (SN) in *Ifnb*^*–/–*^ mice versus WT (Figures 2K and 2L). NeuN^+^ and NeuN^+^TH^+^ cells were reduced in the ventral midbrain, which was correlated with reduced TH protein in basal ganglia (BG) in *Ifnb*^*–/–*^ mice while total cells were unaffected (Figures 2M–2O). Among differentially expressed genes in *Ifnb*^*–/–*^ neurons, 31 (p = 6.937e-4) were involved in regulating dopamine (DA) signaling (Figure 2P), which is involved in coordinating movements. DA signaling was also defective in the STR. No major differences were found in levels of DA or its metabolite homovanillic acid (HVA) (data not shown) in 3- to 6-month-old *Ifnb*^*–/–*^ mice, but the DA metabolite dihydroxyphenylacetic acid (DOPAC) was significantly lower, thus significantly affecting DA/DOPAC and DOPAC/HVA ratios (Figure 2Q). These findings support the importance of IFN-β in regulating dopamine turnover and protecting dopaminergic neurons.

### Lack of Neuronal IFN-β-IFNAR Signaling Causes Lewy Bodies

**Figure 3:**
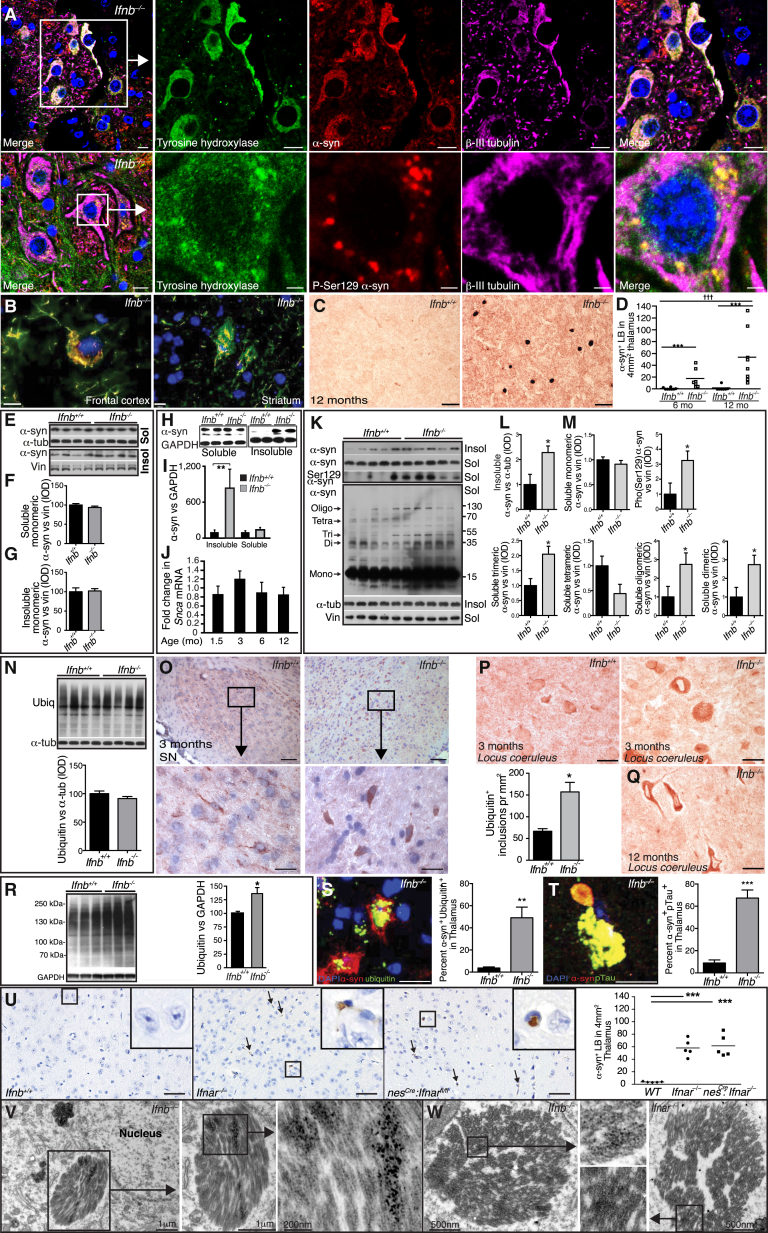
*Ifnb*^*–/–*^ Mice Develop LBs with α-syn, Ubiquitin, and pTau (A) IF of α-syn in SN of 6-month-old *Ifnb*^*–/–*^ mice. Scale bar, 10 μm; 2 μm in close-up lower panel. (B) IF of 3-month-old *Ifnb*^*–/–*^ brains showing α-syn^+^ neuron in frontal cortex and positive aggregates in STR. Scale bars, 10 μm. (C) IHC of α-syn in thalamus. Scale bar, 20 μm. (D) Number of α-syn-positive LBs in thalamus of 6- and 12-month-old mice. ^∗∗∗^p < 0.001 by Mann-Whitney U test for two groups, ^†††^p < 0.001 by Kruskal-Wallis test for all groups. (E–G) (E) WB of α-syn and quantified IOD in (F) soluble and (G) insoluble BG fractions of 1.5-month-old mice. Data are mean ± SEM, n = 4. (H and I) (H) WB of α-syn and (I) quantified IOD in TX-100 soluble and insoluble fractions of 3-month-old brains. Data are mean ± SEM, n = 5. (J) RT-PCR of α-syn in *Ifnb*^*–/–*^ relative to *Ifnb*^*+/+*^ brains. Data are mean ± SEM, n = 3–5. (K) WB of α-syn (TX-100 soluble and insoluble) from BG of 8-month-old mice and long exposure of whole membranes of soluble fractions. (L and M) Quantified IOD of α-syn and pSer129-α-syn in (L) insoluble and (M) soluble fractions of monomeric (short exposure) and high-molecular-weight α-syn species (long exposure). Graphs, mean ± SEM, n = 5. (N) WB and quantified ubiquitin IOD in BG from 1.5-month-old mice. Data are mean ± SEM, n = 3. (O) IHC of ubiquitin in SN at 3 months and quantified ubiquitin^+^ aggregates/μm^2^. Scale bar, 200 μm; 20 μm in inserts. Graph, mean ± SEM, n = 5. (P and Q) IHC of ubiquitin in locus coeruleus at (P) 3 months and (Q) 12 months. Scale bar, 200 μm. (R) WB and quantified ubiquitin IOD in brain extracts from 3-month-old mice. Data are mean ± SEM, n = 3. (S and T) IF and quantification of (S) ubiquitin^+^α-syn^+^ and (T) p-tau^+^α-syn^+^ aggregates in thalami of 12-month-old mice. Scale bar, 10 μm; data are percentage double positives ± SEM. (U) IHC of α-syn in mice thalami showing LB-like structures (arrows). Scale bar, 50 μm. Graph, α-syn^+^ LB-like structures in thalamus ± SEM, n = 5. ^∗∗∗^p < 0.001 by one-way ANOVA and Turkey’s post hoc correction test. (V and W) Immuno-EM of thalamic neurons from *Ifnb*^*–/–*^ and *Ifnar*^*–/–*^ mice showing LB-like structures positive for α-syn by (V) immuno-DAB reactivity and (W) immunogold labeling. For (I), (L), (M), (O), (R), (S), and (T), ^∗^p < 0.05, ^∗∗^p < 0.01, ^∗∗∗^p < 0.001 by Student’s t test. See also Figure S3.

**Figure S3:**
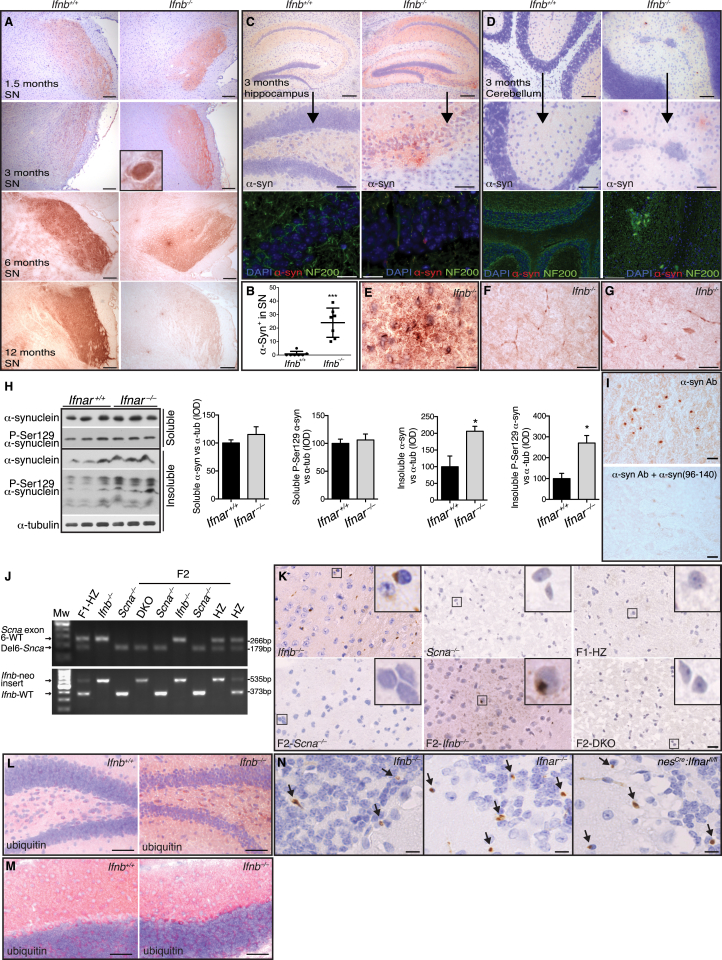
Lack of IFN-β Signaling Causes Widespread α-syn Accumulation in the Brain, Related to Figure 3 (A) α-syn immunostaining (IHC) of snap frozen brains from *Ifnb*^*+/+*^ and *Ifnb*^*–/–*^ showing images of substantia nigra (SN) at 1.5, 3, 6 and 12 months of age. Scale bar, 100 μm. (B) Quantification of α-syn aggregates in *Ifnb*^*+/+*^ and *Ifnb*^*–/–*^ SN at 3 months. (C and D) α-syn IHC staining (upper panels) and IF (lower panels) for α-syn (red), NF200 (green) and DAPI (blue) in hippocampus and cerebellum of *Ifnb*^*+/+*^ and *Ifnb*^*–/–*^ at 3 months. Scale bar, 100 μm; upper panels, and 20 μm; lower panels IHC and IF. (E) α-syn aggregates in thalamus of 3 months old *Ifnb*^*–/–*^ mice. Scale bar, 10 μm. (F and G) α-syn^+^ neurites in brainstem and subthalamic region, respectively. Scale bar, 10 μm. (H) WB (TX-100 soluble and insoluble) from basal ganglia of 8-month-old *Ifnar*^*+/+*^ and *Ifnar*^*–/–*^ mice, and quantified integrated optical density (IOD) of α-syn and phosphorylated (Ser129) α-syn normalized to α-tubulin. Graphs represents mean ± SEM, n = 3. (I) 12 months old *Ifnb*^*–/–*^ thalamus stained for α-syn and lower panel stained with excessive α-syn_96-140_ peptide to block binding of the two utilized antibodies toward α-syn; i.e., sheep anti-mouse (Millipore) (I) and rabbit anti-α-syn (Abcam) (not shown). Scale bar, 20 μm. (J) Genomic PCR screening of mice for *Scna* and *Ifnb* genes. (K) IHC images showing α-syn staining of snap-frozen brain cryosections of parental; *Ifnb*^*–/–*^, *Scna*^*–/–*^, F1 Heterozygots (F1-HZ) and F2; DKO (*Ifnb*^*–/–*^*Scna*^*–/–*^ mice), *Ifnb*^*–/–*^, and *Scna*^*–/–*^ littermates. Scale bar, 10 μm. (L and M) Ubiquitin IHC of snap frozen brains from *Ifnb*^*+/+*^ and *Ifnb*^*–/–*^ showing hippocampus (L) and cerebellum (M) at 3 months of age. Scale bar, 100 μm. (N) IHC images of perfused and paraffin-embedded brain sections showing α-syn positive staining (yellowish brown) in cerebellar molecular and granular layers (blue hematoxyline counter staining) of *Ifnb*^*–/–*^, *Ifnar*^*–/–*^ and *nes*^*Cre*^*:Ifnar*^*fl/fl*^ mice. Scale bar, 10 μm. For (B) and (H) ^∗^p < 0.05 and ^∗∗∗^p < 0.001 by unpaired Student’s t test.

Since *Ifnb*^*–/–*^ mice defects overlapped with neurodegenerative diseases, particularly PD, we examined *Ifnb*^*–/–*^ brain pathology. Gross anatomy was unchanged, but histological examination showed that *Ifnb*^*–/–*^ neuron degeneration was associated with age-dependent α-synucleinopathy. Staining for α-syn was normal in 1.5-month-old *Ifnb*^*–/–*^ brains; by 3 months, α-syn was found in LB-like structures in SN; however, α-syn staining intensity was reduced in 12-month-old *Ifnb*^*–/–*^ mice (Figures S3A and S3B), likely reflecting degeneration of TH^+^ neuron (Figure 2L). Alpha-syn and large pathogenic aggregates of phosphorylated (pSer129) α-syn were found in TH^+^ DA neurons of SN (Figure 3A). At 3 months, α-syn aggregates were widespread in the STR, frontal cortex (Figure 3B), hippocampus, and cerebellum (Figures S3C and S3D). Alpha-syn^+^ aggregates and neurites were found sporadically in thalamus, the brainstem, and subthalamic regions of 3-month-old *Ifnb*^*–/–*^ mice (Figures S3E–S3G). Neurons with α-syn^+^ LB-like structures increased with age (6- and 12-month-old) in *Ifnb*^*–/–*^ mouse thalamus (Figures 3C and 3D). Whole-brain protein extracts from 1.5-month-old mice were not different from WT, but 3-month-old mice had significantly increased α-syn in insoluble fractions and no difference in soluble fractions (Figures 3E–3I). No difference in mRNA for α-syn was observed between *Ifnb*^*–/–*^ and WT brains (Figure 3J); thus, the α-syn accumulation was not due to increased transcription. The insoluble α-syn fraction in the BG (including SN) was significantly higher in older mice, but the soluble fraction was still unchanged. A significant increase in high-molecular-weight dimeric, trimeric, and oligomeric α-syn were seen in *Ifnb*^*–/–*^ mice, but with a decrease in tetramers. While aggregated α-syn oligomers are neurotoxic (Rockenstein et al., 2014), α-syn tetramers are suggested to be the normal aggregation resistant conformation of the protein (Bartels et al., 2011; Wang et al., 2011), possibly explaining the tetramer abundance in *Ifnb*^*+/+*^ mice. pSer129-α-syn, which is prone to form pathogenic fibrillar aggregates, was increased in *Ifnb*^*–/–*^ mice (Figures 3K–3M). *Ifnar*^*–/–*^ brains showed a similar pattern to *Ifnb*^*–/–*^ regarding α-syn and pSer129-α-syn accumulation (Figure S3H). Blocking with α-syn_96–140_ peptide confirmed specific immunoreactivity for α-syn in *Ifnb*^*–/–*^ brains. To ensure that lack of *Ifnb* did not generate crossreacting α-syn-independent aggregates, we generated *Scna*^*–/–*^*Ifnb*^*–/–*^ double-KO mice (DKO). *Ifnb*^*–/–*^ but not *Scna*^*–/–*^ or DKO mice had α-syn^+^ aggregates or LBs (Figures S3I–S3K).

Accumulated polyubiquitinated proteins are associated with neurodegenerative disease (Davies et al., 1997; Hara et al., 2006; Komatsu et al., 2006). While 1.5-month-old WT and *Ifnb*^*–/–*^ mice showed not differences, 3-month-old *Ifnb*^*–/–*^ brains had increased polyubiquitin (Figures 3N–3R), in hippocampus and cerebellum (Figures S3L and S3M), SN and locus coeruleus (Figures 3O and 3P) and vestibular nuclei of pons (not shown). Ubiquitin accumulated with inclusion body abundance, as seen in the locus coeruleus of 12-month-old *Ifnb*^*–/–*^ mice (Figure 3Q) and α-syn^+^ aggregates colocalizing with ubiquitin and phosphorylated Tau (pTau) significantly increased (Figures 3S and 3T).

To exclude systemic immune response influences, we analyzed *Ifnar*^*–/–*^ and *nes*^*Cre*^*:Ifnar*^*fl/fl*^ mice. Consistent with *Ifnb*^*–/–*^ mice, α-syn^+^ LB-like structures were found in the cerebellum, granular and molecular layers (Figure S3N), and significantly in thalamus of *Ifnar*^*–/–*^ and *nes*^*Cre*^*:Ifnar*^*fl/fl*^ mice (Figure 3U). The ultrastructure of *Ifnb*^*–/–*^ and *Ifnar*^*–/–*^ neurons in paratenial and central medial thalamic nuclei contained α-syn-immunoreactive perinuclear LB-like structures (Figures 3V and 3W), which were absent in *Ifnb*^*+/+*^ mice (data not shown), underscoring the importance of endogenous IFN-β–IFNAR signaling in preventing neuronal proteinopathy.

### Lack of IFN-β Affects Autophagy

**Figure 4:**
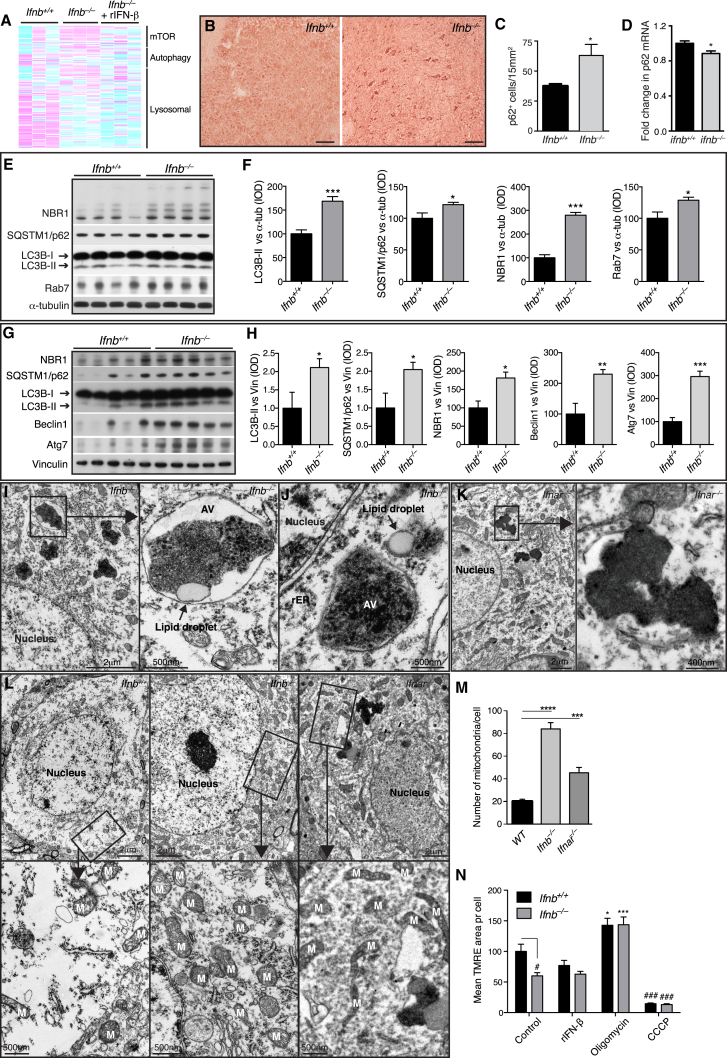
Late-Stage Degradative Autophagosomes Accumulate in *Ifnb*^*–/–*^ Neurons (A) Heatmaps of GSEA core-enriched genes in autophagy-related pathways comparing *Ifnb*^*+/+*^ and *Ifnb*^*–/–*^ mouse CGNs, *Ifnb*^*–/–*^ with or without 24 hr rIFN-β (100 U/ml), n = 3. (B) IHC from pons area using p62 antibodies. Scale bar, 20 μm. (C) Quantification of p62^+^ cells/15 mm^2^. Data are mean ± SEM, n = 5. (D) Fold change in p62 mRNA in brain extracts of 3-month-old mice. Data are mean ± SEM, n = 3. (E–H) WB of BG of (E) 1.5-month-old and (G) 6- to 8-month-old mice and (F) and (H) quantified IOD of bands. Data are mean ± SEM of four or five brains. (I–L) TEM of 9-month-old mice thalami. (I–J) *Ifnb*^*–/–*^ mice showing perinuclear electron-dense late-stage autophagic vacuoles (AV), most surrounded by single lipid membrane and associated with lipid droplets. rER, rough endoplasmic reticulum. (K) *Ifnar*^*–/–*^ mouse with similar electron-dense aggregates. (L) TEM of mitochondria in thalamic neuron cell body. M, mitochondria. (M) Graph, mean ± SEM mitochondria per cell body of 12–16 thalamic neurons; ^∗∗∗^p < 0.001, ^∗∗∗∗^p < 0.0001 by one-way ANOVA with post hoc Dunnett’s multiple comparisons test. (N) TMRE analysis of 21-day-old CNs with mean TMRE area per cell, n = 3. ^∗^Treatment effect, and ^#^genotype effect. For (B), (C), (F), (H), and (N), ^∗^/^#^p < 0.05, ^∗∗^p < 0.01, ^∗∗∗^/^###^p < 0.001 by unpaired Student’s t test. See also Figure S4 and Table S2.

**Figure S4:**
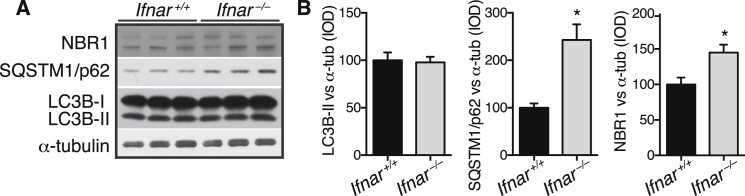
Late-Stage Degradative Autophagosomes Accumulate in *Ifnar*^*–/–*^ Neurons, Related to Figure 4 (A and B) (A) WB of brain extracts from 8-month-old *Ifnar*^*+/+*^ and *Ifnar*^*–/–*^ mice immunoblotted against SQSTM1/p62, LC3B, α-tubulin, and NBR1 and (B) quantified IOD. Data are mean ± SEM of n = 3, ^∗^p < 0.05 by unpaired Students t test.

We used GSEA to identify cellular pathways involved in *Ifnb*^*–/–*^ neuron pathology. In the top 20 deregulated pathways, three were associated with autophagy, which were restored with rIFN-β (Figure 4A; Table S2). The autophagy system uses adaptor proteins SQSTM1/p62 (hereafter p62) and NBR1 to bind ubiquitinated proteins and LC3B-II in the autophagosomal isolation membrane. Upon autophagy, cytosolic LC3B-I is converted to membrane bound LC3B-II and blocking autophagy flux, e.g., with Rab7 defects (Gutierrez et al., 2004; Hyttinen et al., 2013), causes accumulation of autophagy-targeted proteins; p62, NBR1, LC3B-II, and organelles. Increased neurons with accumulated p62 were seen in 3-month-old *Ifnb*^*–/–*^ brains compared to WT, predominantly in brainstem, without increased p62 mRNA. LC3B-II increased in BG of 1.5-month-old *Ifnb*^*–/–*^, correlating with increased p62, NBR1 and Rab7, supporting defects in autophagy before α-syn, ubiquitin, pTau, and LB aggregation. Autophagy was even more deregulated in 6- to 8-month *Ifnb*^*–/–*^ brains (Figures 4B–4H). Defective autophagy was also confirmed in *Ifnar*^*–/–*^ mice (Figure S4).

Senescent or damaged mitochondria are degraded by mitophagy. Large, cytoplasmic, electron-dense aggregates associated with lipid droplets were exclusively found in *Ifnb*^*–/–*^ and *Ifnar*^*–/–*^ thalamic neurons, which correlated with significantly more mitochondria than in WT (Figures 4I–4M). Cultured *Ifnb*^*–/–*^ neurons had significantly lower mitochondrial membrane potential (MMP) than *Ifnb*^*+/+*^ neurons, indicating senescent or damaged mitochondria. MMP was not affected by rIFN-β contrary to positive and negative regulators (Figure 4N). Thus, IFN-β signaling was important in regulating autophagy flux including neuronal mitophagy.

### Late-Stage Autophagy Block Causes α-syn Accumulation

**Figure 5:**
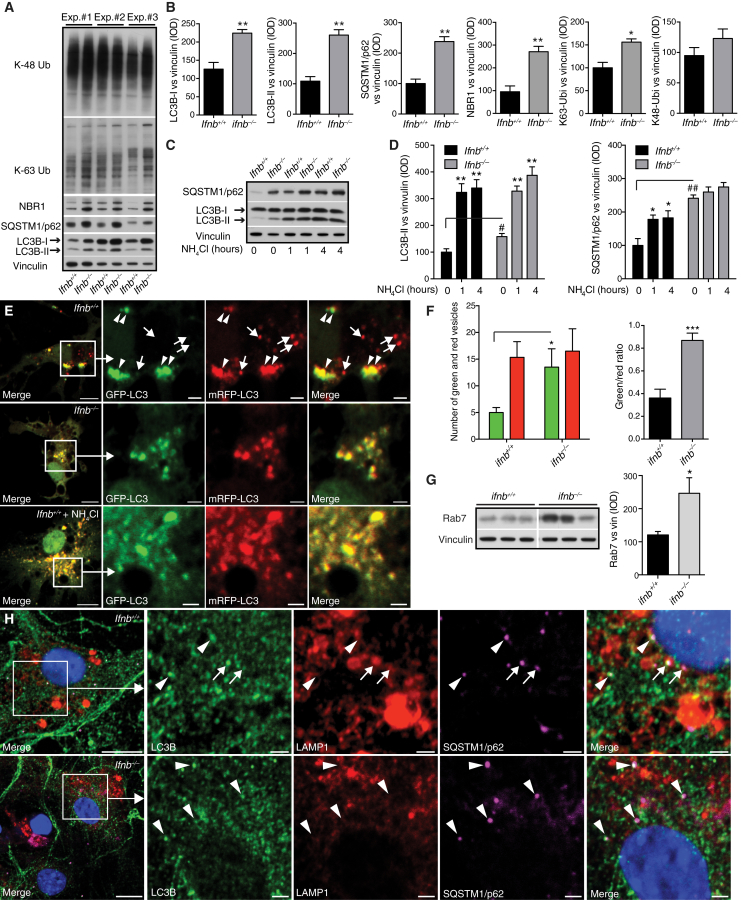
*Ifnb*^*–/–*^ Neurons Have a Defect in Autophagy Maturation (A–H) *Ifnb*^*+/+*^ and *Ifnb*^*–/–*^ primary CNs cultured for 21 days. (A) WB from three independent experiments with antibodies as indicated. (B) Quantified IOD of WB bands normalized to vinculin. Data are mean ± SEM, n = 3. (C) WB of CNs with or without NH_4_Cl (20 mM) for 1 or 4 hr. (D) Quantified IOD of WB. Data are mean ± SEM of n = 3, ^∗^/^#^p < 0.05, ^∗∗^/^##^p < 0.01 by one-way ANOVA. ^∗^Within and # between genotype differences after NH_4_Cl treatment. (E) CNs expressing mRFP-GFP-LC3B with or without NH_4_Cl for 2 hr. Arrowheads, colocalized GFP and mRFP (autophagosomes); arrows, mRFP-only vesicles (autolysosomes). Scale bars, 10 μm; 2 μm in inserts. (F) Graphs, mean number of autophagosomes and autolysosomes/cell and vesicle ratio ± SEM, n = 3. (G) WB of Rab7. Graph, IOD of WB. Data are mean ± SEM of n = 3. (H) IF showing LC3B and p62 colocalized in autophagosomes (arrowheads) and LC3B, LAMP1, and p62 colocalizing in autolysosomes (arrows). Scale bars, 10 μm; 2 μm in inserts. For (B), (F), and (G), ^∗^p < 0.05, ^∗^p < 0.01, ^∗∗∗^p < 0.001 by unpaired Student’s t test. See also Figure S5.

**Figure S5:**
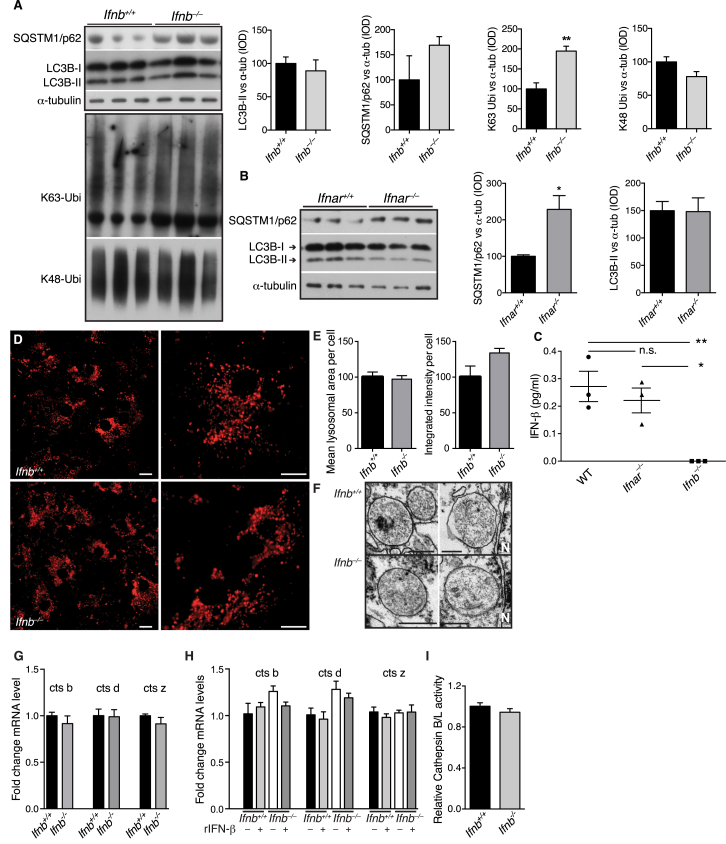
Lysosomes Do Not Change Morphology nor Activity in *Ifnb*^*–/–*^ Mice, Related to Figure 5 (A) WB of CGN from *Ifnb*^*+/+*^ and *Ifnb*^*–/–*^ mice immunoblotted against SQSTM1/p62, LC3B, α-tubulin, K63- and K48-linked ubiquitin. Graphs show mean integrated optical density (IOD) ± SEM, n = 3 of WB bands normalized to α-tubulin. (B) WB of cultured CGNs from *Ifnar*^*+/+*^ and *Ifnar*^*–/–*^ mice. Graphs show mean IOD ± SEM, n = 3. In (A) and (B) ^∗^p < 0.05, ^∗∗^p < 0.01 by unpaired Student’s t test. (C) ELISA analysis of secreted IFN-β in the culture supernatant from WT, *Ifnar*^*–/–*^, and *Ifnb*^*–/–*^ cortical neurons (CN). Graph shows mean pg/ml ± SEM of n = 3 independent CN cultures where each sample was analyzed in triplicates. ^∗^p < 0.05 and ^∗∗^p < 0.01 by one-way ANOVA. (D) Live cell fluorescence images of 21-day-old cortical neurons from *Ifnb*^*+/+*^ and *Ifnb*^*–/–*^ mice loaded with LysoTracker DND-99. Scale bar, 10 μm. (E) Quantified mean area of LysoTracker DND-99 per cell and integrated intensity per cell. Data represent mean ± SEM of n = 2. (F) Transmission electron microscopy images showing lysosomes in neurons from thalamus of 9-month-old *Ifnb*^*+/+*^ and *Ifnb*^*–/–*^ mice. Scale bar, 200 nm. (G and H) RT-PCR of cathepsin b, d, and z showing fold changes in ΔCt of *Ifnb*^*+/+*^ and *Ifnb*^*–/–*^ obtained from (G) brains or (H) CGN’s with or without treatment with rIFN-β (100 U/ml) for 24 hr. (I) Relative activity of cathepsin B and L in CGN’s from *Ifnb*^*+/+*^ and *Ifnb*^*–/–*^ mice. (G–I) Data represent mean ± SEM of n = 3.

*Ifnb*^*–/–*^ CNs and CGNs and *Ifnar*^*–/–*^ CNs showed autophagy defects resembling a late-stage autophagy block (Figures 5A, 5B, S5A, and S5B). In *Ifnb*^*–/–*^ CNs, LC3B-I and LC3B-II were increased with p62, NBR1, and K-63-linked ubiquitin, while K-48-linked ubiquitin only increased slightly (Figures 5A and 5B). Both *Ifnb*^*+/+*^ and *Ifnar*^*–/–*^ produced detectable IFN-β, unlike *Ifnb*^*–/–*^ neurons (Figure S5C). To validate a block in autophagy flux, fusion between autophagosomes and lysosomes was inhibited with NH_4_Cl. At 1 hr, LC3B-II levels saturated in both genotypes, which correlated with a significant increase in p62 in *Ifnb*^*+/+*^ CNs; however, *Ifnb*^*–/–*^ CNs had nearly saturated p62 levels without treatment, and no significant increase was seen with NH_4_Cl (Figures 5C and 5D). Autophagy flux was measured in CNs with the mRFP-GFP-LC3B construct, which emits mRFP and GFP signal in autophagosomes and only mRFP in autolysosomes because of low pH in the latter. *Ifnb*^*–/–*^ CNs had increased mCherry^+^GFP^+^ autophagosomes and very few autolysosomes compared to WT again suggesting blocked autophagy flux. Treating *Ifnb*^*+/+*^ CNs with NH_4_Cl caused a similar distribution as *Ifnb*^*–/–*^ CNs. Rab7 significantly increased in *Ifnb*^*–/–*^ CNs, suggesting accumulation of mature autophagosomes. In support, fewer LC3B- and p62-positive autophagic vacuoles overlapped with LAMP1, a lysosomal marker, in *Ifnb*^*–/–*^ neurons indicating flux or fusion problems (Figures 5E–5H). GSEA suggested dysregulation in lysosomal genes in *Ifnb*^*–/–*^ neurons (Table S2). However, numbers and dysfunction of lysosomes measured as acidification, morphology, or cathepsin expression and activity did not differ in *Ifnb*^*+/+*^ and *Ifnb*^*–/–*^ CNs and brains (Figures S5D–S5I).

**Figure 6:**
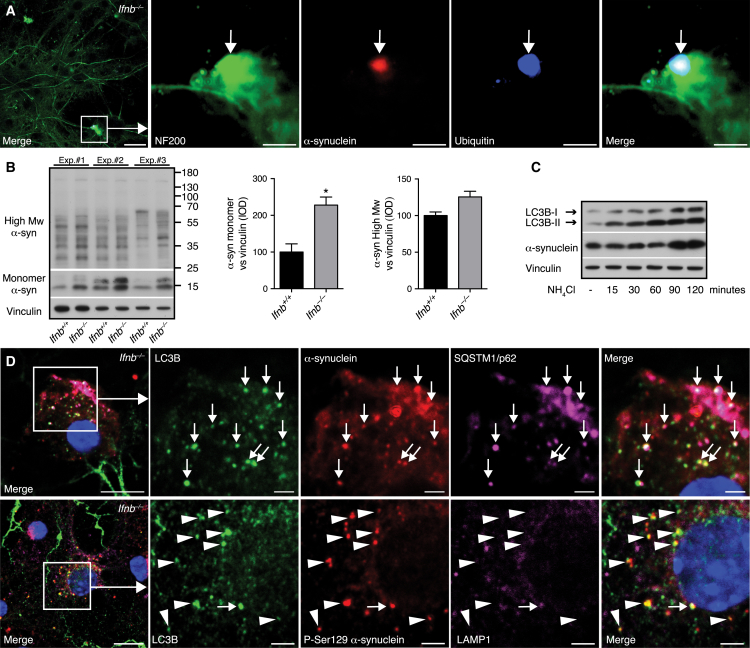
Autophagy Block Causes α-syn Accumulation (A) IF of *Ifnb*^*–/–*^ CN with inclusion body (arrow). Scale bar, 20 μm; 2 μm in insert. (B) WB and quantified IOD bands of monomeric and high-molecular-weight (Mw) α-syn. Data are mean ± SEM, n = 3. ^∗^p < 0.05 by unpaired Student’s t test. (C) WB of *Ifnb*^*+/+*^ CNs treated with 20 mM NH_4_Cl. (D) IF of CNs from *Ifnb*^*–/–*^ mice. Arrows, triple colocalizing vesicular structures; arrowheads, LC3B and phosphorylated (Ser129) α-syn-positive vesicular structures. Scale bars, 10 μm; 2 μm in inserts. See also Figure S6.

**Figure S6:**
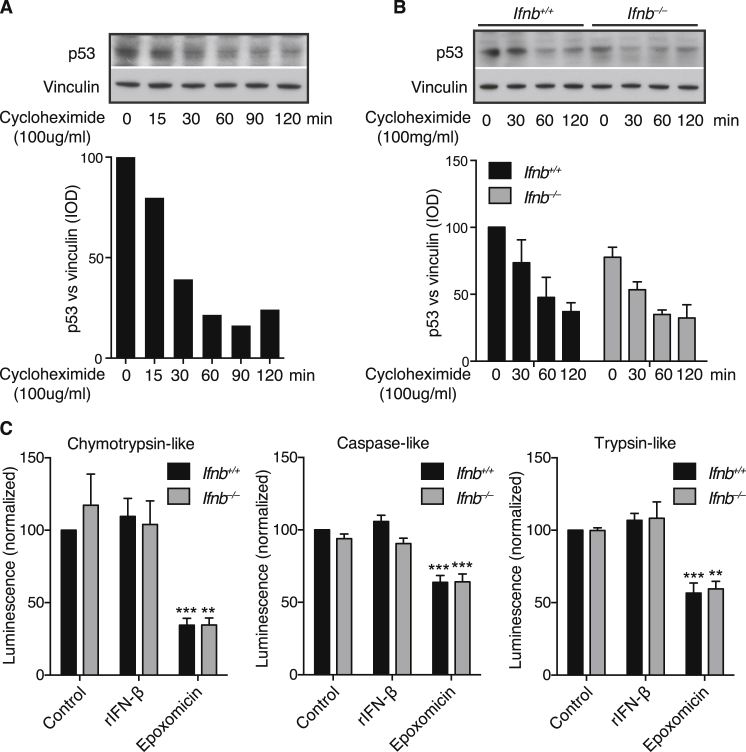
Proteasomal Flux and Activity Are Not Affected by Lack of IFN-β, Related to Figure 6 (A–C) Cortical neurons (CN) cultured for 21 days obtained from *Ifnb*^*+/+*^ and *Ifnb*^*–/–*^ mice. (A-B) WB showing proteasomal kinetic of p53 turn-over in (A) *Ifnb*^*+/+*^ and (B) *Ifnb*^*+/+*^ and *Ifnb*^*–/–*^ CN treated with cycloheximide (100mg/ml) for different time periods as indicated. Graphs show quantified integral optical density (IOD) of p53 WB bands normalized to vinculin and represent (A) one experiment or (B) mean ± SEM of n = 3. (C) Proteasomal Caspase-like, Trypsin-like, and Chymotrypsin-like activity in CN treated with rIFN-β (100 U/ml), epoxomicin (10 μg/ml), or left untreated, measured by relative luminescence units normalized to control. Data represent mean ± SEM of n = 3; ^∗∗^p < 0.01, ^∗∗∗^p < 0.001 by one-way ANOVA.

*Ifnb*^*–/–*^ CNs developed ubiquitin^+^ α-syn aggregates and increased monomeric and high-molecular-weight α-syn after 21 days of culture (Figures 6A and 6B). Accumulation of α-syn was not due to proteasomal defects; turnover of the proteasome substrate p53 after cycloheximide treatment and proteasomal catalytic activity were uncompromised in *Ifnb*^*–/–*^ CNs and unchanged by rIFN-β (Figure S6).

Blocking autophagy flux increased LC3B-II and α-syn, and α-syn colocalized with LC3B^+^ and p62^+^ autophagosomes in *Ifnb*^*–/–*^ CNs (Figures 6C and 6D), underscoring the relevance for autophagic clearance of α-syn. Aggregation-prone pSer129-α-syn was seen in LC3B^+^ autophagosomes, that rarely overlapped with LAMP1. Collectively, these results demonstrated that lack of IFN-β reduced lysosomal fusion and caused α-syn accumulation.

### IFN-β Promotes Neuronal Autophagy and α-syn Clearance

**Figure 7:**
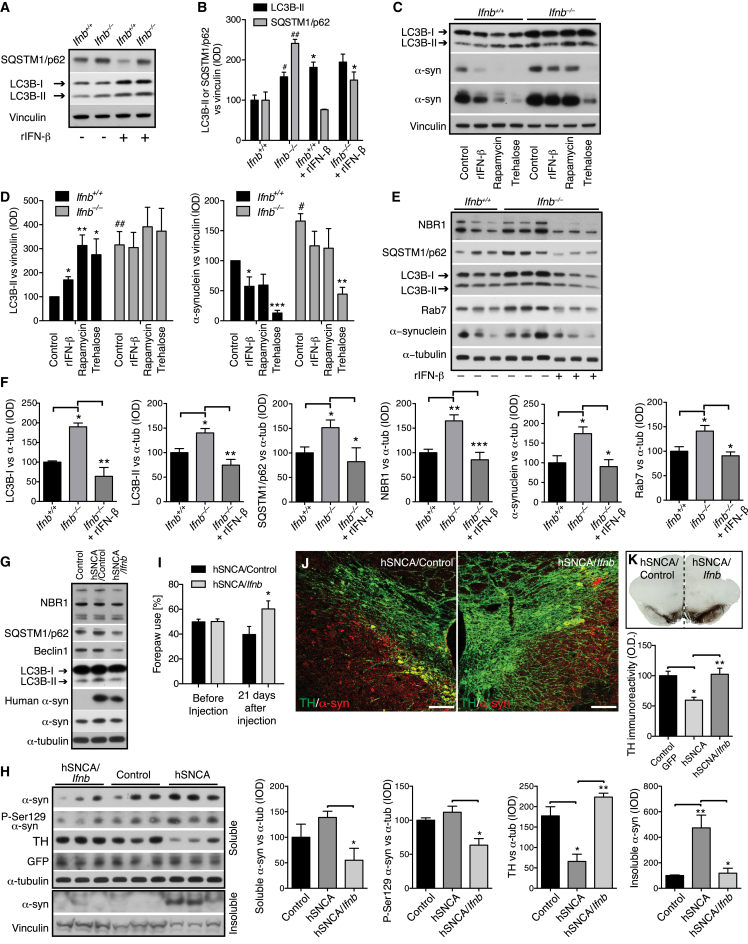
IFN-β Treatment Promotes Autophagy Flux and Reduces α-syn Accumulation (A–F) *Ifnb*^*+/+*^ and *Ifnb*^*–/–*^ primary CNs cultured for 21 days. (A) WB of CN with or without rIFN-β (100 U/ml) for 22 hr. (B) Quantified IOD of WB bands. Graphs, mean ± SEM, n = 3; ^∗^/^#^p < 0.05; ^##^p < 0.01 by one-way ANOVA. ^#^Between genotypes and ^∗^after rIFN-β in each group. (C) WB of LC3, α-syn (two exposures), and vinculin from untreated or rIFN-β (100 U/ml), rapamycin (1 μM), or trehalose (50 mM) -treated CNs (24 hr). (D) Quantified IOD of WB bands with mean ± SEM, n = 4–5. ^∗^After treatment within genotype and ^#^between untreated control genotypes. (E) WB of CNs with 30 U/ml rIFN-β (every 3–4 days) and 100 U/ml the last 24 hr. (F) Quantified IOD. Data are mean ± SEM, n = 4–6. (G and H) WB of rat BG 10 days post AAV- and lentiviral-control or AAV-hSNCA co-injection with lentivirus-*Ifnb* or lentivirus-control vector. Antibodies were against autophagy markers, human α-syn, or human and rat α-syn, pSer129-α-syn, TH, and GFP (to confirm AAV and lentiviral expression). Graphs, mean ± SEM, n = 3/group. (I) Right and left forepaw use in rats before and 21 days after AAV injection of hSNCA with lentivirus-control or lentivirus-*Ifnb* in left and right SN brain hemisphere, respectively. Graph, mean ± SD, n = 3–5 rats/group. ^∗^p < 0.05 by Student’s t test. (J) IF of rat brain; right and left hemisphere 21 days after virus injection. Scale bar, 100 μm. (K) IHC of rat 21 days after virus injection as in (I) with SN TH immunoreactivity. Graph, mean (OD) ± SEM. TH immunoreactivity of control AAV/lentivirus, AAV-hSCNA/lentivirus control, or AAV hSCNA/lentivirus-*Ifnb*, n = 3–5 rats/group. For (B), (D), (F), (H), and (K), ^∗^/^#^p < 0.05, ^∗∗^p < 0.01, ^∗∗∗^p < 0.001 by one-way ANOVA.

LC3B-II and p62 were higher in untreated *Ifnb*^*–/–*^ CNs, and, while overnight rIFN-β-treatment promoted LC3B-II conversion and reduced p62 in *Ifnb*^*+/+*^ CNs, indicating increased autophagy flux, rIFN-β reduced p62 but only slightly increased LC3B-II in *Ifnb*^*–/–*^ CNs. By promoting autophagy, rIFN-β reduced α-syn in both *Ifnb*^*+/+*^ and *Ifnb*^*–/–*^ CNs (Figures 7A–7D). Effects of rapamycin, an mTOR-dependent autophagy activator, were similar to rIFN-β, but the mTOR-independent inducer trehalose more efficiently reduced α-syn in CNs from both genotypes, possibly through increased α-syn secretion (Ejlerskov et al., 2013). To ensure reconstitution of the genetic defect, we cultured *Ifnb*^*–/–*^ neurons with a low rIFN-β dose for 21 days, which increased autophagy flux and α-syn clearance to the level in *Ifnb*^*+/+*^ neurons (Figures 7E and 7F).

### *Ifnb* Gene Therapy Prevents Dopaminergic Neuron Loss in a Familial PD Model

We used lentiviruses to overexpress IFN-β to examine effects on a familial PD model induced with human α-syn (hSCNA) in rat SNs. Injection of hSCNA and control lentiviruses blocked autophagy indicated by accumulated LC3B-II, p62, NBR1, Beclin1, and hSCNA in BG 10 days after SN injection (Decressac et al., 2013) (Figures 7G and 7H). *Ifnb* overexpression prevented hSCNA and pSer129-α-syn accumulation and restored TH loss. The mice showed improved left paw use compared to right paw use; contralateral to injection side of hSCNA/*Ifnb* and hSCNA/control viruses, respectively, 21 days post injection, which was associated with preservation of TH^+^ fibers in SN (Figures 7I and 7J). *Ifnb* gene therapy also significantly protected TH^+^ dopaminergic neurons from hSCNA-induced SN damage (Figure 7K). Thus, IFN-β prevented pathology in a familial model of PD by inducing autophagy and α-syn clearance.

## Discussion

CNS immune activation and inflammation occur in neurodegenerative diseases (Brochard et al., 2009; Lee et al., 2009; Maccioni et al., 2009; Tansey et al., 2008), but their role in initiation is unclear. We report that defects in IFN-β-IFNAR signaling, that is central to immune regulation, trigger neurodegeneration in the CNS of aging mice. IFN-β promotes neurite growth and protein degradation by autophagy. Lack of IFN-β causes neural pathological changes: accumulation of LB-like structures, neural apoptosis, and neurogenesis defects. p62 accumulated in *Ifnb*^*–/–*^ brainstems and BG regions before pathological α-syn aggregation. Aggregates and LB-like structures with α-syn were seen in brainstem and BG including SN, cortex, thalamus, and cerebellum as previously reported in PD and DLB patients (Goedert et al., 2013; Mori et al., 2003).

IFN-β is expressed by neurons to prevent malignant growth (Liu et al., 2013) and neuroinflammation (Liu et al., 2014) and is produced by choroid plexus epithelial cells in aged mouse and human brains without CNS disease (Baruch et al., 2014). In the latter study, injection of an anti-IFNAR antibody in the cerebrospinal fluid, however, positively affected some aspects of cognition. This approach could have complications as injection of full-length antibody might initiate complement-dependent cytotoxicity (Nelson, 2010) and inflammation (Congdon et al., 2013; Linnartz et al., 2012) and cause rapid antibody clearance compared to isotype controls (Sheehan et al., 2006). Genetic generation of *Ifnb* or *Ifnar* knockout mice or specifically targeting neuroectodermal cells in *nes*^*Cre*^*:Ifnar*^*fl/fl*^ mice circumvents such issues. We found that neuronal IFN-β-IFNAR signaling is required for neurons to withstand age-associated pathology. The data also suggest that neuronal IFN-β production in the CNS parenchyma might function differently than high production by choroid plexus epithelial cells or potentially resident microglia.

Familial but not sporadic neurodegenerative diseases are associated with overexpressed or mutant proteins such as α-syn in PD (Thenganatt and Jankovic, 2014). Overexpression of disease-associated proteins (Arima et al., 1999; Jellinger, 2000; Lippa et al., 1998) was not required to accumulate LB-like structures in *Ifnb*^*–/–*^, *Ifnar*^*–/–*^, or *nes*^*Cre*^*:Ifnar*^*fl/fl*^ neurons. *Ifnb* or *Ifnar* deletion was the sole trigger for α-syn-containing inclusion bodies resulting from defective autophagosome maturation. Treatment with rIFN-β promoted autophagy flux and increased α-syn clearance in *Ifnb*^*+/+*^ and *Ifnb*^*–/–*^ neurons, supporting IFN-β function in mutated proteins clearance, as suggested with ataxin 7 (Chort et al., 2013). Shared pathology of *Ifnb*^*–/–*^ and *nes*^*Cre*^*:Ifnar*^*fl/fl*^ mice suggests that the pathogenesis is likely driven by initial changes in neuroectodermal neurons rather than systemic or local immune activation. In agreement, neurons are crucial for regulation of CNS inflammation (Liu et al., 2006).

Protein degradation defects are common in neurodegenerative pathologies (Davies et al., 1997; Hara et al., 2006; Komatsu et al., 2006). We found that endogenous neuronal IFN-β signaling is central in regulating protein degradation by autophagy, including clearing aged mitochondria. Accumulated aged and defective mitochondria may release reactive oxygen species, enhancing neuroinflammation and neuronal death (Chaturvedi and Flint Beal, 2013). Cognitive and motoric impairments in PD is associated with DA dysfunction (Narayanan et al., 2013). Excess cytosolic DA is degraded by monoamine oxidase (MAO) in the mitochondrial outer membrane and MAO defects might cause oxidative stress (Segura-Aguilar et al., 2014). Senescent and damaged mitochondria and reduced MAO mRNA were found in *Ifnb*^*–/–*^ mice and reduced DOPAC in *Ifnb*^*–/–*^ mice supported dysregulation of MAO and mitochondrial genes potentially contributing to neurotoxicity and DA neuron death.

Lack of endogenous IFN-β signaling was associated with spontaneous neurodegeneration, impaired motor coordination and cognition, and neuronal LB-like inclusions with aging as seen in most PD and DLB patients (Jellinger, 2008). Gene profiling supported essential IFN-β regulation of neuronal homeostasis. Neurotrophin was a top hit in GSEA and increased Tox mRNA, important for corticogenesis (Artegiani et al., 2015), supported IFN-β effects on prosurvival mechanisms.

We showed that *Ifnb* gene therapy reversed pathology in a familial PD model, by promoting autophagy and α-syn clearance, which preserved DA neurons and associated neurologic deficit. Our data strongly support an essential role for IFN-β signaling in preventing neurodegenerative pathology and suggest *Ifnb*^*–/–*^ mice as a model for nonfamilial, sporadic neurodegenerative diseases, particularly PD and DLB, with potential for testing future therapies.

## Experimental Procedures

For detailed procedures, see Supplemental Experimental Procedures.

### Mice and Cell Culture

*Ifnb*^*–/–*^ mice (Erlandsson et al., 1998) were backcrossed 20 generations to B10.RIII or C57BL6. *Ifnar*^*–/–*^ and *nes*^*Cre*^*:Ifnar*^*fl/fl*^ mice were in C57BL6 (Prinz et al., 2008). WT were *Ifnb*^*+/–*^, *Ifnb*^*+/+*^ littermates, or *Ifnar*^*+/+*^ C57BL6 mice. C57BL/6JOlaHsd mice (Harlan Laboratories) with a spontaneous deletion of part of *Snca* (α-syn) were crossed with *Ifnb*^*–/–*^ mice for *Scna*^*–/–*^*Ifnb*^*–/–*^. Mice were housed in standard facilities. Sex- and weight-matched mice were used in experiments performed in accordance with the ethical committees in Denmark and approved by our institutional review boards. CGNs were from 6- or 7-day-old cerebella and cortical neuron (CN) cultures from the cortex of 1-day-old mice.

### Behavioral Measurements

Motor-coordination and -learning were evaluated with an accelerating RotaRod (TSE Systems GmbH) automatically recording time before fall. Neuromuscular strength was tested by forelimb hanging time on a bar. Heat and cold tail-pain sensitivity was measured by tail-flick latency time after exposure.

Spatial learning and reference memory were assessed with Morris water maze (Vorhees and Williams, 2006) with slight modifications. Swimming patterns were recorded with Ethovision 3.1 (Noldus Information Technology), measuring the time to reach a hidden platform during learning trials and frequency of platform position crossings during probe tests. Deficits prevalence were calculated as behavioral test scores of *Ifnb*^*–/–*^ mice that deviated from the norm: mean value (SD/2) of the *Ifnb*^*+/+*^ group (Table S1).

In the cylinder tests, asymmetry in forelimb use during vertical exploration was used as a validated measure of akinesia in hemiparkinsonian rodents.

### Cloning

Mouse *Ifnb* pCR4IFNb was from transOMIC (accession no. BC119395). *Ifnb* was transferred to pCSII-GW via pCR8TOPOGW (Invitrogen) with conventional cloning techniques to generate pCSII-IFNb. PCSII (without insert) was generated by lambda recombination with an empty pCR8GW vector. Plasmid inserts were verified by sequencing.

### Surgery with AAV6-hSCNA

AAV-GFP or AAV-human α-syn/hSNCA-WPRE (Vector Biolabs) together with lentiviral vectors pCSII-IFNb and pCSII control (3 μl for each virus) were injected unilaterally into 30 adult female Sprague Dawley rats (Taconic; 225–250 g at surgery). Virus was infused at 0.2 μl/min as described (Decressac et al., 2013).

### Immunohistochemistry, Immunofluorescence, and Transmission Electron Microscopy

For immunohistochemistry (IHC) and immunofluorescence (IF), either mice were perfused and brains fixed in 4% paraformaldehyde (PFA) and paraffin embedded or brains were dissected and snap-frozen before sectioning. In vitro neuronal cultures were fixed in 4% PFA before staining. Tissues and cells were stained as described (Liu et al., 2014).

For neurogenesis and BrdU staining, mice were injected intraperitoneally (i.p.) once per day (75 μg/g body weight) for 5 consecutive days and sacrificed 2 hr after last injection. Brains were processed and immunostained for BrdU and doublecortin (DCX).

MMP was measured by adding tetramethylrhodamine ethyl ester (TMRE) and Hoechst (Life Technologies) to primary CNs.

IF images were taken with a Zeiss LSM510 confocal scanning microscope and IN Cell Analyzer 2200 automated microscope. IHC images were taken with a NanoZoomer 2.0-HT digital slide scanner or Olympus BX51 microscope. Images were quantified with ImageJ (Fiji version), IN Cell Investigator, CellProfiler, Zeiss Zen, and Adobe Photoshop.

In situ apoptosis detection was with TUNEL kits (Calbiochem) with Hoechst counterstaining or DAB substrate and methyl green counterstaining kits (R&D Systems).

For transmission electron microscopy (TEM), 12-month-old mice were cardiac perfused with 2% PFA and 0.2% glutaraldehyde and paratenial, and central medial thalamic nuclei were dissected and processed for epon embedding and ultrathin sectioning. Samples were incubated with primary α-syn antibodies (Leica) and biotinylated (Vector Laboratories) or 1.4-nm gold-labeled secondary antibodies (Nanoprobes) and embedded in epoxy resin. Ultra-thin sections were analyzed in a Philips CM100 electron microscope.

### Transfection and Plasmids

CNs were transfected with mRFP-GFP-LC3 using Lipofectamine 2000 (Life Technologies) according to the manufacturer’s description for live-cell confocal imaging.

### Stereological Analysis

The optical fractionator method was used to estimate neuron and glial numbers in the hippocampus of 80-μm mouse brain sections stained with H&E. Pointcounting techniques, based on the Cavalieri principle, were used to estimate hippocampal volume on one side of the brain.

### Real-Time PCR

Total RNA was isolated using a QIAGEN kit (QIAGEN), reverse transcribed into cDNA, amplified, and quantified by SYBR Green (Bio-Rad) detection. Relative mRNA expression was normalized with glyceraldehyde 3-phosphate dehydrogenase (*Gapdh*) gene.

### High-Performance Liquid Chromatography

Striatum was dissected and homogenized. Filtered supernatant was examined for DA, DOPAC, and HVA levels by reversed-phase HPLCy (Decressac et al., 2013).

### Western Blots

Samples were lysed in 1% Triton X-100 (Sigma) and insoluble brain pellets were sonicated in UREA/SDS and processed as described (Ejlerskov et al., 2013).

### Affymetrix Microarrays

RNA was extracted with TRI (Sigma) and DNase I (Invitrogen) from 3-day-old *Ifnb*^*+/+*^ and *Ifnb*^*–/–*^ CGN cultures in triplicate with or without 24 hr rIFN-β (100 U/ml). Affymetrix 430 2.0 microarray chip (SCIBLU, Affymetrix) data were analyzed with Arraystar 3 (DNA STAR) and quantile-normalized and processed by the RMA (Affymetrix) algorithm. We log_2_-transformed intensity values, and normal-distributed data were tested in unpaired two-tailed Student’s t tests, filtering for differential regulation confidence of 95% (p < 0.05). Venn diagrams were created with oneChannelGUI (Bioconductor). Quantile-normalized RMA-treated data selected using a 1.4-fold cutoff were analyzed with Ingenuity Pathway Analysis software.

GSEA data heatmaps were generated by extracting lists of core enriched genes from GSEA pathway analysis in R using Heatmap2 (Data S1).

Comparisons of *Ifnb*^*–/–*^ CGN gene profiles and published PD and HD models using Affymetrix data (GSE4758, GSE9038) were quantile-normalized together and summarized in R using RMA algorithms from the Affy-package. Differential expression was determined individually within each experiment for our data and published PD and HD models by comparing control/WT samples with transgenic samples using standard ANOVA.

### Statistical Analysis

Data were analyzed with unpaired and paired two-tailed Student’s t tests, ANOVA, and Mann-Whitney U, and Kruskal-Wallis tests. p < 0.05 was significant. Error bars are SEM.

## Author Contributions

P.E., J.G.H., J.W., R.C., M.K., Y.L., G.P., K.K., M.A., C.F.R., and K.R. did experiments and analyzed and prepared data; B.P. contributed to stereological studies; T.G., D.L., and M.P. did experiments including immunolabeling-EM and *Ifnar*^*–/–*^ and *nes*^*Cre*^*:Ifnar*^*fl/fl*^ mouse analysis; D.C.R. contributed material and designed some protein degradation experiments and S.I.-N. designed and supervised the study, analyzed and interpreted data, and wrote the manuscript. All authors read and contributed to the final manuscript.

## Accession Numbers

The accession number for the raw microarray data reported in this paper is Gene Expression Omnibus: GSE63815.

## Supplemental Information

Document S1. Supplemental Experimental Procedures and Tables S1 and S2

Data S1. Gene Profiling of *Ifnb*^*–/–*^ Shows Association with Autophagy and Neurodegenerative Diseases, Related to Figure 2 Core-enriched genes in GSEA pathways of autophagy-related; mTOR, Autophagy and Lysosome, and Neurodegenerative diseases; Huntington’s, Parkinson’s, Alzheimer’s and Prion disease (HPAP) pathways comparing *Ifnb*^*+/+*^ and *Ifnb*^*–/–*^ mouse CGNs, *Ifnb*^*–/–*^ with or without 24 hr rIFN-β (100 U/ml), n = 3.

Document S2. Article plus Supplemental Information
