# Supplementary material for: Lack of Neuronal IFN-β-IFNAR Causes Lewy Body- and Parkinson’s Disease-like Dementia
Source: Cell. 2015 Oct 8;163(2):324–39. doi: 10.1016/j.cell.2015.08.069 (PMC4601085; doi:10.1016/j.cell.2015.08.069)
Supplement: Document S1. Supplemental Experimental Procedures and Tables S1 and S2 [file mmc1.pdf]

Cell
Supplemental Information

Lack of Neuronal IFN-β-IFNAR Causes Lewy Body- and Parkinson's Disease-like Dementia

Patrick Ejlerskov, Jeanette Göransdotter Hultberg, JunYang Wang, Robert Carlsson, Malene Ambjørn, Martin Kuss, Yawei Liu, Giovanna Porcu, Kateryna Kolkova, Carsten Friis Rundsten, Karsten Ruscher, Bente Pakkenberg, Tobias Goldmann, Desiree Loreth, Marco Prinz, David C. Rubinsztein, and Shohreh Issazadeh-Navikas

Supplemental Experimental Procedures

Mice and cell culture

Ifnb$^{-/-}$ mice (Erlandsson et al., 1998) were backcrossed 20 generations to B10.RIII or C57BL6, *Ifnar*$^{-/-}$ and *nes*^{Cre}:*Ifnar*$^{fl/fl}$ C57BL6 mice (Prinz et al., 2008). WT were *Ifnb*$^{+/-}$, *Ifnb*$^{+/+}$ littermates or *Ifnar*$^{+/+}$ C57BL6 mice. Mice were housed in standard facilities. Gender- (males and females) and weight-matched mice were used in experiments. Experiments were performed in accordance with the ethical committees in Copenhagen, Denmark, and approved by the respective Institutional Review Boards.

Cerebellar granular neurons (CGN) were obtained from 6- or 7-day-old cerebella and cultured for three or four days on poly-D-lysine coated culture dishes (Liu et al., 2013; Liu et al., 2014). For cortical neuron (CN) cultures the cortex were dissected from 1-day-old mice pups and processed as previously described (Brewer and Torricelli, 2007). Neurons were cultured on poly-D-lysine coated plates (137,500 cells/cm^2) in Neurobasal medium (Gibco) containing B27 (2%) and gentamicin for 21 days. Half of the medium was change every 3rd or 4th day and for long-term treatment, IFN-β (30U/ml) was added at each change.

Generation of Scna$^{-/-}$Ifnb$^{-/-}$ double knock out mice

To generate double knock out mice, C57BL/6JOlaHsd mice (Harlan Laboratories, The Netherlands) with a spontaneous deletion encompassing part of the α-synuclein (*Snca*) gene (Specht and Schoepfer 2004) were purchased. We intercrossed mice lacking α-synuclein (*Scna*$^{-/-}$) with *Ifnb*$^{-/-}$ mice (F_1/heterozygotes). Next we generated double KO mice (*Scna*$^{-/-}$*Ifnb*$^{-/-}$) by intercrossing F_1 mice and genotyping the F_2 mice (Figure S3J).

Antibodies and reagents

Primary antibodies used; polyclonal rabbit NF200 (Sigma), monoclonal mouse LC3B (Cosmo Bio Co LTD, IF), polyclonal rabbit LC3 (Sigma, WB); monoclonal mouse vinculin (Sigma); monoclonal rabbit K-63-linked ubiquitin (Millipore); polyclonal rabbit K-48-linked ubiquitin (Millipore); polyclonal rabbit SQSTM1/p62 (MBL); monoclonal mouse Beclin1 (BD Transduction lab), polyclonal rabbit NBR1 (Proteintech); polyclonal rabbit Atg7 (ProSci); rat monoclonal LAMP1 (BD Biosciences); monoclonal rabbit α-synuclein [EP1646Y] (AbCam, WB and IF); monoclonal rabbit phosphorylated S129 α-synuclein [EP1536Y] (AbCam); polyclonal sheep α-synuclein (Millipore, IF); monoclonal mouse against human α-synuclein (4B12; ThermoScientific); monoclonal mouse against α-synuclein (NCL-L-ASYN; Leica); polyclonal rabbit tyrosine hydroxylase (Millipore); polyclonal rabbit anti-p53 (CM5) (Leica); monoclonal mouse BrdU (RPN202; GE Healthcare), polyclonal goat Doublecortin (C-18; Santa-Cruz); monoclonal mouse α-tubulin (Sigma); polyclonal rabbit phosphorylated tau (T205) (AbCam); polyclonal rabbit GAPDH [EPR1977Y] (AbCam); monoclonal mouse multi-ubiquitin (Medical and Biological Laboratories); mouse monoclonal ubiquitin (Chemicon); monoclonal mouse NeuN, clone A60 (Millipore).

Secondary antibodies used; biotinylated rabbit anti-sheep antibody (Vector Laboratories) and streptavidin-CY3 (1:200, US Biological), goat fluorescein-coupled anti-rabbit antibody (1:200, BD Pharmingen), goat anti-mouse, -rabbit, -rat, -sheep coupled to Alexa Fluor-488, -568, and -633 (1:1000, Invitrogen). Nuclei were stained with DAPI (1:30.000, DAKO) or Hoechst (1µg/ml, Life Technologies).

The specificity of antibodies against α-synuclein for IHC staining was confirmed by blocking the antibodies using saturating concentration of specific recombinant peptides and analyzing immunoreactivity in brain tissues of *Snca*$^{-/-}$*Ifnb*$^{-/-}$ mice.

Reagents used: Oligomycin, Carbonyl cyanide 3-chlorophenylhydrazone (CCCP), Rapamycin, Trehalose (all from Sigma); cycloheximide, epoxomicin, and Ammonium chloride (NH_4Cl) (all from Merck; Calbiochem); and IFN-β (PBL Biomedical Laboratories).

Behavioral measurements

Groups were sex- and weight-matched. Mice were daily habituated to the experimental arena for 3 days. The experimenter was blind to the genotype of the mice. Motor-coordination and -learning were evaluated in an accelerating RotaRod (TSE Systems GmbH). After pre-training at 4 rpm for one minute (min), the speed was gradually increased from 4 to 40 rpm over 3 min, and kept at 40 rpm for additionally 2 min. Time before falling was automatically recorded with a maximum duration of 5 min. Four trials were performed with at least 10 min rest in between. The 3rd trials (peak performances) were considered for statistical comparisons of retention time / motor-coordination in all ages and groups.

Neuromuscular strength was tested by placing the forelimbs of the mouse on a 40 cm elevated stainless steel bar (2mm in diameter), and subsequently measuring the seconds spent hanging for a maximum of 60 seconds (sec). The graph represents an average of two trials with 2 min rest.

Tail-flick was measured by letting mice voluntarily enter a tapered plastic cylinder with air holes. One third of the free-hanging tail was immersed in 50°C or 0°C water and tail-flick latency was recorded with a maximum of 10 sec at 50ºC, or 20 sec at 0ºC. Four trials were conducted with 3-5 min intervals and graph represents mean of the last three trials performed.

The forced swimming test; the mice were individually forced to swim for 5 min inside a 16 cm in diameter glass cylinder tank containing water about 15 cm deep (the subjected individual was not able to stand on the bottom but its tail was able to reach the bottom of the tank) at 25°C, approximately 2°C above RT. The water was changed between sessions to eliminate odour cues. For evaluation of activity or immobility the mice were observed and movements recorded using a stable camera placed in front of the transparent swimming tank. The read-outs were five different behavioral states, i.e. 1) swimming, i.e. active swimming motions, e.g., moving around in the cylinder, 2) diving, i.e. when the whole body of the mouse, including the head is submersed, 3) climbing, i.e. vigorous movements with the forepaws in and out the water, usually directed against the wall of the cylinder, 4)

immobility, i.e. the lack of motion of the whole body, except for small irregular movements necessary to keep its head above water and 5) complete immobility, i.e. the lack of motion of the whole body. The duration of each behavior was recorded, as well as the latency of immobility, a measure that gives information about which group is first to give up on finding an escape. Also, the numbers of faecal boli deposited in the water tank were counted after every session. Rodents that are relatively inactive and have high defecation scores are regarded as fearful, whereas animals with high activity and low defecation scores are considered to be less fearful. The mice were immediately removed from the water tank after the test, dried with paper towels and allowed to dry and warm up for 15 min in a heated container before being returned to their home cages.

Spatial learning and reference memory was assessed in a Morris Water Maze as described (Vorhees and Williams, 2006) with slight modifications. A white plastic water tank (120 cm diameter, ~60 cm high) was filled with water (25°C ±1°C) and made opaque with powdered milk. A circular platform (10 cm in diameter) was submerged 1 cm below the water surface, in a distance of 30 cm from the rim and center of the maze. An automated video tracking system, Ethovision 3.1 (Noldus Information Technology), recorded the time taken to reach the hidden platform during the learning trials and the frequency of platform position crossings during the probe tests. After habituation to the tank, the procedure entailed two learning trial blocks executed on day 1–4 and 6–8 and two probe trials on day 5 and 9. During learning trials (4 per day), mice were placed at one of the 4 starting points that varied semi-randomly between trials and day. Time taken to find the hidden platform (escape latency) and swimming distance were recorded for a maximum of 60 sec. After each trial the mice were left on the platform for 20 sec or placed there manually if they failed to find it within 60 sec. Subsequently, all mice were dried and kept warm. Trial interval was 20 min ±5 min. During probe trials the platform was removed and the number of platform position crossings (14-cm diameter annulus of the platform center) was recorded. The platform was returned immediately after probe 1 and the mice were allowed to stay on it for 20 sec. Statistical analysis was conducted with GraphPad Prism. Two-way repeated-measure ANOVA with Bonferroni *post-hoc* test was conducted to assess the effects of genotype, learning days, and genotype differences within each learning day (trials). Unpaired and paired Student's *t*-test was used to test for significance between $Ifnb^{+/+}$ and $Ifnb^{-/-}$ mice within the same probe day and between the first and second probe day within each genotype, respectively. Collectively, the prevalence of these deficits was calculated as the behavioral test scores of $Ifnb^{-/-}$ mice deviating from the norm; i.e., mean value – (SD/2) of the $Ifnb^{+/+}$ group.

Cylinder test was conducted by asymmetrical forelimb use during vertical exploration to provide a validated measure of akinesia in hemiparkinsonian rodents.

Cloning

Mouse *Ifnb* (pCR4IFNb)) was obtained from transOMIC (BC119395). *Ifnb* was transferred to pCSII-GW via pCR8TOPOGW (Invitrogen) with conventional cloning techniques to generate pCSII-

IFNb. pCSII- (without insert) was generated by LR-recombination with an empty pCR8GW vector. Plasmid inserts were verified by sequencing.

Lentiviral production

293FT cells (80% confluent) were transfected with 34.4 μg pCSII- or pCSII-IFNb, 11.8μg pCMV-VSV-G-RSV-Rev (RIKEN BRC), and 23.6μg pMDLg/pRRE (Addgene) with lipofectamine 2000, according to the manufacturers protocol (Invitrogen). After 24 and 48 hours the media was harvested and filtered through a 0.45μm filter (Satorius). The virus supernatant was then precipitated with PEG6000 and titers were determined according to production, concentration and titration of pseudotyped HIV-1-based lentiviral vectors (Kutner et al., 2009).

Surgical procedure with AAV6-hSCNA

In total 30 adult female Sprague Dawley rats (225-250 g at the time of surgery) were used (Taconic). For general anaesthesia we used an isofluorane anesthesia (4% for induction, 1.7-1.9% to maintain anesthesia). Rats were placed in a stereotaxic frame (Stoelting) and virus was injected using a 10 μl Hamilton syringe fitted with a glass capillary with an outer diameter of 250 μm. Three microliters of AAV6-GFP or AAV6-human α-synuclein (hSNCA-WPRE; Vector Biolabs) were injected unilaterally in SN, together with lentiviral vectors pCSII-IFNb (over-expression *Ifnb*) or pCSII control and 10 days later SN were processed for WB. Rats used for cylinder test, IF and IHC staining were injected with hSNCA and control lentivirus in the left brain hemisphere and hSNCA and pCSII-IFNb in the right brain hemisphere of the same rat. Solutions were infused at a rate of 0.2 μl/min and the needle was left in place for an additional 1 min period before it was slowly retracted. Injection was carried out above the SN, at the following coordinates (flat skull position): antero-posterior: -5.3 mm, medio-lateral: -1.7mm, dorso-ventral: -7.2 mm below dural surface as calculated relative to bregma according to the stereotaxic atlas.

Immunohistochemistry, immunofluorescence, and electron microscopy

Fresh brain tissue was snap-frozen and cryosectioned (6-10μm) for immunohistochemistry (IHC) and immunofluorescence (IF) as described (Liu et al., 2006). In some experiments, mice were perfused and brain tissues fixed in 4% paraformaldehyde (PFA), dehydrated in ethanol and embedded in paraffin before sectioning. *In vitro* cell cultures and cryosections were fixed in 4% PFA for 10 and 20 min, respectively. *In vitro* cultures, paraffin- and cryosections were incubated in blocking buffer (0.3% TX100, 5% normal goat serum (NGS), and 2% BSA in phosphate buffered saline (PBS)) for 15-30 minutes, and subsequently in blocking buffer containing primary antibodies overnight at room temperature (RT) in a humidified container. The next day cells were washed three times in PBS, incubated in blocking buffer containing Alexa Fluor® 488, -568, and -633 secondary antibodies where appropriate for 1 hour, and subsequently in PBS containing DAPI for 5 min. Finally, the slides were washed three times in PBS, one time in MQ-water, and mounted with ProLong® Gold antifade

reagent (Life technologies) and cover slip.

For neurogenesis analysis mice were injected with BrdU (75µg/g body weight) i.p. once per day for five consecutive days and sacrificed 2 hours after the last injection. The brain slides were incubation in blocking buffer (0.3% TX100, 5% BSA in PBS^-) for 20 min, incubated in 1M HCl, 37°C, for 1 hour, washed 2x5 min in 0.1M sodium borate (pH 8.5), washed 3 times in PBS^- before proceeding with IF staining as above with antibodies against BrdU and doublecortin.

Brain sections for IHC were fixed in either 4% PFA or ice cold acetone for 30 or 5 minutes, respectively, washed in PBS^- and incubated in 3% H_2O_2 for 20 min to quench endogenous peroxidases. Slides were then washed three times in PBS and added blocking buffer (0.3% TX100, 5% NGS, and 2% BSA in PBS) for 30 min, followed by avidin and biotin blocking (Vector labs), and subsequently incubated with primary antibodies over night at 4°C. The following day the slides were washed three times in PBS^- and added IgG specific secondary antibodies coupled to biotin for 1.5 hours at RT, washed three times in PBS^-, and subsequently added ABC peroxidase solution (Vector labs) for 1 hour. DAB solution (10 mg DAB, 0.024% H_2O_2, and 10mM NiCl in 15 ml PBS) or NovaRed was added and when sufficient reaction product had developed the slides were washed 20 min in PBS^-, dehydrated in ethanol and xylene and cover slips mounted with Pertex. In some experiments the slides were stained with hematoxylene (15 seconds) and washed repeatedly in water prior to the dehydration steps.

Confocal microscopy images were acquired with a Zeiss LSM510 confocal laser scanning microscope with a C-Apochromat ×63, 1.4 NA oil immersion objective, using the diode 405 nm, the argon 488 nm, the helium–neon 543 nm and 633 nm laser lines for excitation of DAPI/Hoechst, Alexa Fluor® 488, 568, and 633, respectively. Confocal sections of 0.8-1.0µm were collected and saved as 512x512-pixel or 1024x1024-pixel images at 12-bit resolution before import to Adope Photoshop CS6, Zeiss Zen, or ImageJ for compilation and quantification. For 3D imaging 15-20µm thick sections were superimposed in the z-plane using the Zeiss Zen software.

IHC images were acquired in a NanoZoomer 2.0-HT digital slide scanner or with an Olympus BX51 microscope using ColorView IIIu software. Neurite length, branches and numbers (from superimposed 3D images), and stereological analysis of tyrosine hydroxylase IHC staining of striatum and substantia nigra were quantified in ImageJ (Fiji version) using Simple Neurite Tracer and Analyze Calibrate O.D., respectively, and for the latter a custom made macro for cell body count per area of substantia nigra. IF staining of brain slices with antibodies against TH were imaged in an IN Cell Analyzer 2200 automated microscope and images quantified using the IN Cell Investigator software and stitched together with CellProfiler software from Broad Institute.

Mitochondrial membrane potential was measured by adding TMRE (20 nM) and Hoechst (Life Technologies) to the medium of primary cortical neurons for 20 min at 37°C. Images were acquired with an IN Cell Analyzer 2200 automated microscope, and number of cells and mean TMRE area were analyzed with CellProfiler software. Neurons were either left untreated or treated with rIFN-

β (100U/ml) for 24 hours, CCCP (16 μM) (added together with TMRE), or oligomycin (5 μg/ml) (added just prior to imaging).

Lysosomes were labeled with 50 nM LysoTracker® Red DND-99 (Life Technologies) for 20 min before live cell imaging in a LSM510 zeiss confocal microscope. HEPES (20 mM) was added to the cultures before imaging. Images were acquired with an IN Cell Analyzer 1000 microscope for quantification of area and integrated fluorescence intensity per cell (90-177 cells were imaged per experiment) using CellProfiler software.

Terminal deoxynucleotidyl transferase dUTP nick end labeling (TUNEL) staining was performed with an *in situ* apoptosis detection kit (R&D Systems) with DAB substrate and methyl green counterstaining, or a TUNEL kit (Calbiochem) with Hoechst counterstaining. Nuclei and $TUNEL^+$ cells were counted from images in a blinded manner.

For transmission electron microscopy (TEM) analysis mice were cardiac perfused with PBS^- followed by a phosphate buffer (PB) containing 2% PFA and 0.2% glutaraldehyde, pH 7.4, and the paratenial and central medial thalamic nuclei were dissected from the brain and processed for epon embedding and ultrathin sectioning on a vibratome. For immuno-EM 12-month-old mice were deeply anesthetized and transcardially perfused with 0.9% saline followed by a fixative containing 4% PFA and 0,05% glutaraldehyde in 0.1 M PB, pH 7.4. Brains were removed and postfixed in the same fixative for 24 h. Tissue blocks were washed with PB and horizontal sections (50 μm) were cut on a vibratome. Sections were treated with 3% H_2O_2 for 10 min. After washing in PB, the sections were incubated in blocking solution containing 5% NGS in 0.1 M PB for 1 h, followed by incubation with primary antibodies (1:20 α-synuclein (NCL-L-ASYN; Leica); for 24 h at 4°C. After washing with PB, the appropriate biotinylated (Vector Laboratories) or gold-labeled secondary antibodies (1.4 nm gold, Nanoprobes) were applied to the sections for 24h. Sections incubated with biotinylated antibodies were washed, incubated for 1 h at RT with ABC solution (Vector Elite Kit; Vector Laboratories) and visualized by using peroxidase reaction with DAB (diaminobenzidine) as the substrate. Sections incubated with Gold-conjugated antibodies were treated with HQ Silver™ Enhancement Kit (Nanoprobes). Finally, the sections were treated with OsO_4, stained with uranyl acetate, dehydrated, and flat-embedded in epoxy resin (Sigma). Ultrathin sections were cut and analyzed with a Philips CM100 electron microscope equipped with a digital camera.

Transfection and plasmid

Autophagy flux was evaluated by lipofectamine™ 2000 (Life Technologies) transfecting cortical neurons (on culture day 19) with the mRFP-GFP-LC3 tandem construct (provided by David Rubinsztein) according to manufacturer's description. HEPES (20 mM) was added to neurons on culture day 21 and the culture dish was placed in a preheated (37°C) microscope stage for live cell confocal imaging.

Stereological analysis

The optical fractionator method was used to estimate the total number of neurons and glia in the hippocampus of the brain sections (80 μm) of each mouse stained with H&E. Point counting techniques, based on the Cavalieri principle, were used to estimate the volume of the hippocampus on one side of the brain.

Western blotting

Cells were lysed in lysis buffer (100 mM NaCl, 50 mM Tris-HCl, 1 mM EGTA, 10 mM $MgCl_2$, pH 7.2) containing 1% Triton X-100, phosphatase and protease inhibitor cocktail (Sigma) for 5 min at RT and hereafter kept on ice. Cell lysates were centrifuged at 16,100g for 5 min at 4ºC, and protein concentrations of supernatants were determined with BCA assay (Pierce). Lysates were added 4xLDS containing 100 mM DTT and equal amounts of protein were loaded and separated on 4-12% or 16% SDS-PAGE gels, and transferred to Hybond C extra or PVDF membranes. Membranes were incubated with primary antibodies over night, washed three times in washing buffer (0.1% Tween-20 in PBS), incubated with horse radish peroxidase (HRP) coupled secondary antibodies for 1 hour, washed three times in washing buffer, and developed using chemiluminescent HRP detection substrate (Millipore). When extracting proteins from brain tissue, one half brain (cut sagittally) or the basal ganglia were added lysis buffer (same as above) and homogenized in a cell douncer. The homogenate was then centrifuged two consecutive times at 16.100g for 10 min at 4°C, and the final supernatant was collected and processed as above. The TX-100 insoluble pellets from the two centrifugations steps were lysed in UREA buffer (8M Urea, 5% SDS and 1 mM EGTA) and subsequently sonicated. The cell lysates were then processed as above.

Enzyme-Linked ImmunoSorbent Assay (ELISA)

Supernatant of CN cultures from WT, *Ifnb*$^{-/-}$ and *Ifnar*$^{-/-}$ were collected after 3 days of culture for ELISA. Mouse IFN-β ELISA was performed according to manufacturer (VeriKine-HS^{TM}) mouse IFN-β Serum ELISA kit (Catalog No.42410, PBL assay science). Medium from rIFN-β treated CNs served as a positive control.

Quantitative real time (RT)-PCR

Total RNA was isolated using a total RNA purification kit (RNeasy Plus Mini Kit, 74134, QIAGEN) and reverse transcribed into cDNA (iScript cDNA Synthesis kit, 170-8891, Bio-Rad). qPCR reactions were performed by Maxima™ SYBR Green/ROX qPCR Master Mix (K0002, Fermentas)

Relative mRNA expression was calculated using *Gapdh* and *Actb* (beta-actin) gene as endogenous reference for mouse and rat brain tissue, respectively. The primers were purchased from SABioscience, QIAGEN, as following: Mouse primers: *Snca,* PPM25867E; *Gapdh,* PPM02946E; p62: for (5'-CCT ATA CCC ACA TCT CCC AC-3'); rev (5'-TAC AAG AAT GCC AAG ACA

CTG-3'); CatB: for (5'-CTG CTT ACC ATA CAC CAT CC-3'); rev (5'-ATC TCC TTC ACA CTG TTA GAC-3'); CatD: for (5'-CTG TAT CGG TTC CAT GTA AGT-3'); rev (5'-CCA AGC ATT AGT TCT CCT CC-3'); CatZ: for (5'-CCG TTA TCA ACC ACA TCA TCT C-3'); rev (5'-ATC CTC ATC CAG CCT TTC TC-3').

The following primers were used for genotyping *Snca*$^{-/-}$ mice containing the Del(6)Snca1Slab deletion and *Ifnb*$^{-/-}$ mice containing the neo-insert: *Snca* exon VI 5': AAG ACT ATG AGC CTG AAG CCT AAG; *Snca* exon VI 3': AGT GTG AAG CCA CAA CAA TAT CC; D6Slab17-pA: TTG ATA GTT CCA CTG TTC TGG C; D6Slab17-pB: GTA ACA ATA CAG CAA GAG ATA C; *Ifnb* sense: TAT CTT CAG GGC TGT CTC CTT TCT; *Ifnb* anti-sense: ACC TGT TGT TCA TGA TGG AAG CCA; λ2 anti-sense: GGC ATA GTT ACT AGT TGT AAC AGC.

Determination of Dopamine and its Metabolites by HPLC

The striatal tissues of the rats were dissected using stereotaxic coordination, homogenized in perchloric acid 0.1 N, centrifuged at 14,000 Å~ g for 30 min and 200 µl of the supernatant was filtered through a glass 0.22 µm filter (Avantec; 13CP020AS). Filtered supernatant (20 µl) was examined for dopamine (DA), 3,4-dihydroxyphenylacetic acid (DOPAC), and homovanillic acid (HVA) levels by reversed-phase HPLCy (RPHPLC) with electrochemical detection. Typically, 20 µl sample was injected onto a Prodigy C18 column (100 Å~ 2 mm ID, 3-µm particle size; Phenomenex). The mobile phase consisted of 93% (wt/vol) of 94.2 mM NaH2- PO4, 0.98 mM octanesulfonic acid, 0.06 mM Na2EDTA, adjusted to pH 3.2 with 1M phosphoric acid and 9% acetonitrile (vol/vol). The flow-rate was 0.15 ml/min.

Cathepsin activity

Cathepsin activity was obtained from whole brain lysates. Mice were sacrificed at day 7 and dissected brains were snap-frozen in liquid nitrogen. The tissue was homogenized by 15 firm strokes using a cell douncer in acidic Mantle-Buffer (50 mM NaAcetate, 100 mM NaCl, 1 mM DTT, pH 5.5) (1:10 weight to volume ratio) on ice. Lysates were incubated at 4°C for 15 minutes while rotating. After centrifugation at 10,000 rpm for 15 minutes at 4°C, the supernatants were taken and protein concentration determined. For measurements of cathepsin activity, 50 µl brain lysate was mixed with 50 µl of cathepsin reaction buffer (50 mM NaAcetate, 4 mM EDTA, 8 mM DTT, and 0.5 mM Pefablock) in a black 96-well plate. The cathepsin reaction buffer contained a cathepsin B/L specific substrate Z-phe-arg-AFC (MP Biomedicals). The plate was incubated at 37°C in a FluoStar Optima plate reader and kinetic fluorescence (excitation filter at 400nm and emission filter at 480nm, recording once a minute) was measured for 30-35 minutes. Enzymatic activity was determined by averaging 4-5 time points at the steepest point of the fluorescence slope. For each animal the assay was run in triplicates. As controls, cathepsin inhibitors were used at indicated concentrations, namely: zFA-fmk, ALLN and CA-074-ME.

Proteasomal activity and flux

Proteasomal activity, hereunder caspase-like, trypsin-like, and chymotrypsin-like activity, was measured in cortical neurons with Proteasome-Glo^{tm} (Promega cat. no. 1180) according to manufactures description. CN were left untreated or pre-treated with rIFN-β (100 U/ml) or epoxomicin (10 nM) for 24 hours before adding Proteasome-Glo^{tm}. The plate was read in Glomax multi detection system (Promega) set for luminescence detection. Flux and degradation of the proteasomal substrate p53, was assessed by treating CN with cycloheximide (100 μg/ml) for 15 to 120 minutes, and the p53 levels were subsequently measured by WB.

Expression Affymetrix microarrays

RNA was extracted with TRI reagent (Sigma) and DNAse I (Invitrogen) from 3-day-old *Ifnb*$^{+/+}$ and *Ifnb*$^{-/-}$ CGN cultures in triplicates with or without rIFN-β (100 U/ml) treatment for the last 24 hours. We assessed RNA quality and concentration with an Agilent 2100 Bioanalyzer and Nanodrop ND-1000. Data from Affymetrix 430 2.0 microarray chips (SCIBLU, Affymetrix) was analyzed with Arraystar 3 software (DNA STAR Inc.), which were quantile-normalized and processed by the RMA (Affymetrix) algorithm. We $\log_2$-transformed the intensity values and normal-distributed data were tested in unpaired, two-tailed Student's *t*-tests, assuming equal variance and filtering for differential regulation confidence of 95% ($P<0.05$). Venn diagrams were created with oneChannelGUI (Bioconductor) from normalized RMA-analyzed data. Hybridization quality graphs are available on request. Quantile-normalized RMA-treated data selected using a 1.4-fold cut-off were analyzed with Ingenuity Pathway Analysis software. All raw microarray data are deposited at the Gene Expression Omnibus (GEO) database under the accession number GSE63815. Venn diagrams from normalized data were created with oneChannelGUI (www.bioconductor.org).

Heatmaps of the GSEA data were generated by extracting lists of core enriched genes from Autophagy (hereunder Lysosome and mTOR), Parkinson's, Huntington's, Alzheimer's, or Prion disease pathways of the GSEA analysis in R (Data File S1). Fold change gene expression values were calculated for the lists and the resulting tables were fed into Heatmap2 in R to construct the heatmaps.

Comparison between gene profile of *Ifnb*$^{-/-}$ CGNs and published models for PD, and HD disease affymetrix data (GSE4758, GSE9038) were quantile normalized together and summarized in R using the RMA algorithms from the affy-package. Identification of differential expression was performed individually within each experiment for our data as well as the published PD and HD models by comparing control/wild-type samples with transgenic samples using a standard ANOVA. In total, three sets of differentially expressed genes were generated and depicted in heatmaps: 1) Genes differentially expressed in our data ($p<0.001$); 2) Genes differentially expressed in both our data and the HD set ($p<0.01$); 3) Genes differentially expressed in both our and the PD set ($p<0.01$). The corresponding heatmaps were created using the pheatmap function in R. The settings were always the

same; Hierarchical clustering with Wards method on Euclidean distances where clustering was performed.

Statistical analysis

Data were analyzed with unpaired and paired two-tailed Student's *t*-tests, ANOVA, Mann-Whitney U test and Kruskal-Wallis test, with *P<0.05; **P<0.01; ***P<0.001 as significant values. Error bars are SEM.

Table S1 | Lack of *Ifnb* Gene is Associated with Increasing Penetrance in Behavioral Impairments. Related to Figure 1.

Age (months)	No. of mice	MWM*	No. of mice	RotaRod	Hanging test	Heat-induced pain	Cold-induced pain
1.5	8–15	11%	9–10	0%	0%	0%	0%
3	8–17	44%	8–11	62.5%	70%	87.5%	80%
6	8–16	49%	9–13	70%	92.3%	90%	78%
12	10–21	69%	10–13	69.2%	ND	ND	ND

Penetrance; i.e. the percentage of affected mice were calculated as the behavioral test scores of *Ifnb*$^{-/-}$ mice deviating from the norm; i.e., mean value – (SD/2) of the *Ifnb*$^{+/+}$ group. Two-way ANOVA were used to assess the age-dependent cognitive impairment in *Ifnb*$^{-/-}$ mice (*) (11%, 44%, 49%, 60%, indicate percentage of mice that did not improve their performance between probe 1 and 2 in each age group). MWM = Morris Water Maze, ND = not done.

Table S2 | Canonical KEGG Pathways from GSEA Analysis of *Ifnb*$^{-/-}$ Neurons (+/–) Recombinant IFN-β. Related to Figure 4.

	Gene set	Size	ES	NES	NOM p-val	FDR q-val	FWER p-val	Rank at max
1	KEGG FC GAMMA R MEDIATED PHAGOCYTOSIS	85	0,41	1,52	0,0000	0,2660	0,3153	2162
2	KEGG NEUROTROPHIN SIGNALING PATHWAY	115	0,30	1,49	0,0000	0,1847	0,3875	1397
**3**	**KEGG MTOR SIGNALING PATHWAY**	**44**	**0,34**	**1,38**	**0,0000**	**0,3694**	**0,6319**	**2121**
4	KEGG ENDOCYTOSIS	146	0,27	1,28	0,0000	0,6215	0,6611	4958
5	KEGG RENAL CELL CARCINOMA	67	0,25	1,22	0,1465	1,0000	1,0000	5308
6	KEGG B CELL RECEPTOR SIGNALING PATHWAY	70	0,32	1,22	0,1910	0,6208	1,0000	5723
7	KEGG TYPE II DIABETES MELLITUS	43	0,32	1,21	0,0708	0,5576	1,0000	1951
**8**	**KEGG LYSOSOME**	**109**	**0,24**	**1,20**	**0,0785**	**0,5243**	**1,0000**	**4992**
9	KEGG TYPE I DIABETES MELLITUS	20	0,42	1,17	0,1340	0,5688	1,0000	1075
10	KEGG AXON GUIDANCE	125	0,23	1,16	0,0970	0,5403	1,0000	5879
11	KEGG ALDOSTERONE REGULATED SODIUM REABSORPTION	36	0,39	1,11	0,1681	0,6792	1,0000	2074
12	KEGG ADIPOCYTOKINE SIGNALING PATHWAY	64	0,23	1,09	0,1444	0,6854	1,0000	1774
13	KEGG GLYCOSYLPHOSPHATIDYLINOSITOL GPI ANCHOR	22	0,28	1,07	0,1597	1,0000	1,0000	1695
14	KEGG ALANINE ASPARTATE AND GLUTAMATE METABOLISM	30	0,30	1,06	0,2208	0,6861	1,0000	1330
15	KEGG LEUKOCYTE TRANSENDOTHELIAL MIGRATION	102	0,28	1,05	0,2028	0,6681	1,0000	2057
16	KEGG FC EPSILON RI SIGNALING PATHWAY	76	0,28	1,02	0,2854	0,6840	1,0000	1767
17	KEGG LEISHMANIA INFECTION	49	0,30	1,01	0,1431	0,6653	1,0000	2057
18	KEGG NUCLEOTIDE EXCISION REPAIR	43	0,21	1,01	0,3563	0,6319	1,0000	2579
**19**	**KEGG REGULATION OF AUTOPHAGY**	**24**	**0,32**	**1,00**	**0,2618**	**0,6215**	**1,0000**	**3614**
20	KEGG RNA DEGRADATION	50	0,18	1,00	0,1958	0,6014	1,0000	2789

GSEA analysis was made on Affymetrix data that was quantile normalized and summarized for each comparison using RMA algorithm. ES = enrichment score; NES = normalized enrichment score; NOM p-val = nominal p-value; FDR q-val = False Discovery Rate; FWER p-val = family-wise error rate; RANK AT MAX = position in the ranked list at which the maximum enrichment score occurred.

Supplemental References

Brewer, G.J., and Torricelli, J.R. (2007). Isolation and culture of adult neurons and neurospheres. Nat Protoc *2*, 1490-1498.

Erlandsson, L., Blumenthal, R., Eloranta, M.L., Engel, H., Alm, G., Weiss, S., and Leanderson, T. (1998). Interferon-beta is required for interferon-alpha production in mouse fibroblasts. Curr Biol *8*, 223-226.

Kutner, R.H., Zhang, X.Y., and Reiser, J. (2009). Production, concentration and titration of pseudotyped HIV-1-based lentiviral vectors. Nat Protoc *4*, 495-505.

Liu, Y., Carlsson, R., Ambjorn, M., Hasan, M., Badn, W., Darabi, A., Siesjo, P., and Issazadeh-Navikas, S. (2013). PD-L1 expression by neurons nearby tumors indicates better prognosis in glioblastoma patients. J Neurosci *33*, 14231-14245.

Liu, Y., Carlsson, R., Comabella, M., Wang, J., Kosicki, M., Carrion, B., Hasan, M., Wu, X., Montalban, X., Dziegiel, M.H.*, et al.* (2014). FoxA1 directs the lineage and immunosuppressive properties of a novel regulatory T cell population in EAE and MS. Nat Med *20*, 272-282.

Liu, Y., Teige, I., Birnir, B., and Issazadeh-Navikas, S. (2006). Neuron-mediated generation of regulatory T cells from encephalitogenic T cells suppresses EAE. Nat Med *12*, 518-525.

Prinz, M., Schmidt, H., Mildner, A., Knobeloch, K.P., Hanisch, U.K., Raasch, J., Merkler, D., Detje, C., Gutcher, I., Mages, J.*, et al.* (2008). Distinct and nonredundant in vivo functions of IFNAR on myeloid cells limit autoimmunity in the central nervous system. Immunity *28*, 675-686.

Vorhees, C.V., and Williams, M.T. (2006). Morris water maze: procedures for assessing spatial and related forms of learning and memory. Nat Protoc *1*, 848-858.
