# Supplementary material for: Lack of Neuronal IFN-β-IFNAR Causes Lewy Body- and Parkinson’s Disease-like Dementia
Source: Cell. 2015 Oct 8;163(2):324–39. doi: 10.1016/j.cell.2015.08.069 (PMC4601085; doi:10.1016/j.cell.2015.08.069)
Supplement: Document S2. Article plus Supplemental Information [file mmc3.pdf]

Cell

Article

Lack of Neuronal IFN-β-IFNAR Causes Lewy Body- and Parkinson's Disease-like Dementia

Graphical Abstract

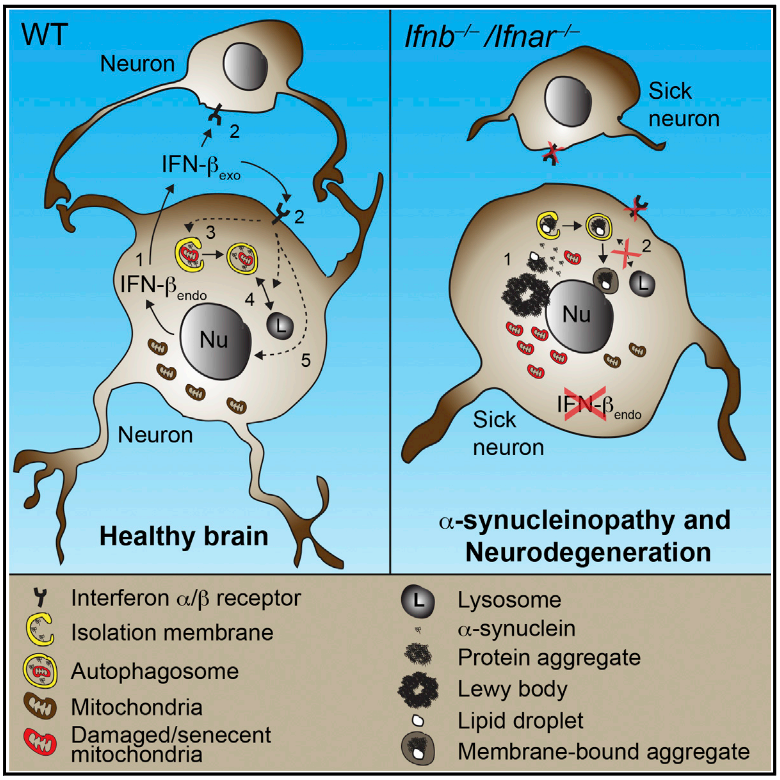

Authors

Patrick Ejlerskov, Jeanette Göransdotter Hultberg, JunYang Wang, ..., Marco Prinz, David C. Rubinsztein, Shohreh Issazadeh-Navikas

Correspondence

shohreh.issazadeh@bric.ku.dk

In Brief

Lack of the immunomodulatory cytokine interferon-β (IFN-β) causes spontaneous neurodegeneration resembling sporadic Lewy body and Parkinson's disease dementia due to defects in neuronal autophagy.

Highlights

- Lack of neuronal IFN-β-IFNAR signaling causes brain Lewy body accumulation
- IFN-β deficiency causes late-stage autophagy block and thereby α-synuclein aggregation
- IFN-β promotes neuronal autophagy and α-synuclein clearance
- *Ifnb* gene therapy prevents dopaminergic neuron loss in a familial PD model

Accession Numbers

GSE63815

Ejlerskov et al., 2015, Cell *163*, 324–339
October 8, 2015 ©2015 Elsevier Inc.
http://dx.doi.org/10.1016/j.cell.2015.08.069

Cell

Article

Lack of Neuronal IFN-β-IFNAR Causes Lewy Body- and Parkinson's Disease-like Dementia

Patrick Ejlerskov,[1] Jeanette Göransdotter Hultberg,[1] JunYang Wang,[1] Robert Carlsson,[1] Malene Ambjørn,[1] Martin Kuss,[1] Yawei Liu,[1] Giovanna Porcu,[1] Kateryna Kolkova,[1] Carsten Friis Rundsten,[1] Karsten Ruscher,[2] Bente Pakkenberg,[3] Tobias Goldmann,[4] Desiree Loreth,[5] Marco Prinz,[4,6] David C. Rubinsztein,[7] and Shohreh Issazadeh-Navikas[1,*]

[1]Biotech Research and Innovation Centre, University of Copenhagen, 2200 Copenhagen, Denmark
[2]Department of Clinical Sciences, Lund University, 22100 Lund, Sweden
[3]Research Laboratory for Stereology and Neuroscience, Bispebjerg University Hospital, 2200 Copenhagen, Denmark
[4]Institute for Neuropathology
[5]Department of Neurology
[6]Centre for Biological Signaling Studies
University of Freiburg, 79106 Freiburg, Germany
[7]Department of Medical Genetics, Cambridge Institute for Medical Research, Cambridge CB2 0XY, UK
*Correspondence: shohreh.issazadeh@bric.ku.dk
http://dx.doi.org/10.1016/j.cell.2015.08.069

SUMMARY

Neurodegenerative diseases have been linked to inflammation, but whether altered immunomodulation plays a causative role in neurodegeneration is not clear. We show that lack of cytokine interferon-β (IFN-β) signaling causes spontaneous neurodegeneration in the absence of neurodegenerative disease-causing mutant proteins. Mice lacking *Ifnb* function exhibited motor and cognitive learning impairments with accompanying α-synuclein-containing Lewy bodies in the brain, as well as a reduction in dopaminergic neurons and defective dopamine signaling in the nigrostriatal region. Lack of IFN-β signaling caused defects in neuronal autophagy prior to α-synucleinopathy, which was associated with accumulation of senescent mitochondria. Recombinant IFN-β promoted neurite growth and branching, autophagy flux, and α-synuclein degradation in neurons. In addition, lentiviral IFN-β overexpression prevented dopaminergic neuron loss in a familial Parkinson's disease model. These results indicate a protective role for IFN-β in neuronal homeostasis and validate *Ifnb* mutant mice as a model for sporadic Lewy body and Parkinson's disease dementia.

INTRODUCTION

Neurodegenerative diseases have disrupted neuronal homeostasis and their pathologies often overlap. Protein aggregates containing α-synuclein (α-syn), which eventually forms larger Lewy bodies (LBs), are seen in Parkinson's disease (PD), dementia with Lewy bodies (DLB), multiple system atrophy, and in some forms of Alzheimer's disease (AD), all neurodegenerative diseases associated with aging (Arima et al., 1999; Francis, 2009; Lippa et al., 1998). Often, the protein aggregates contain hyperphosphorylated tau and ubiquitin (Jellinger and Attems, 2008).

Neurodegenerative events in these diseases are linked to inflammation (Mrak and Griffin, 2007; Tansey et al., 2008), but, despite this link, defects in genes regulating inflammation do not have an established causative role in neurodegeneration. We report that deletion of *Ifnb*, which encodes interferon-β (IFN-β), is sufficient to cause a cascade of neurodegenerative events. IFN-β belongs to the type I interferon family of cytokines and binding to its receptor, interferon-α/β receptor (IFNAR), results in immunoregulation including anti-viral and anti-inflammatory effects (Prinz et al., 2008; Teige et al., 2003, 2006) and benefits for multiple sclerosis patients (Liu et al., 2014; Yong et al., 1998). However, the role of IFN-β in classic neurodegenerative diseases is unknown. We report that *Ifnb*$^{-/-}$ mice developed spontaneous pathologies mimicking major aspects of human neurodegeneration such as PD and DLB. *Ifnb*$^{-/-}$ mice had age-associated motor learning defects, neuromuscular deficiencies, and cognitive impairment. *Ifnb*$^{-/-}$ pathology was associated with LBs resulting from defective neuronal autophagy. Autophagy, a pathway that degrades long-lived proteins, organelles, lipids, and protein aggregates, is essential for neuronal homeostasis (Harris and Rubinsztein, 2012), and deleting neural autophagy-regulating genes leads to neurodegeneration (Hara et al., 2006; Komatsu et al., 2006).

Our findings indicate a central role for IFN-β in neuronal homeostasis as a regulator of autophagy-mediated protein degradation and accentuates *Ifnb*$^{-/-}$ mice as a model for neurodegenerative diseases with α-synucleinopathy and dementia such as PD and DLB.

RESULTS

Ifnb$^{-/-}$ Mice Exhibit Behavioral and Cognitive Impairments and Neurodegeneration

We examined the effect of *Ifnb* gene deletion on motor coordination, learning, and grip strength. *Ifnb*$^{-/-}$ mice were significantly impaired in motor coordination and learning from 3 months

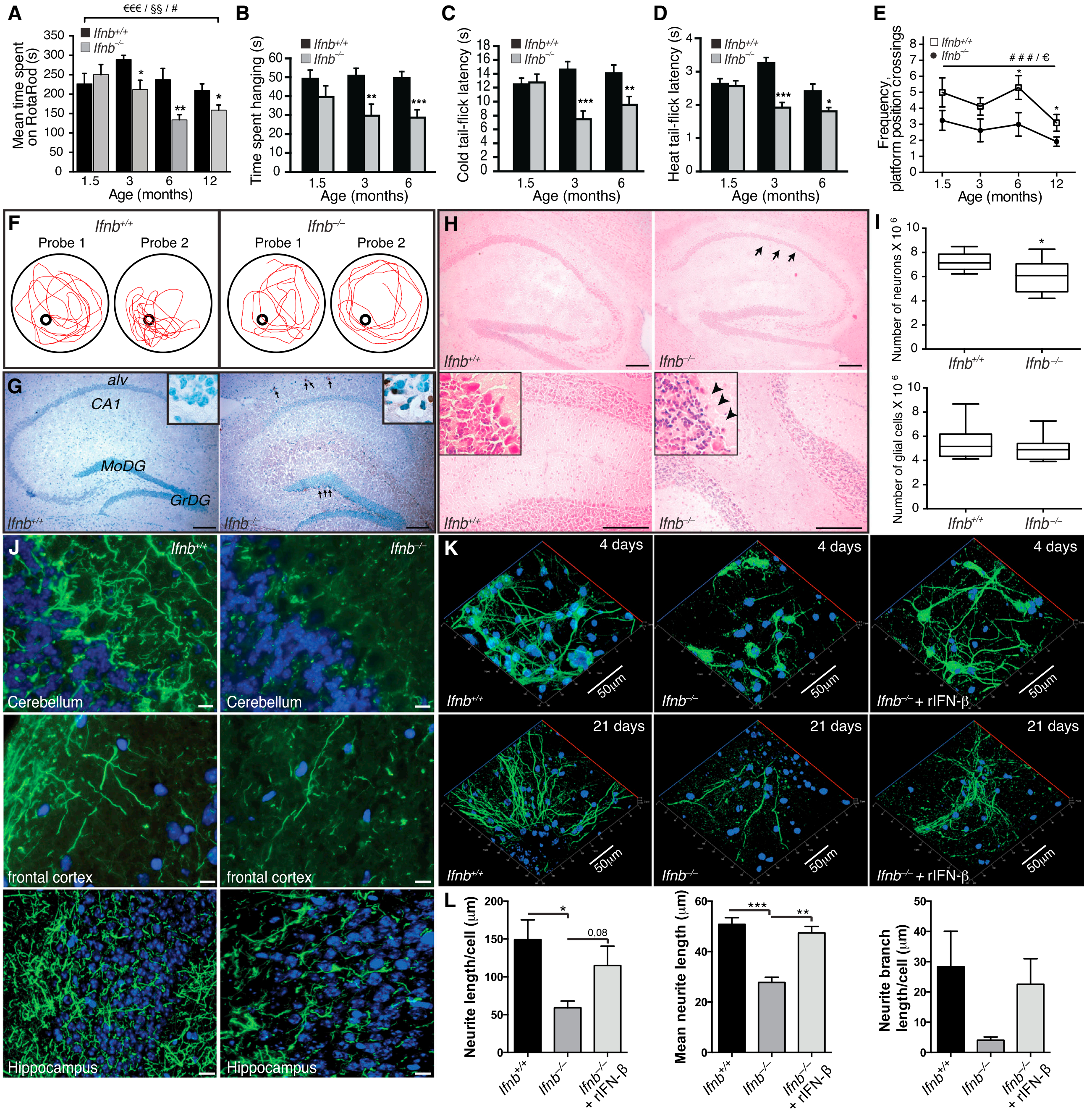

Figure 1. *Ifnb*$^{-/-}$ Mice Exhibit Spontaneous Behavioral and Cognitive Impairments and Neurodegeneration

(A) Motor coordination by RotaRod. Data are mean second(s) ± SEM, n = 8–13/group. *p < 0.05, **p < 0.01 for genotype effect per time point by unpaired Student's t test; $^{\#}$p < 0.05 for aging effect. $^{€€€}$p < 0.001 and §§p < 0.01 for genotype and aging effect, respectively, over time, by two-way ANOVA.

(B) Wire suspension performance. Data are mean ± SEM, 9–13/group.

(C and D) (C) Cold and (D) heat tail-flick latency. Data are mean ± SEM, 9–11/group.

(B–D) Unpaired Student's t test. *p < 0.05, **p < 0.01, ***p < 0.001.

(E) Morris water maze test. Data are mean ± SEM, 8–21 mice/group. $^{\#\#\#}$p < 0.001 shows genotype effect; $^{€}$p < 0.05 shows age effect; *p < 0.05 comparing genotypes by two-way ANOVA and (*) with Bonferroni post hoc test.

(F) Representative maze swimming pattern on probes 1 and 2.

(G) TUNEL staining of hippocampus from 1.5-month-old mice with methyl green nuclear counterstaining. MoDG, molecular dentate gyrus; GrDG, granular dentate gyrus; alv, alveus. Scale bar, 200 µm.

(H) H&E brain staining showing loss of granular cell layer and Purkinje cells (arrows) in *Ifnb*$^{-/-}$ hippocampus (upper) and cerebellum (lower). Scale bar, 200 µm.

(legend continued on next page)

compared to age-, sex-, and weight-matched wild-type (WT) *Ifnb*$^{+/+}$ littermates and in latency-to-fall time in a wire-suspension test (Figures 1A and 1B). We saw no differences in 1.5-month-old mice, suggesting that impaired motor coordination, balance, and grip strength were associated with age. During repeated motor-learning trials, retention time improved significantly in older *Ifnb*$^{+/+}$ mice compared to *Ifnb*$^{-/-}$ mice from age 3 months (Figure S1A), showing that reduced motor-learning in *Ifnb*$^{-/-}$ mice was associated with aging.

We assessed somatosensory function with nociception cold- and heat-induced tail-flick tests. Latency to tail flick was significantly shorter in *Ifnb*$^{-/-}$ than *Ifnb*$^{+/+}$ mice (Figures 1C and 1D), indicating hyperalgesia and defective nociception toward temperature-induced pain.

Forced swimming tests found no differences between *Ifnb*$^{-/-}$ and *Ifnb*$^{+/+}$ mice in swimming pattern, climbing effort, or immobility (Figure S1), so *Ifnb*$^{-/-}$ mice were not defective in locomotor activity in water in contrast to land. In water maze tests, *Ifnb*$^{-/-}$ mice had significant spatial- and memory-learning deficits that increased with age. During second tests, 6- and 12-month-old, *Ifnb*$^{-/-}$ mice had significantly fewer platform position crossings compared to *Ifnb*$^{+/+}$ mice, indicating impaired reference memory (Figures 1E and 1F). *Ifnb*$^{+/+}$ mice improved between the first and second tests at all ages except 12 months; *Ifnb*$^{-/-}$ mice did not improve, indicating impaired spatial learning. This was seen at all ages in *Ifnb*$^{-/-}$ mice measured by escape latency time in the second learning block (Figures S1B and S1C). Thus, although *Ifnb*$^{-/-}$ mice began with no major behavioral and cognitive defects, they developed age-dependent deficits with increased penetrance (Table S1).

IFN-β Is Essential for Neuronal Survival, Neurite Outgrowth, and Branching

We investigated whether *Ifnb*$^{-/-}$ behavioral deficits were associated with neurodegeneration. Apoptotic cells were detected in 1.5-month-old *Ifnb*$^{-/-}$ granular layers of olfactory bulbs (Figure S1E), the granular dentate gyrus of hippocampus and the subventricular zone (Figure 1G), and the striatum (STR) caudate putamen including the ependymal cell layer in 12-month-old *Ifnb*$^{-/-}$ mice (Figure S1F), but not detected in *Ifnb*$^{+/+}$ sagittal brain sections at similar ages. Apoptotic neurons increased in cultured *Ifnb*$^{-/-}$ primary cerebellar granular neurons (CGNs) and was reversed by recombinant (r)IFN-β (Figures S1G and S1H). Neurons were significantly reduced in the hippocampal CA1 region in 3- to 6-month-old *Ifnb*$^{-/-}$ mice and decreased in Purkinje cells of cerebellum. Glial cell counts were unchanged (Figures 1H and 1I).

Supporting in vivo deficits in neuronal circuits, *Ifnb*$^{-/-}$ mice had reduced neurite network formation in the cerebellum, frontal cortex, and hippocampus granular cell layer (Figure 1J). This was confirmed in cultured *Ifnb*$^{-/-}$ primary cortical neurons (CNs) that had reduced neurite length per cell, mean length, and branch length per cell after 4 days, with restoration by rIFN-β (Figures 1K and 1L). A similar trend was seen after 21 days of culture (Figure 1K). Cultured primary *Ifnb*$^{-/-}$ CGNs mimicked the findings of CNs (Figures S1I and S1J). Thus, behavioral defects in old *Ifnb*$^{-/-}$ mice were associated with neuron death and reduced neurite circuits.

Ifnb$^{-/-}$ Neuron Gene Profiling Reveals Neurodegenerative Paths

We identified neuron-specific signaling and disease pathways caused by IFN-β deficiency with expression microarrays on highly pure (>98%) primary CGNs from *Ifnb*$^{-/-}$ and *Ifnb*$^{+/+}$ mice plus or minus rIFN-β. *Ifnb*$^{-/-}$ neurons had 323 upregulated and 233 downregulated genes (Figures 2A, 2B, and S2A). rIFN-β treatment had differential effects on *Ifnb*$^{+/+}$ versus *Ifnb*$^{-/-}$ CGNs with approximately equal numbers of downregulated and upregulated genes, but with relatively few overlaps (Figures 2C–2F). Multiple genes in the *Ifnb*$^{-/-}$ data set annotated as neuronal degeneration and the top ten pathways included cell death and neurological disorders. Genes in neurite formation and branching suggested compromised neurogenesis pathways in *Ifnb*$^{-/-}$ neurons, supporting the in vitro and in vivo phenotypes (Figures 2G and 2H). To investigate whether *Ifnb* and signaling via IFNAR affected neurogenesis in vivo, 3-month-old *Ifnb*$^{-/-}$ and IFNAR knockout (KO) mice (*Ifnar*$^{-/-}$) and WT littermates were injected with bromodeoxyuridine (BrdU). Lack of *Ifnb* or receptor was associated with reduced neurogenesis in the hippocampus; dentate gyrus (Figure 2I), supporting the microarray data. IFN-β might directly affect prosurvival mechanisms as neurotrophin was among the top hits in gene set enrichment analysis (GSEA) (Table S2) and mRNA for the corticogenesis-regulating transcription factor Tox (Artegiani et al., 2015) was reduced in *Ifnb*$^{-/-}$ neurons (Figure S2A). Increased apoptosis and reduced neurogenesis might contribute to impaired cognition and reduced hippocampal neuron numbers. GSEA of *Ifnb*$^{+/+}$ and *Ifnb*$^{-/-}$ showed enrichment of genes associated with Huntington's disease (HD), PD, AD, and prion diseases, and rIFN-β caused the *Ifnb*$^{-/-}$ profile to resemble WT (Figure 2J), supporting that common pathways link neurodegenerative diseases (Shulman and De Jager, 2009).

We compared the *Ifnb*$^{-/-}$ neuronal gene profile with PD and HD mouse models (Fossale et al., 2011). Three sets of differentially expressed genes were generated: differentially expressed in our data ($p < 0.001$), in our data and the HD model ($p < 0.01$), or our data and the PD model ($p < 0.01$) (Figures S2A–S2C; Data S1). Clustered genes in *Ifnb*$^{-/-}$ neurons and the HD model were both similarly and oppositely regulated. In contrast, most clustered genes in *Ifnb*$^{-/-}$ neurons and the PD model were regulated similarly, thus indicating a higher gene signature resemblance with PD than HD (Figures S2B and S2C).

(I) Number of neurons and glial cells in hippocampus of 3- to 6-month-old mice. Data are mean ± SEM, n = 6 mice/group. *$p < 0.05$ using Student's t test.
(J) IF of 3-month-old brain sections. Scale bars, 10 μm.
(K) Z stack projections of CNs cultured for 4 and 21 days with or without rIFN-β. Scale bar, 50 μm.
(L) Quantified neurite length from 4-day-old CNs.
Data are mean ± SEM, n = 3–4, counting 11–32 cells per experiment. *$p < 0.05$, **$p < 0.01$, ***$p < 0.001$ by one-way ANOVA. See also Figure S1 and Table S1.

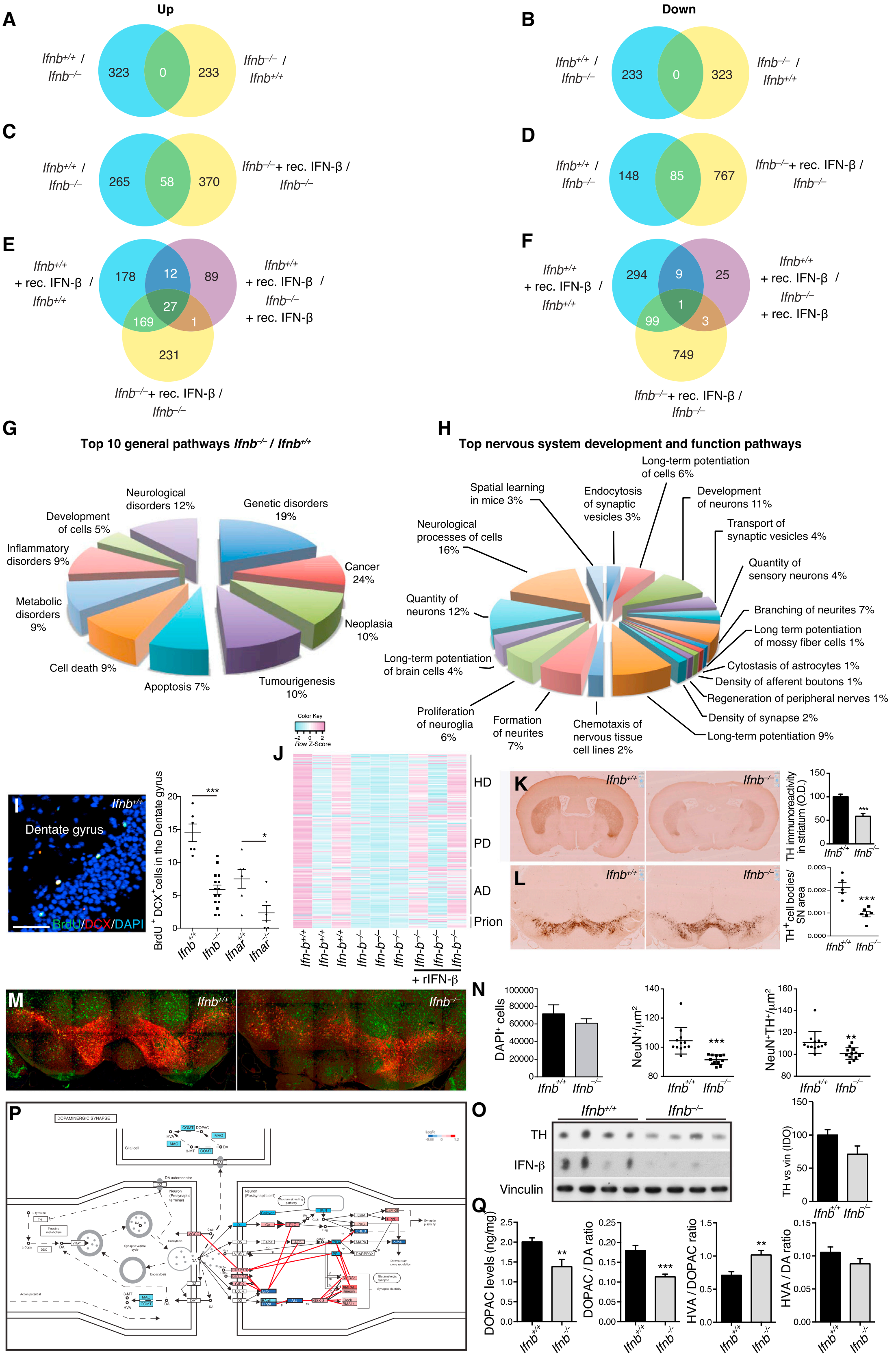

(legend on next page)

Lack of *Ifnb* Causes Defects in the Nigrostriatal Dopaminergic Pathway

Based on the possible PD-related defect in *Ifnb*$^{-/-}$ neurons, we investigated nigrostriatal region integrity. Tyrosine hydroxylase (TH)$^{+}$ fiber density and TH^{+} (dopamine-producing) neurons were significantly reduced in the STR and substantia nigra (SN) in *Ifnb*$^{-/-}$ mice versus WT (Figures 2K and 2L). NeuN^{+} and NeuN^{+}TH^{+} cells were reduced in the ventral midbrain, which was correlated with reduced TH protein in basal ganglia (BG) in *Ifnb*$^{-/-}$ mice while total cells were unaffected (Figures 2M–2O). Among differentially expressed genes in *Ifnb*$^{-/-}$ neurons, 31 (p = 6.937e-4) were involved in regulating dopamine (DA) signaling (Figure 2P), which is involved in coordinating movements. DA signaling was also defective in the STR. No major differences were found in levels of DA or its metabolite homovanillic acid (HVA) (data not shown) in 3- to 6-month-old *Ifnb*$^{-/-}$ mice, but the DA metabolite dihydroxyphenylacetic acid (DOPAC) was significantly lower, thus significantly affecting DA/DOPAC and DOPAC/HVA ratios (Figure 2Q). These findings support the importance of IFN-β in regulating dopamine turnover and protecting dopaminergic neurons.

Lack of Neuronal IFN-β-IFNAR Signaling Causes Lewy Bodies

Since *Ifnb*$^{-/-}$ mice defects overlapped with neurodegenerative diseases, particularly PD, we examined *Ifnb*$^{-/-}$ brain pathology. Gross anatomy was unchanged, but histological examination showed that *Ifnb*$^{-/-}$ neuron degeneration was associated with age-dependent α-synucleinopathy. Staining for α-syn was normal in 1.5-month-old *Ifnb*$^{-/-}$ brains; by 3 months, α-syn was found in LB-like structures in SN; however, α-syn staining intensity was reduced in 12-month-old *Ifnb*$^{-/-}$ mice (Figures S3A and S3B), likely reflecting degeneration of TH^{+} neuron (Figure 2L). Alpha-syn and large pathogenic aggregates of phosphorylated (pSer129) α-syn were found in TH^{+} DA neurons of SN (Figure 3A). At 3 months, α-syn aggregates were widespread in the STR, frontal cortex (Figure 3B), hippocampus, and cerebellum (Figures S3C and S3D). Alpha-syn^{+} aggregates and neurites were found sporadically in thalamus, the brainstem, and subthalamic regions of 3-month-old *Ifnb*$^{-/-}$ mice (Figures S3E–S3G). Neurons with α-syn^{+} LB-like structures increased with age (6- and 12-month-old) in *Ifnb*$^{-/-}$ mouse thalamus (Figures 3C and 3D). Whole-brain protein extracts from 1.5-month-old mice were not different from WT, but 3-month-old mice had significantly increased α-syn in insoluble fractions and no difference in soluble fractions (Figures 3E–3I). No difference in mRNA for α-syn was observed between *Ifnb*$^{-/-}$ and WT brains (Figure 3J); thus, the α-syn accumulation was not due to increased transcription. The insoluble α-syn fraction in the BG (including SN) was significantly higher in older mice, but the soluble fraction was still unchanged. A significant increase in high-molecular-weight dimeric, trimeric, and oligomeric α-syn were seen in *Ifnb*$^{-/-}$ mice, but with a decrease in tetramers. While aggregated α-syn oligomers are neurotoxic (Rockenstein et al., 2014), α-syn tetramers are suggested to be the normal aggregation resistant conformation of the protein (Bartels et al., 2011; Wang et al., 2011), possibly explaining the tetramer abundance in *Ifnb*$^{+/+}$ mice. pSer129-α-syn, which is prone to form pathogenic fibrillar aggregates, was increased in *Ifnb*$^{-/-}$ mice (Figures 3K–3M). *Ifnar*$^{-/-}$ brains showed a similar pattern to *Ifnb*$^{-/-}$ regarding α-syn and pSer129-α-syn accumulation (Figure S3H). Blocking with α-syn$_{96-140}$ peptide confirmed specific immunoreactivity for α-syn in *Ifnb*$^{-/-}$ brains. To ensure that lack of *Ifnb* did not generate crossreacting α-syn-independent aggregates, we generated *Scna*$^{-/-}$*Ifnb*$^{-/-}$ double-KO mice (DKO). *Ifnb*$^{-/-}$ but not *Scna*$^{-/-}$ or DKO mice had α-syn^{+} aggregates or LBs (Figures S3I–S3K).

Accumulated polyubiquitinated proteins are associated with neurodegenerative disease (Davies et al., 1997; Hara et al., 2006; Komatsu et al., 2006). While 1.5-month-old WT and *Ifnb*$^{-/-}$ mice showed not differences, 3-month-old *Ifnb*$^{-/-}$ brains had increased polyubiquitin (Figures 3N–3R), in hippocampus and cerebellum (Figures S3L and S3M), SN and locus coeruleus (Figures 3O and 3P) and vestibular nuclei of pons (not shown). Ubiquitin accumulated with inclusion body abundance, as seen in the locus coeruleus of 12-month-old *Ifnb*$^{-/-}$ mice (Figure 3Q) and α-syn^{+} aggregates colocalizing with ubiquitin and phosphorylated Tau (pTau) significantly increased (Figures 3S and 3T).

To exclude systemic immune response influences, we analyzed *Ifnar*$^{-/-}$ and *nes*^{Cre}:*Ifnar*$^{fl/fl}$ mice. Consistent with *Ifnb*$^{-/-}$ mice, α-syn^{+} LB-like structures were found in the

Figure 2. Gene Profiling of *Ifnb*$^{-/-}$ Neurons Shows Association with Neurodegenerative Diseases

Mouse Affymetrix 430 2.0 arrays.

(A–F) Venn diagrams for gene expression analysis of *Ifnb*$^{-/-}$, *Ifnb*$^{+/+}$ CGNs with or without rIFN-β, n = 3. Genes differentially regulated by 1.5-fold, *$p < 0.05$. (A, C, and E) Upregulated. (B, D, and F) downregulated genes.

(G and H) Gene expression analyzed for (G) top ten general signaling pathways and (H) nervous system development and function pathways.

(I) Neurogenesis in hippocampus dentate gyrus showing IF of an *Ifnb*$^{+/+}$ mouse and quantifying all groups: n = 4 mice/group and six to 12 sagittal sections were counted for BrdU^{+}DCX^{+} neurons.

(J) GSEA Heatmaps of core-enriched pathways; HD, PD, AD, and prion disease pathways were the top four neurologic disease pathways without or with rIFN-β, n = 3.

(K and L) IHC TH staining of coronal (K) STR and (L) SN sections. Data are (K) mean optical density (OD) ± SEM and (L) TH^{+} cell bodies per SN area ± SEM, n = 4 mice.

(M and N) Stitched IF images showing NeuN (green) and TH (red) (M) immunoreactivity and (N) quantifications of n = 4 mice/group in three to four ventral midbrain regions.

(O) WB of BG from 8-month-old mice (n = 4). Graph, mean integral optical density (IOD) ± SEM of TH bands.

(P) Overview of the DA system. Red, upregulated; blue, downregulated genes from comparative microarray analysis of *Ifnb*$^{+/+}$ and *Ifnb*$^{-/-}$ CGNs.

(Q) High-performance liquid chromatography (HPLC) analysis of DA, DOPAC, and HVA in STR of 3- to 6-month-old mice, n = 9–10/group.

For (I), (K), (L), (N), and (Q), *$p < 0.05$, **$p < 0.01$, ***$p < 0.001$, by Student's t test. See also Figure S2 and Data S1.

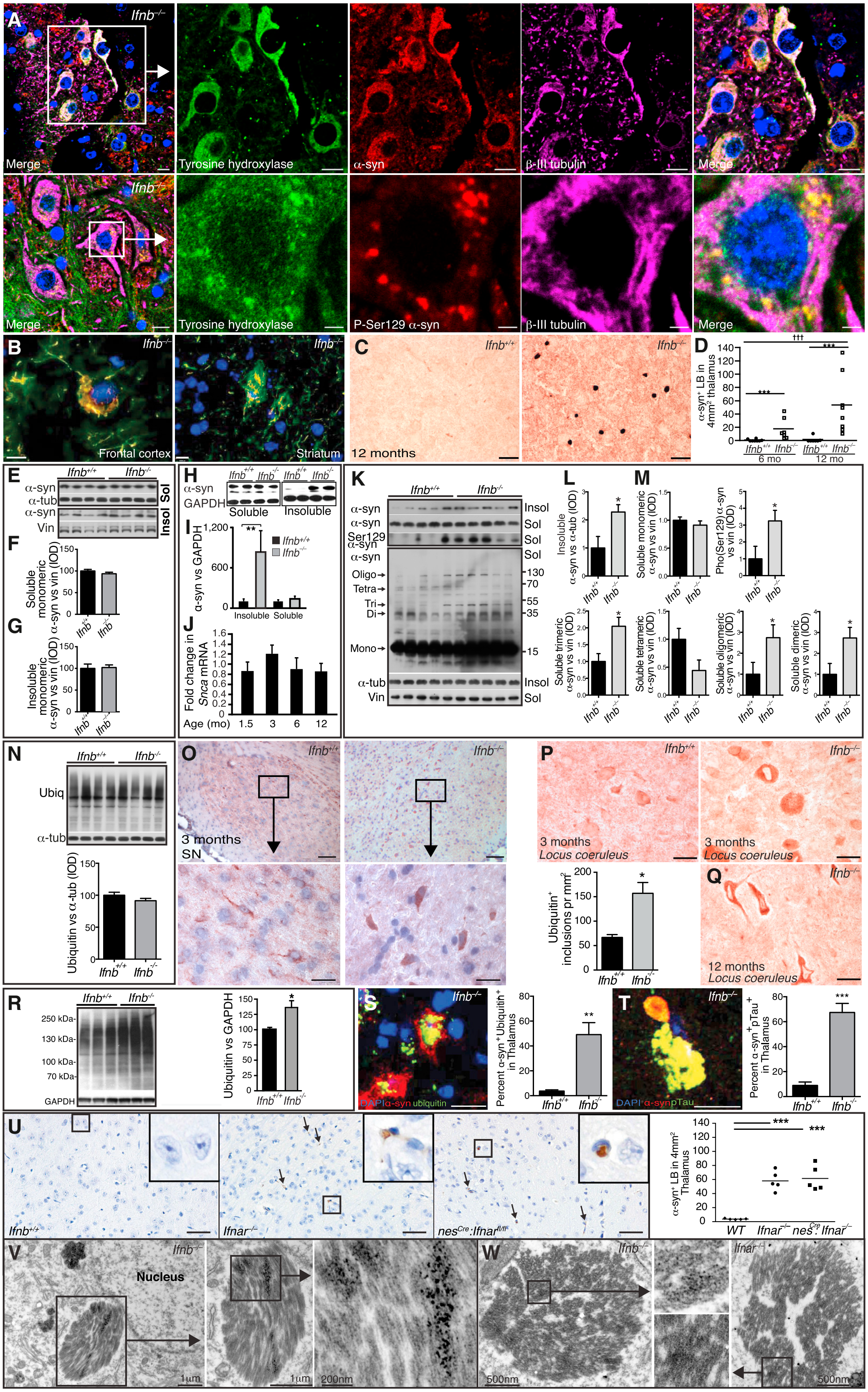

(legend on next page)

cerebellum, granular and molecular layers (Figure S3N), and significantly in thalamus of *Ifnar*$^{-/-}$ and *nes*^{Cre}:*Ifnar*$^{fl/fl}$ mice (Figure 3U). The ultrastructure of *Ifnb*$^{-/-}$ and *Ifnar*$^{-/-}$ neurons in paratenial and central medial thalamic nuclei contained α-syn-immunoreactive perinuclear LB-like structures (Figures 3V and 3W), which were absent in *Ifnb*$^{+/+}$ mice (data not shown), underscoring the importance of endogenous IFN-β–IFNAR signaling in preventing neuronal proteinopathy.

Lack of IFN-β Affects Autophagy

We used GSEA to identify cellular pathways involved in *Ifnb*$^{-/-}$ neuron pathology. In the top 20 deregulated pathways, three were associated with autophagy, which were restored with rIFN-β (Figure 4A; Table S2). The autophagy system uses adaptor proteins SQSTM1/p62 (hereafter p62) and NBR1 to bind ubiquitinated proteins and LC3B-II in the autophagosomal isolation membrane. Upon autophagy, cytosolic LC3B-I is converted to membrane bound LC3B-II and blocking autophagy flux, e.g., with Rab7 defects (Gutierrez et al., 2004; Hyttinen et al., 2013), causes accumulation of autophagy-targeted proteins; p62, NBR1, LC3B-II, and organelles. Increased neurons with accumulated p62 were seen in 3-month-old *Ifnb*$^{-/-}$ brains compared to WT, predominantly in brainstem, without increased p62 mRNA. LC3B-II increased in BG of 1.5-month-old *Ifnb*$^{-/-}$, correlating with increased p62, NBR1 and Rab7, supporting defects in autophagy before α-syn, ubiquitin, pTau, and LB aggregation. Autophagy was even more deregulated in 6- to 8-month *Ifnb*$^{-/-}$ brains (Figures 4B–4H). Defective autophagy was also confirmed in *Ifnar*$^{-/-}$ mice (Figure S4).

Senescent or damaged mitochondria are degraded by mitophagy. Large, cytoplasmic, electron-dense aggregates associated with lipid droplets were exclusively found in *Ifnb*$^{-/-}$ and *Ifnar*$^{-/-}$ thalamic neurons, which correlated with significantly more mitochondria than in WT (Figures 4I–4M). Cultured *Ifnb*$^{-/-}$ neurons had significantly lower mitochondrial membrane potential (MMP) than *Ifnb*$^{+/+}$ neurons, indicating senescent or damaged mitochondria. MMP was not affected by rIFN-β contrary to positive and negative regulators (Figure 4N). Thus, IFN-β signaling was important in regulating autophagy flux including neuronal mitophagy.

Late-Stage Autophagy Block Causes α-syn Accumulation

Ifnb$^{-/-}$ CNs and CGNs and *Ifnar*$^{-/-}$ CNs showed autophagy defects resembling a late-stage autophagy block (Figures 5A, 5B, S5A, and S5B). In *Ifnb*$^{-/-}$ CNs, LC3B-I and LC3B-II were increased with p62, NBR1, and K-63-linked ubiquitin, while K-48-linked ubiquitin only increased slightly (Figures 5A and 5B). Both *Ifnb*$^{+/+}$ and *Ifnar*$^{-/-}$ produced detectable IFN-β, unlike *Ifnb*$^{-/-}$ neurons (Figure S5C). To validate a block in autophagy flux, fusion between autophagosomes and lysosomes was inhibited with NH_4Cl. At 1 hr, LC3B-II levels saturated in both genotypes, which correlated with a significant increase in p62 in *Ifnb*$^{+/+}$ CNs; however, *Ifnb*$^{-/-}$ CNs had nearly saturated p62 levels without treatment, and no significant increase was seen with NH_4Cl (Figures 5C and 5D). Autophagy flux was measured in CNs with the mRFP-GFP-LC3B construct, which emits mRFP and GFP signal in autophagosomes and only mRFP in autolysosomes because of low pH in the latter. *Ifnb*$^{-/-}$ CNs had increased mCherry^{+}GFP^{+} autophagosomes and very few autolysosomes compared to WT again suggesting blocked autophagy flux. Treating *Ifnb*$^{+/+}$ CNs with NH_4Cl caused a similar distribution as *Ifnb*$^{-/-}$ CNs. Rab7 significantly increased in *Ifnb*$^{-/-}$ CNs, suggesting accumulation of mature autophagosomes. In support, fewer LC3B- and p62-positive autophagic vacuoles overlapped with LAMP1, a lysosomal marker, in *Ifnb*$^{-/-}$ neurons indicating flux or fusion problems (Figures 5E–5H). GSEA suggested dysregulation in lysosomal genes in *Ifnb*$^{-/-}$ neurons (Table S2). However, numbers and dysfunction of lysosomes measured as acidification, morphology, or cathepsin expression and activity did not differ in *Ifnb*$^{+/+}$ and *Ifnb*$^{-/-}$ CNs and brains (Figures S5D–S5I).

Figure 3. *Ifnb*$^{-/-}$ Mice Develop LBs with α-syn, Ubiquitin, and pTau

(A) IF of α-syn in SN of 6-month-old *Ifnb*$^{-/-}$ mice. Scale bar, 10 μm; 2 μm in close-up lower panel.

(B) IF of 3-month-old *Ifnb*$^{-/-}$ brains showing α-syn^{+} neuron in frontal cortex and positive aggregates in STR. Scale bars, 10 μm.

(C) IHC of α-syn in thalamus. Scale bar, 20 μm.

(D) Number of α-syn-positive LBs in thalamus of 6- and 12-month-old mice. ***$p < 0.001$ by Mann-Whitney U test for two groups, $^{†††}p < 0.001$ by Kruskal-Wallis test for all groups.

(E–G) (E) WB of α-syn and quantified IOD in (F) soluble and (G) insoluble BG fractions of 1.5-month-old mice. Data are mean ± SEM, n = 4.

(H and I) (H) WB of α-syn and (I) quantified IOD in TX-100 soluble and insoluble fractions of 3-month-old brains. Data are mean ± SEM, n = 5.

(J) RT-PCR of α-syn in *Ifnb*$^{-/-}$ relative to *Ifnb*$^{+/+}$ brains. Data are mean ± SEM, n = 3–5.

(K) WB of α-syn (TX-100 soluble and insoluble) from BG of 8-month-old mice and long exposure of whole membranes of soluble fractions.

(L and M) Quantified IOD of α-syn and pSer129-α-syn in (L) insoluble and (M) soluble fractions of monomeric (short exposure) and high-molecular-weight α-syn species (long exposure). Graphs, mean ± SEM, n = 5.

(N) WB and quantified ubiquitin IOD in BG from 1.5-month-old mice. Data are mean ± SEM, n = 3.

(O) IHC of ubiquitin in SN at 3 months and quantified ubiquitin^{+} aggregates/μm^2. Scale bar, 200 μm; 20 μm in inserts. Graph, mean ± SEM, n = 5.

(P and Q) IHC of ubiquitin in locus coeruleus at (P) 3 months and (Q) 12 months. Scale bar, 200 μm.

(R) WB and quantified ubiquitin IOD in brain extracts from 3-month-old mice. Data are mean ± SEM, n = 3.

(S and T) IF and quantification of (S) ubiquitin^{+}α-syn^{+} and (T) p-tau^{+}α-syn^{+} aggregates in thalami of 12-month-old mice. Scale bar, 10 μm; data are percentage double positives ± SEM.

(U) IHC of α-syn in mice thalami showing LB-like structures (arrows). Scale bar, 50 μm. Graph, α-syn^{+} LB-like structures in thalamus ± SEM, n = 5. ***$p < 0.001$ by one-way ANOVA and Turkey's post hoc correction test.

(V and W) Immuno-EM of thalamic neurons from *Ifnb*$^{-/-}$ and *Ifnar*$^{-/-}$ mice showing LB-like structures positive for α-syn by (V) immuno-DAB reactivity and (W) immunogold labeling.

For (I), (L), (M), (O), (R), (S), and (T), *$p < 0.05$, **$p < 0.01$, ***$p < 0.001$ by Student's t test. See also Figure S3.

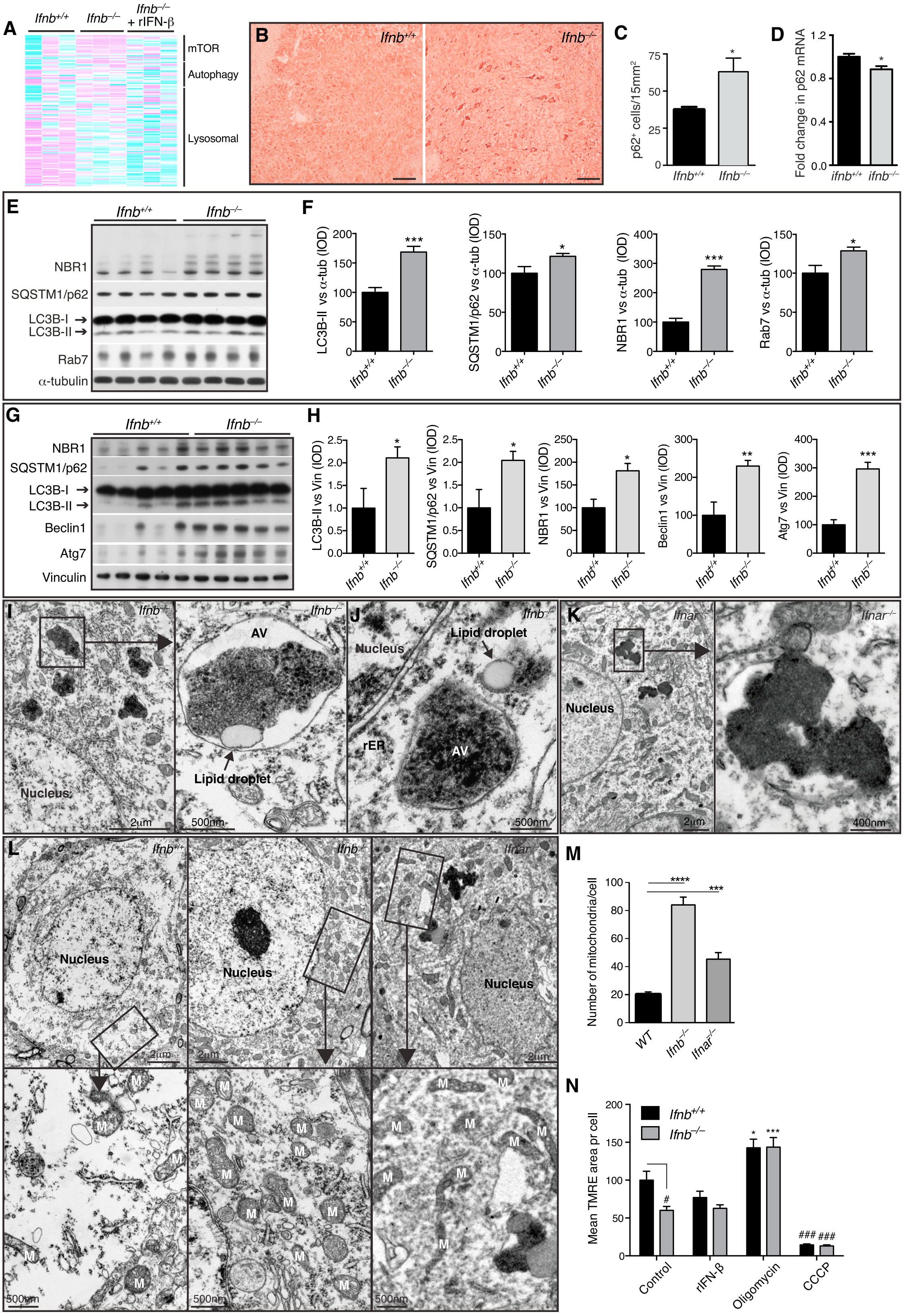

(legend on next page)

Ifnb$^{-/-}$ CNs developed ubiquitin^{+} α-syn aggregates and increased monomeric and high-molecular-weight α-syn after 21 days of culture (Figures 6A and 6B). Accumulation of α-syn was not due to proteasomal defects; turnover of the proteasome substrate p53 after cycloheximide treatment and proteasomal catalytic activity were uncompromised in *Ifnb*$^{-/-}$ CNs and unchanged by rIFN-β (Figure S6).

Blocking autophagy flux increased LC3B-II and α-syn, and α-syn colocalized with LC3B^{+} and p62^{+} autophagosomes in *Ifnb*$^{-/-}$ CNs (Figures 6C and 6D), underscoring the relevance for autophagic clearance of α-syn. Aggregation-prone pSer129-α-syn was seen in LC3B^{+} autophagosomes, that rarely overlapped with LAMP1. Collectively, these results demonstrated that lack of IFN-β reduced lysosomal fusion and caused α-syn accumulation.

IFN-β Promotes Neuronal Autophagy and α-syn Clearance

LC3B-II and p62 were higher in untreated *Ifnb*$^{-/-}$ CNs, and, while overnight rIFN-β-treatment promoted LC3B-II conversion and reduced p62 in *Ifnb*$^{+/+}$ CNs, indicating increased autophagy flux, rIFN-β reduced p62 but only slightly increased LC3B-II in *Ifnb*$^{-/-}$ CNs. By promoting autophagy, rIFN-β reduced α-syn in both *Ifnb*$^{+/+}$ and *Ifnb*$^{-/-}$ CNs (Figures 7A–7D). Effects of rapamycin, an mTOR-dependent autophagy activator, were similar to rIFN-β, but the mTOR-independent inducer trehalose more efficiently reduced α-syn in CNs from both genotypes, possibly through increased α-syn secretion (Ejlerskov et al., 2013). To ensure reconstitution of the genetic defect, we cultured *Ifnb*$^{-/-}$ neurons with a low rIFN-β dose for 21 days, which increased autophagy flux and α-syn clearance to the level in *Ifnb*$^{+/+}$ neurons (Figures 7E and 7F).

Ifnb Gene Therapy Prevents Dopaminergic Neuron Loss in a Familial PD Model

We used lentiviruses to overexpress IFN-β to examine effects on a familial PD model induced with human α-syn (hSCNA) in rat SNs. Injection of hSCNA and control lentiviruses blocked autophagy indicated by accumulated LC3B-II, p62, NBR1, Beclin1, and hSCNA in BG 10 days after SN injection (Decressac et al., 2013) (Figures 7G and 7H). *Ifnb* overexpression prevented hSCNA and pSer129-α-syn accumulation and restored TH loss. The mice showed improved left paw use compared to right paw use; contralateral to injection side of hSCNA/*Ifnb* and hSCNA/control viruses, respectively, 21 days post injection, which was associated with preservation of TH^{+} fibers in SN (Figures 7I and 7J). *Ifnb* gene therapy also significantly protected TH^{+} dopaminergic neurons from hSCNA-induced SN damage (Figure 7K). Thus, IFN-β prevented pathology in a familial model of PD by inducing autophagy and α-syn clearance.

DISCUSSION

CNS immune activation and inflammation occur in neurodegenerative diseases (Brochard et al., 2009; Lee et al., 2009; Maccioni et al., 2009; Tansey et al., 2008), but their role in initiation is unclear. We report that defects in IFN-β-IFNAR signaling, that is central to immune regulation, trigger neurodegeneration in the CNS of aging mice. IFN-β promotes neurite growth and protein degradation by autophagy. Lack of IFN-β causes neural pathological changes: accumulation of LB-like structures, neural apoptosis, and neurogenesis defects. p62 accumulated in *Ifnb*$^{-/-}$ brainstems and BG regions before pathological α-syn aggregation. Aggregates and LB-like structures with α-syn were seen in brainstem and BG including SN, cortex, thalamus, and cerebellum as previously reported in PD and DLB patients (Goedert et al., 2013; Mori et al., 2003).

IFN-β is expressed by neurons to prevent malignant growth (Liu et al., 2013) and neuroinflammation (Liu et al., 2014) and is produced by choroid plexus epithelial cells in aged mouse and human brains without CNS disease (Baruch et al., 2014). In the latter study, injection of an anti-IFNAR antibody in the cerebrospinal fluid, however, positively affected some aspects of cognition. This approach could have complications as injection of full-length antibody might initiate complement-dependent cytotoxicity (Nelson, 2010) and inflammation (Congdon et al., 2013; Linnartz et al., 2012) and cause rapid antibody clearance compared to isotype controls (Sheehan et al., 2006). Genetic generation of *Ifnb* or *Ifnar* knockout mice or specifically targeting neuroectodermal cells in *nes*^{Cre}*:Ifnar*$^{fl/fl}$ mice circumvents such issues. We found that neuronal IFN-β-IFNAR signaling is required for neurons to withstand age-associated pathology. The data also suggest that neuronal IFN-β production in the CNS parenchyma might function differently than high production by choroid plexus epithelial cells or potentially resident microglia.

Familial but not sporadic neurodegenerative diseases are associated with overexpressed or mutant proteins such as

Figure 4. Late-Stage Degradative Autophagosomes Accumulate in *Ifnb*$^{-/-}$ Neurons

(A) Heatmaps of GSEA core-enriched genes in autophagy-related pathways comparing *Ifnb*$^{+/+}$ and *Ifnb*$^{-/-}$ mouse CGNs, *Ifnb*$^{-/-}$ with or without 24 hr rIFN-β (100 U/ml), n = 3.

(B) IHC from pons area using p62 antibodies. Scale bar, 20 μm.

(C) Quantification of p62^{+} cells/15 mm^2. Data are mean ± SEM, n = 5.

(D) Fold change in p62 mRNA in brain extracts of 3-month-old mice. Data are mean ± SEM, n = 3.

(E–H) WB of BG of (E) 1.5-month-old and (G) 6- to 8-month-old mice and (F) and (H) quantified IOD of bands. Data are mean ± SEM of four or five brains.

(I–L) TEM of 9-month-old mice thalami. (I–J) *Ifnb*$^{-/-}$ mice showing perinuclear electron-dense late-stage autophagic vacuoles (AV), most surrounded by single lipid membrane and associated with lipid droplets. rER, rough endoplasmic reticulum. (K) *Ifnar*$^{-/-}$ mouse with similar electron-dense aggregates. (L) TEM of mitochondria in thalamic neuron cell body. M, mitochondria.

(M) Graph, mean ± SEM mitochondria per cell body of 12–16 thalamic neurons; ***$p < 0.001$, ****$p < 0.0001$ by one-way ANOVA with post hoc Dunnett's multiple comparisons test.

(N) TMRE analysis of 21-day-old CNs with mean TMRE area per cell, n = 3. *Treatment effect, and #genotype effect.

For (B), (C), (F), (H), and (N), */#$p < 0.05$, **$p < 0.01$, ***/###$p < 0.001$ by unpaired Student's t test. See also Figure S4 and Table S2.

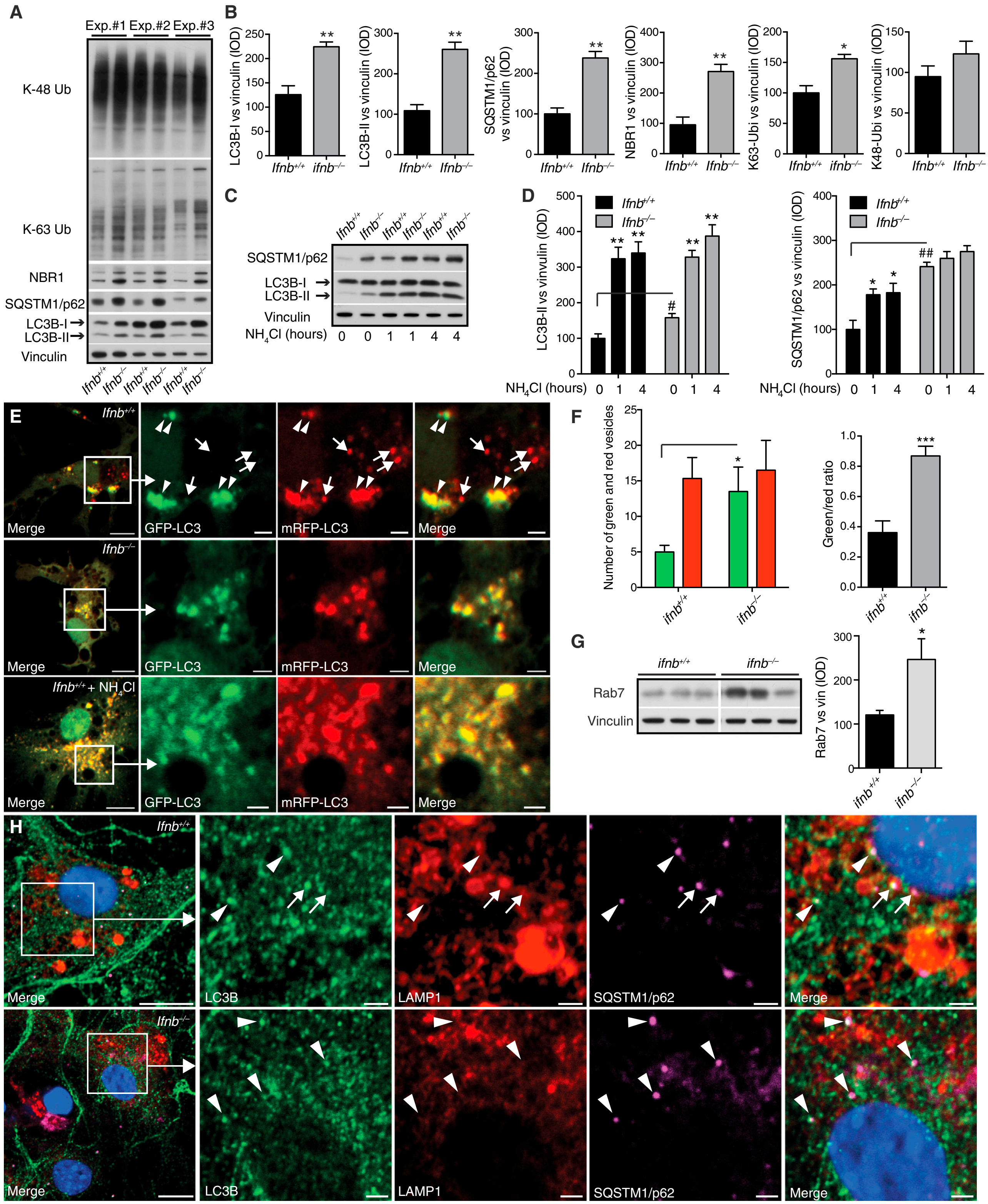

Figure 5. *Ifnb*$^{-/-}$ Neurons Have a Defect in Autophagy Maturation

(A–H) *Ifnb*$^{+/+}$ and *Ifnb*$^{-/-}$ primary CNs cultured for 21 days.

(A) WB from three independent experiments with antibodies as indicated.

(legend continued on next page)

α-syn in PD (Thenganatt and Jankovic, 2014). Overexpression of disease-associated proteins (Arima et al., 1999; Jellinger, 2000; Lippa et al., 1998) was not required to accumulate LB-like structures in *Ifnb*$^{-/-}$, *Ifnar*$^{-/-}$, or *nes*^{Cre}:*Ifnar*$^{fl/fl}$ neurons. *Ifnb* or *Ifnar* deletion was the sole trigger for α-syn-containing inclusion bodies resulting from defective autophagosome maturation. Treatment with rIFN-β promoted autophagy flux and increased α-syn clearance in *Ifnb*$^{+/+}$ and *Ifnb*$^{-/-}$ neurons, supporting IFN-β function in mutated proteins clearance, as suggested with ataxin 7 (Chort et al., 2013). Shared pathology of *Ifnb*$^{-/-}$ and *nes*^{Cre}:*Ifnar*$^{fl/fl}$ mice suggests that the pathogenesis is likely driven by initial changes in neuroectodermal neurons rather than systemic or local immune activation. In agreement, neurons are crucial for regulation of CNS inflammation (Liu et al., 2006).

Protein degradation defects are common in neurodegenerative pathologies (Davies et al., 1997; Hara et al., 2006; Komatsu et al., 2006). We found that endogenous neuronal IFN-β signaling is central in regulating protein degradation by autophagy, including clearing aged mitochondria. Accumulated aged and defective mitochondria may release reactive oxygen species, enhancing neuroinflammation and neuronal death (Chaturvedi and Flint Beal, 2013). Cognitive and motoric impairments in PD is associated with DA dysfunction (Narayanan et al., 2013). Excess cytosolic DA is degraded by monoamine oxidase (MAO) in the mitochondrial outer membrane and MAO defects might cause oxidative stress (Segura-Aguilar et al., 2014). Senescent and damaged mitochondria and reduced MAO mRNA were found in *Ifnb*$^{-/-}$ mice and reduced DOPAC in *Ifnb*$^{-/-}$ mice supported dysregulation of MAO and mitochondrial genes potentially contributing to neurotoxicity and DA neuron death.

Lack of endogenous IFN-β signaling was associated with spontaneous neurodegeneration, impaired motor coordination and cognition, and neuronal LB-like inclusions with aging as seen in most PD and DLB patients (Jellinger, 2008). Gene profiling supported essential IFN-β regulation of neuronal homeostasis. Neurotrophin was a top hit in GSEA and increased Tox mRNA, important for corticogenesis (Artegiani et al., 2015), supported IFN-β effects on prosurvival mechanisms.

We showed that *Ifnb* gene therapy reversed pathology in a familial PD model, by promoting autophagy and α-syn clearance, which preserved DA neurons and associated neurologic deficit. Our data strongly support an essential role for IFN-β signaling in preventing neurodegenerative pathology and suggest *Ifnb*$^{-/-}$ mice as a model for nonfamilial, sporadic neurodegenerative diseases, particularly PD and DLB, with potential for testing future therapies.

EXPERIMENTAL PROCEDURES

For detailed procedures, see Supplemental Experimental Procedures.

Mice and Cell Culture

Ifnb$^{-/-}$ mice (Erlandsson et al., 1998) were backcrossed 20 generations to B10.RIII or C57BL6. *Ifnar*$^{-/-}$ and *nes*^{Cre}:*Ifnar*$^{fl/fl}$ mice were in C57BL6 (Prinz et al., 2008). WT were *Ifnb*$^{+/-}$, *Ifnb*$^{+/+}$ littermates, or *Ifnar*$^{+/+}$ C57BL6 mice. C57BL/6JOlaHsd mice (Harlan Laboratories) with a spontaneous deletion of part of *Snca* (α-syn) were crossed with *Ifnb*$^{-/-}$ mice for *Scna*$^{-/-}$*Ifnb*$^{-/-}$. Mice were housed in standard facilities. Sex- and weight-matched mice were used in experiments performed in accordance with the ethical committees in Denmark and approved by our institutional review boards. CGNs were from 6- or 7-day-old cerebella and cortical neuron (CN) cultures from the cortex of 1-day-old mice.

Behavioral Measurements

Motor-coordination and -learning were evaluated with an accelerating RotaRod (TSE Systems GmbH) automatically recording time before fall. Neuromuscular strength was tested by forelimb hanging time on a bar. Heat and cold tail-pain sensitivity was measured by tail-flick latency time after exposure.

Spatial learning and reference memory were assessed with Morris water maze (Vorhees and Williams, 2006) with slight modifications. Swimming patterns were recorded with Ethovision 3.1 (Noldus Information Technology), measuring the time to reach a hidden platform during learning trials and frequency of platform position crossings during probe tests. Deficits prevalence were calculated as behavioral test scores of *Ifnb*$^{-/-}$ mice that deviated from the norm: mean value (SD/2) of the *Ifnb*$^{+/+}$ group (Table S1).

In the cylinder tests, asymmetry in forelimb use during vertical exploration was used as a validated measure of akinesia in hemiparkinsonian rodents.

Cloning

Mouse *Ifnb* pCR4IFNb was from transOMIC (accession no. BC119395). *Ifnb* was transferred to pCSII-GW via pCR8TOPOGW (Invitrogen) with conventional cloning techniques to generate pCSII-IFNb. PCSII (without insert) was generated by lambda recombination with an empty pCR8GW vector. Plasmid inserts were verified by sequencing.

Surgery with AAV6-hSCNA

AAV-GFP or AAV-human α-syn/hSNCA-WPRE (Vector Biolabs) together with lentiviral vectors pCSII-IFNb and pCSII control (3 μl for each virus) were injected unilaterally into 30 adult female Sprague Dawley rats (Taconic; 225–250 g at surgery). Virus was infused at 0.2 μl/min as described (Decressac et al., 2013).

Immunohistochemistry, Immunofluorescence, and Transmission Electron Microscopy

For immunohistochemistry (IHC) and immunofluorescence (IF), either mice were perfused and brains fixed in 4% paraformaldehyde (PFA) and paraffin

(B) Quantified IOD of WB bands normalized to vinculin. Data are mean ± SEM, n = 3.
(C) WB of CNs with or without NH_4Cl (20 mM) for 1 or 4 hr.
(D) Quantified IOD of WB. Data are mean ± SEM of n = 3, */#p < 0.05, **/##p < 0.01 by one-way ANOVA. *Within and # between genotype differences after NH_4Cl treatment.
(E) CNs expressing mRFP-GFP-LC3B with or without NH_4Cl for 2 hr. Arrowheads, colocalized GFP and mRFP (autophagosomes); arrows, mRFP-only vesicles (autolysosomes). Scale bars, 10 μm; 2 μm in inserts.
(F) Graphs, mean number of autophagosomes and autolysosomes/cell and vesicle ratio ± SEM, n = 3.
(G) WB of Rab7. Graph, IOD of WB. Data are mean ± SEM of n = 3.
(H) IF showing LC3B and p62 colocalized in autophagosomes (arrowheads) and LC3B, LAMP1, and p62 colocalizing in autolysosomes (arrows). Scale bars, 10 μm; 2 μm in inserts.
For (B), (F), and (G), *p < 0.05, *p < 0.01, ***p < 0.001 by unpaired Student's t test. See also Figure S5.

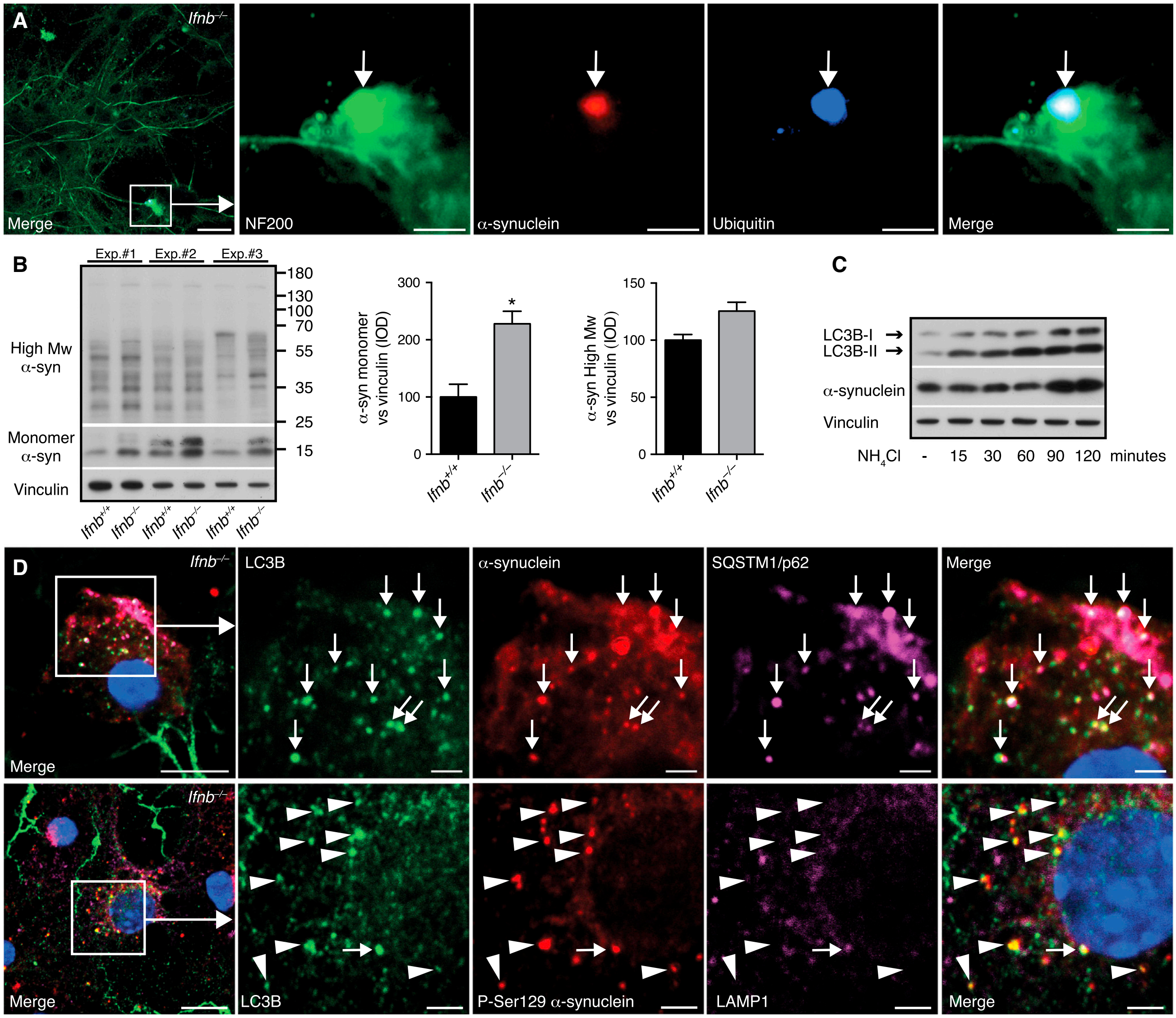

Figure 6. Autophagy Block Causes α-syn Accumulation

(A) IF of *Ifnb*$^{-/-}$ CN with inclusion body (arrow). Scale bar, 20 μm; 2 μm in insert.

(B) WB and quantified IOD bands of monomeric and high-molecular-weight (Mw) α-syn. Data are mean ± SEM, n = 3. $^{*}p < 0.05$ by unpaired Student's t test.

(C) WB of *Ifnb*$^{+/+}$ CNs treated with 20 mM NH_4Cl.

(D) IF of CNs from *Ifnb*$^{-/-}$ mice. Arrows, triple colocalizing vesicular structures; arrowheads, LC3B and phosphorylated (Ser129) α-syn-positive vesicular structures. Scale bars, 10 μm; 2 μm in inserts. See also Figure S6.

embedded or brains were dissected and snap-frozen before sectioning. In vitro neuronal cultures were fixed in 4% PFA before staining. Tissues and cells were stained as described (Liu et al., 2014).

For neurogenesis and BrdU staining, mice were injected intraperitoneally (i.p.) once per day (75 μg/g body weight) for 5 consecutive days and sacrificed 2 hr after last injection. Brains were processed and immunostained for BrdU and doublecortin (DCX).

MMP was measured by adding tetramethylrhodamine ethyl ester (TMRE) and Hoechst (Life Technologies) to primary CNs.

IF images were taken with a Zeiss LSM510 confocal scanning microscope and IN Cell Analyzer 2200 automated microscope. IHC images were taken with a NanoZoomer 2.0-HT digital slide scanner or Olympus BX51 microscope. Images were quantified with ImageJ (Fiji version), IN Cell Investigator, CellProfiler, Zeiss Zen, and Adobe Photoshop.

In situ apoptosis detection was with TUNEL kits (Calbiochem) with Hoechst counterstaining or DAB substrate and methyl green counterstaining kits (R&D Systems).

For transmission electron microscopy (TEM), 12-month-old mice were cardiac perfused with 2% PFA and 0.2% glutaraldehyde and paratenial, and central medial thalamic nuclei were dissected and processed for epon embedding and ultrathin sectioning. Samples were incubated with primary α-syn antibodies (Leica) and biotinylated (Vector Laboratories) or 1.4-nm gold-labeled secondary antibodies (Nanoprobes) and embedded in epoxy resin. Ultra-thin sections were analyzed in a Philips CM100 electron microscope.

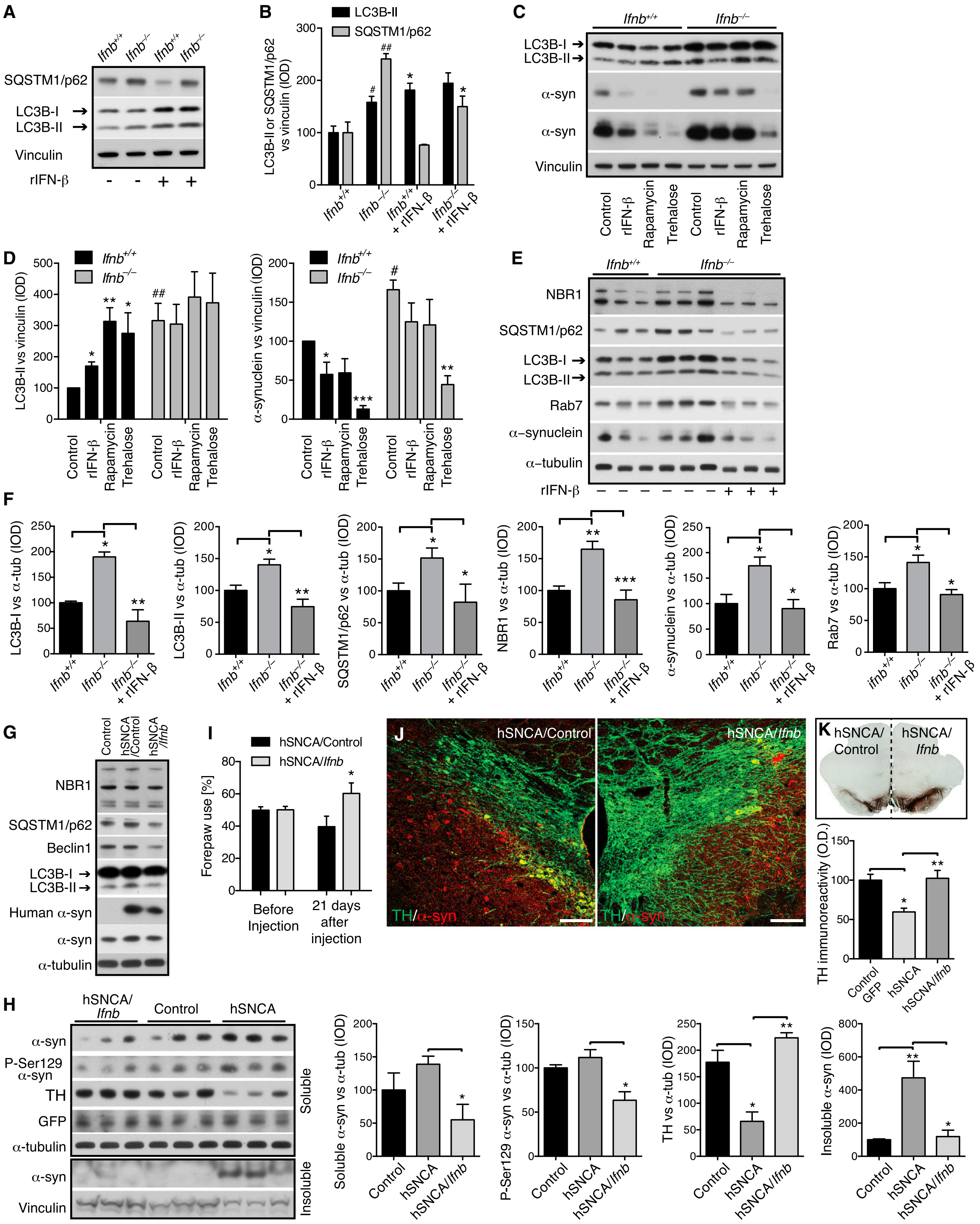

(legend on next page)

Transfection and Plasmids
CNs were transfected with mRFP-GFP-LC3 using Lipofectamine 2000 (Life Technologies) according to the manufacturer's description for live-cell confocal imaging.

Stereological Analysis
The optical fractionator method was used to estimate neuron and glial numbers in the hippocampus of 80-μm mouse brain sections stained with H&E. Pointcounting techniques, based on the Cavalieri principle, were used to estimate hippocampal volume on one side of the brain.

Real-Time PCR
Total RNA was isolated using a QIAGEN kit (QIAGEN), reverse transcribed into cDNA, amplified, and quantified by SYBR Green (Bio-Rad) detection. Relative mRNA expression was normalized with glyceraldehyde 3-phosphate dehydrogenase (*Gapdh*) gene.

High-Performance Liquid Chromatography
Striatum was dissected and homogenized. Filtered supernatant was examined for DA, DOPAC, and HVA levels by reversed-phase HPLCy (Decressac et al., 2013).

Western Blots
Samples were lysed in 1% Triton X-100 (Sigma) and insoluble brain pellets were sonicated in UREA/SDS and processed as described (Ejlerskov et al., 2013).

Affymetrix Microarrays
RNA was extracted with TRI (Sigma) and DNase I (Invitrogen) from 3-day-old *Ifnb*$^{+/+}$ and *Ifnb*$^{-/-}$ CGN cultures in triplicate with or without 24 hr rIFN-β (100 U/ml). Affymetrix 430 2.0 microarray chip (SCIBLU, Affymetrix) data were analyzed with Arraystar 3 (DNA STAR) and quantile-normalized and processed by the RMA (Affymetrix) algorithm. We $\log_2$-transformed intensity values, and normal-distributed data were tested in unpaired two-tailed Student's t tests, filtering for differential regulation confidence of 95% ($p < 0.05$). Venn diagrams were created with oneChannelGUI (Bioconductor). Quantile-normalized RMA-treated data selected using a 1.4-fold cutoff were analyzed with Ingenuity Pathway Analysis software.

GSEA data heatmaps were generated by extracting lists of core enriched genes from GSEA pathway analysis in R using Heatmap2 (Data S1).

Comparisons of *Ifnb*$^{-/-}$ CGN gene profiles and published PD and HD models using Affymetrix data (GSE4758, GSE9038) were quantile-normalized together and summarized in R using RMA algorithms from the Affy-package. Differential expression was determined individually within each experiment for our data and published PD and HD models by comparing control/WT samples with transgenic samples using standard ANOVA.

Statistical Analysis
Data were analyzed with unpaired and paired two-tailed Student's t tests, ANOVA, and Mann-Whitney U, and Kruskal-Wallis tests. $p < 0.05$ was significant. Error bars are SEM.

ACCESSION NUMBERS

The accession number for the raw microarray data reported in this paper is Gene Expression Omnibus: GSE63815.

SUPPLEMENTAL INFORMATION

Supplemental Information includes Supplemental Experimental Procedures, six figures, two tables, and a data file and can be found with this article online at http://dx.doi.org/10.1016/j.cell.2015.08.069.

AUTHOR CONTRIBUTIONS

P.E., J.G.H., J.W., R.C., M.K., Y.L., G.P., K.K., M.A., C.F.R., and K.R. did experiments and analyzed and prepared data; B.P. contributed to stereological studies; T.G., D.L., and M.P. did experiments including immunolabeling-EM and *Ifnar*$^{-/-}$ and *nes*^{Cre}:*Ifnar*$^{fl/fl}$ mouse analysis; D.C.R. contributed material and designed some protein degradation experiments and S.I.-N. designed and supervised the study, analyzed and interpreted data, and wrote the manuscript. All authors read and contributed to the final manuscript.

ACKNOWLEDGMENTS

Support to S.I.-N. was from Danish Council For Independent Research (DFF)-Medical Sciences, Alzheimer-forskningsfonden, Danish Multiple Sclerosis Society, Danish Cancer Society, and Lundbeck Foundation. D.C.R. is a Wellcome Trust Principal Research Fellow. The authors thank Marja Jäättelä for help establishing lysosomal assays, Janne Kock for efficient experimental animal facility arrangement, Elin Pietras for help with IN Cell analysis, Mohamoud Osman, Alexandra Müller, Ana Rita Antunes, Sigrun Nestel, and Barbara Joch for technical assistance, and Klaus Qvortrup for electron microscopy help.

Received: December 4, 2014
Revised: May 20, 2015
Accepted: August 13, 2015
Published: October 8, 2015

REFERENCES

Arima, K., Hirai, S., Sunohara, N., Aoto, K., Izumiyama, Y., Uéda, K., Ikeda, K., and Kawai, M. (1999). Cellular co-localization of phosphorylated tau- and

Figure 7. IFN-β Treatment Promotes Autophagy Flux and Reduces α-syn Accumulation
(A–F) *Ifnb*$^{+/+}$ and *Ifnb*$^{-/-}$ primary CNs cultured for 21 days.
(A) WB of CN with or without rIFN-β (100 U/ml) for 22 hr.
(B) Quantified IOD of WB bands. Graphs, mean ± SEM, n = 3; */#p < 0.05; ##p < 0.01 by one-way ANOVA. #Between genotypes and *after rIFN-β in each group.
(C) WB of LC3, α-syn (two exposures), and vinculin from untreated or rIFN-β (100 U/ml), rapamycin (1 μM), or trehalose (50 mM) -treated CNs (24 hr).
(D) Quantified IOD of WB bands with mean ± SEM, n = 4–5. *After treatment within genotype and #between untreated control genotypes.
(E) WB of CNs with 30 U/ml rIFN-β (every 3–4 days) and 100 U/ml the last 24 hr.
(F) Quantified IOD. Data are mean ± SEM, n = 4–6.
(G and H) WB of rat BG 10 days post AAV- and lentiviral-control or AAV-hSNCA co-injection with lentivirus-*Ifnb* or lentivirus-control vector. Antibodies were against autophagy markers, human α-syn, or human and rat α-syn, pSer129-α-syn, TH, and GFP (to confirm AAV and lentiviral expression). Graphs, mean ± SEM, n = 3/group.
(I) Right and left forepaw use in rats before and 21 days after AAV injection of hSNCA with lentivirus-control or lentivirus-*Ifnb* in left and right SN brain hemisphere, respectively. Graph, mean ± SD, n = 3–5 rats/group. *p < 0.05 by Student's t test.
(J) IF of rat brain; right and left hemisphere 21 days after virus injection. Scale bar, 100 μm.
(K) IHC of rat 21 days after virus injection as in (I) with SN TH immunoreactivity. Graph, mean (OD) ± SEM. TH immunoreactivity of control AAV/lentivirus, AAV-hSCNA/lentivirus control, or AAV hSCNA/lentivirus-*Ifnb*, n = 3–5 rats/group.
For (B), (D), (F), (H), and (K), */#p < 0.05, **p < 0.01, ***p < 0.001 by one-way ANOVA.

NACP/alpha-synuclein-epitopes in lewy bodies in sporadic Parkinson's disease and in dementia with Lewy bodies. Brain Res. *843*, 53–61.

Artegiani, B., de Jesus Domingues, A.M., Bragado Alonso, S., Brandl, E., Massalini, S., Dahl, A., and Calegari, F. (2015). Tox: a multifunctional transcription factor and novel regulator of mammalian corticogenesis. EMBO J. *34*, 896–910.

Bartels, T., Choi, J.G., and Selkoe, D.J. (2011). α-Synuclein occurs physiologically as a helically folded tetramer that resists aggregation. Nature *477*, 107–110.

Baruch, K., Deczkowska, A., David, E., Castellano, J.M., Miller, O., Kertser, A., Berkutzki, T., Barnett-Itzhaki, Z., Bezalel, D., Wyss-Coray, T., et al. (2014). Aging. Aging-induced type I interferon response at the choroid plexus negatively affects brain function. Science *346*, 89–93.

Brochard, V., Combadière, B., Prigent, A., Laouar, Y., Perrin, A., Beray-Berthat, V., Bonduelle, O., Alvarez-Fischer, D., Callebert, J., Launay, J.M., et al. (2009). Infiltration of CD4+ lymphocytes into the brain contributes to neurodegeneration in a mouse model of Parkinson disease. J. Clin. Invest. *119*, 182–192.

Chaturvedi, R.K., and Flint Beal, M. (2013). Mitochondrial diseases of the brain. Free Radic. Biol. Med. *63*, 1–29.

Chort, A., Alves, S., Marinello, M., Dufresnois, B., Dornbierer, J.G., Tesson, C., Latouche, M., Baker, D.P., Barkats, M., El Hachimi, K.H., et al. (2013). Interferon β induces clearance of mutant ataxin 7 and improves locomotion in SCA7 knock-in mice. Brain *136*, 1732–1745.

Congdon, E.E., Gu, J., Sait, H.B., and Sigurdsson, E.M. (2013). Antibody uptake into neurons occurs primarily via clathrin-dependent Fcγ receptor endocytosis and is a prerequisite for acute tau protein clearance. J. Biol. Chem. *288*, 35452–35465.

Davies, S.W., Turmaine, M., Cozens, B.A., DiFiglia, M., Sharp, A.H., Ross, C.A., Scherzinger, E., Wanker, E.E., Mangiarini, L., and Bates, G.P. (1997). Formation of neuronal intranuclear inclusions underlies the neurological dysfunction in mice transgenic for the HD mutation. Cell *90*, 537–548.

Decressac, M., Mattsson, B., Weikop, P., Lundblad, M., Jakobsson, J., and Björklund, A. (2013). TFEB-mediated autophagy rescues midbrain dopamine neurons from α-synuclein toxicity. Proc. Natl. Acad. Sci. USA *110*, E1817–E1826.

Ejlerskov, P., Rasmussen, I., Nielsen, T.T., Bergström, A.L., Tohyama, Y., Jensen, P.H., and Vilhardt, F. (2013). Tubulin polymerization-promoting protein (TPPP/p25α) promotes unconventional secretion of α-synuclein through exophagy by impairing autophagosome-lysosome fusion. J. Biol. Chem. *288*, 17313–17335.

Erlandsson, L., Blumenthal, R., Eloranta, M.L., Engel, H., Alm, G., Weiss, S., and Leanderson, T. (1998). Interferon-beta is required for interferon-alpha production in mouse fibroblasts. Curr. Biol. *8*, 223–226.

Fossale, E., Seong, I.S., Coser, K.R., Shioda, T., Kohane, I.S., Wheeler, V.C., Gusella, J.F., MacDonald, M.E., and Lee, J.M. (2011). Differential effects of the Huntington's disease CAG mutation in striatum and cerebellum are quantitative not qualitative. Hum. Mol. Genet. *20*, 4258–4267.

Francis, P.T. (2009). Biochemical and pathological correlates of cognitive and behavioural change in DLB/PDD. J. Neurol. *256* (*Suppl 3*), 280–285.

Goedert, M., Spillantini, M.G., Del Tredici, K., and Braak, H. (2013). 100 years of Lewy pathology. Nat. Rev. Neurol. *9*, 13–24.

Gutierrez, M.G., Munafó, D.B., Berón, W., and Colombo, M.I. (2004). Rab7 is required for the normal progression of the autophagic pathway in mammalian cells. J. Cell Sci. *117*, 2687–2697.

Hara, T., Nakamura, K., Matsui, M., Yamamoto, A., Nakahara, Y., Suzuki-Migishima, R., Yokoyama, M., Mishima, K., Saito, I., Okano, H., and Mizushima, N. (2006). Suppression of basal autophagy in neural cells causes neurodegenerative disease in mice. Nature *441*, 885–889.

Harris, H., and Rubinsztein, D.C. (2012). Control of autophagy as a therapy for neurodegenerative disease. Nat. Rev. Neurol. *8*, 108–117.

Hyttinen, J.M., Niittykoski, M., Salminen, A., and Kaarniranta, K. (2013). Maturation of autophagosomes and endosomes: a key role for Rab7. Biochim. Biophys. Acta *1833*, 503–510.

Jellinger, K.A. (2000). Morphological substrates of mental dysfunction in Lewy body disease: an update. J. Neural Transm. Suppl. *59*, 185–212.

Jellinger, K.A. (2008). Neuropathological aspects of Alzheimer disease, Parkinson disease and frontotemporal dementia. Neurodegener. Dis. *5*, 118–121.

Jellinger, K.A., and Attems, J. (2008). Prevalence and impact of vascular and Alzheimer pathologies in Lewy body disease. Acta Neuropathol. *115*, 427–436.

Komatsu, M., Waguri, S., Chiba, T., Murata, S., Iwata, J., Tanida, I., Ueno, T., Koike, M., Uchiyama, Y., Kominami, E., and Tanaka, K. (2006). Loss of autophagy in the central nervous system causes neurodegeneration in mice. Nature *441*, 880–884.

Lee, Y.K., Kwak, D.H., Oh, K.W., Nam, S.Y., Lee, B.J., Yun, Y.W., Kim, Y.B., Han, S.B., and Hong, J.T. (2009). CCR5 deficiency induces astrocyte activation, Abeta deposit and impaired memory function. Neurobiol. Learn. Mem. *92*, 356–363.

Linnartz, B., Kopatz, J., Tenner, A.J., and Neumann, H. (2012). Sialic acid on the neuronal glycocalyx prevents complement C1 binding and complement receptor-3-mediated removal by microglia. J. Neurosci. *32*, 946–952.

Lippa, C.F., Fujiwara, H., Mann, D.M., Giasson, B., Baba, M., Schmidt, M.L., Nee, L.E., O'Connell, B., Pollen, D.A., St George-Hyslop, P., et al. (1998). Lewy bodies contain altered alpha-synuclein in brains of many familial Alzheimer's disease patients with mutations in presenilin and amyloid precursor protein genes. Am. J. Pathol. *153*, 1365–1370.

Liu, Y., Teige, I., Birnir, B., and Issazadeh-Navikas, S. (2006). Neuron-mediated generation of regulatory T cells from encephalitogenic T cells suppresses EAE. Nat. Med. *12*, 518–525.

Liu, Y., Carlsson, R., Ambjørn, M., Hasan, M., Badn, W., Darabi, A., Siesjö, P., and Issazadeh-Navikas, S. (2013). PD-L1 expression by neurons nearby tumors indicates better prognosis in glioblastoma patients. J. Neurosci. *33*, 14231–14245.

Liu, Y., Carlsson, R., Comabella, M., Wang, J., Kosicki, M., Carrion, B., Hasan, M., Wu, X., Montalban, X., Dziegiel, M.H., et al. (2014). FoxA1 directs the lineage and immunosuppressive properties of a novel regulatory T cell population in EAE and MS. Nat. Med. *20*, 272–282.

Maccioni, R.B., Rojo, L.E., Fernández, J.A., and Kuljis, R.O. (2009). The role of neuroimmunomodulation in Alzheimer's disease. Ann. N Y Acad. Sci. *1153*, 240–246.

Mori, F., Piao, Y.S., Hayashi, S., Fujiwara, H., Hasegawa, M., Yoshimoto, M., Iwatsubo, T., Takahashi, H., and Wakabayashi, K. (2003). Alpha-synuclein accumulates in Purkinje cells in Lewy body disease but not in multiple system atrophy. J. Neuropathol. Exp. Neurol. *62*, 812–819.

Mrak, R.E., and Griffin, W.S. (2007). Dementia with Lewy bodies: Definition, diagnosis, and pathogenic relationship to Alzheimer's disease. Neuropsychiatr. Dis. Treat. *3*, 619–625.

Narayanan, N.S., Rodnitzky, R.L., and Uc, E.Y. (2013). Prefrontal dopamine signaling and cognitive symptoms of Parkinson's disease. Rev. Neurosci. *24*, 267–278.

Nelson, A.L. (2010). Antibody fragments: hope and hype. MAbs *2*, 77–83.

Prinz, M., Schmidt, H., Mildner, A., Knobeloch, K.P., Hanisch, U.K., Raasch, J., Merkler, D., Detje, C., Gutcher, I., Mages, J., et al. (2008). Distinct and nonredundant in vivo functions of IFNAR on myeloid cells limit autoimmunity in the central nervous system. Immunity *28*, 675–686.

Rockenstein, E., Nuber, S., Overk, C.R., Ubhi, K., Mante, M., Patrick, C., Adame, A., Trejo-Morales, M., Gerez, J., Picotti, P., et al. (2014). Accumulation of oligomer-prone α-synuclein exacerbates synaptic and neuronal degeneration in vivo. Brain *137*, 1496–1513.

Segura-Aguilar, J., Paris, I., Muñoz, P., Ferrari, E., Zecca, L., and Zucca, F.A. (2014). Protective and toxic roles of dopamine in Parkinson's disease. J. Neurochem. *129*, 898–915.

Sheehan, K.C., Lai, K.S., Dunn, G.P., Bruce, A.T., Diamond, M.S., Heutel, J.D., Dungo-Arthur, C., Carrero, J.A., White, J.M., Hertzog, P.J., and Schreiber, R.D.

(2006). Blocking monoclonal antibodies specific for mouse IFN-alpha/beta receptor subunit 1 (IFNAR-1) from mice immunized by in vivo hydrodynamic transfection. J. Interferon Cytokine Res. *26*, 804–819.

Shulman, J.M., and De Jager, P.L. (2009). Evidence for a common pathway linking neurodegenerative diseases. Nat. Genet. *41*, 1261–1262.

Tansey, M.G., Frank-Cannon, T.C., McCoy, M.K., Lee, J.K., Martinez, T.N., McAlpine, F.E., Ruhn, K.A., and Tran, T.A. (2008). Neuroinflammation in Parkinson's disease: is there sufficient evidence for mechanism-based interventional therapy? Front. Biosci. *13*, 709–717.

Teige, I., Treschow, A., Teige, A., Mattsson, R., Navikas, V., Leanderson, T., Holmdahl, R., and Issazadeh-Navikas, S. (2003). IFN-beta gene deletion leads to augmented and chronic demyelinating experimental autoimmune encephalomyelitis. J. Immunol. *170*, 4776–4784.

Teige, I., Liu, Y., and Issazadeh-Navikas, S. (2006). IFN-beta inhibits T cell activation capacity of central nervous system APCs. J. Immunol. *177*, 3542–3553.

Thenganatt, M.A., and Jankovic, J. (2014). Parkinson disease subtypes. JAMA Neurol. *71*, 499–504.

Vorhees, C.V., and Williams, M.T. (2006). Morris water maze: procedures for assessing spatial and related forms of learning and memory. Nat. Protoc. *1*, 848–858.

Wang, W., Perovic, I., Chittuluru, J., Kaganovich, A., Nguyen, L.T., Liao, J., Auclair, J.R., Johnson, D., Landeru, A., Simorellis, A.K., et al. (2011). A soluble α-synuclein construct forms a dynamic tetramer. Proc. Natl. Acad. Sci. USA *108*, 17797–17802.

Yong, V.W., Chabot, S., Stuve, O., and Williams, G. (1998). Interferon beta in the treatment of multiple sclerosis: mechanisms of action. Neurology *51*, 682–689.

Supplemental Figures

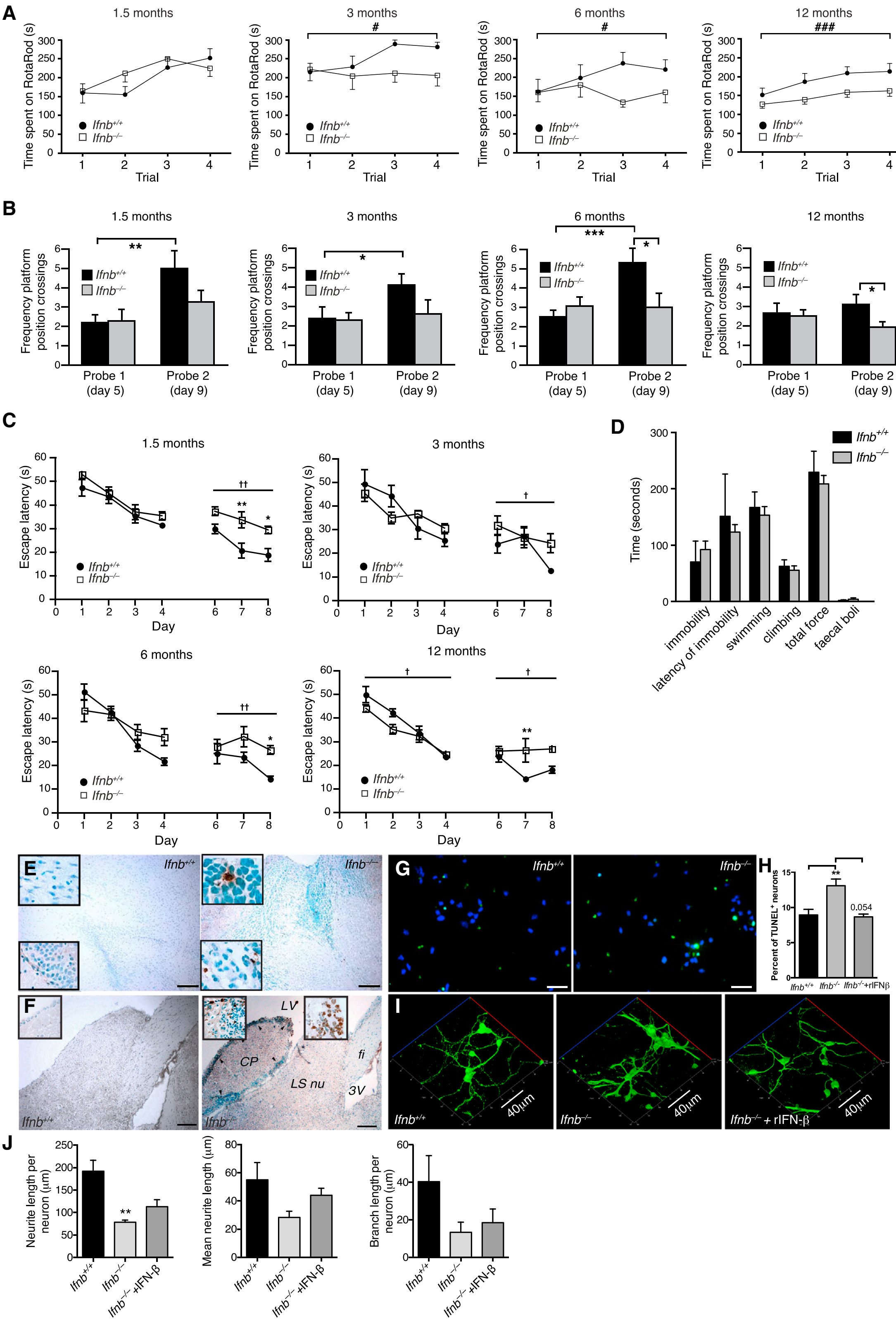

(legend on next page)

Figure S1. *Ifnb*$^{-/-}$ Mice Show Behavioral Deficits, Neuronal Apoptosis, and Neurite Retraction, Related to Figure 1

(A) Motor-coordination and -learning as assessed by seconds (s) spent on RotaRod for each trial. Data are mean ± SEM, 8–13 mice per group. Two-way ANOVA; $^{\#}p < 0.05$, and $^{\#\#\#}p < 0.001$ were conducted to assess the effects of genotype and trials.

(B and C) Performance of *Ifnb*$^{+/+}$ and *Ifnb*$^{-/-}$ mice at different ages in Morris Water Maze on probe day 1 and 2.

(B) Graphs show mean frequency of platform position crossings. Data are mean ± SEM of 8–21 mice per group. Student's t test was used to assess differences in frequency of platform position crossings between probe trials within each genotype and age group, and between genotypes of the same age within a probe trial. $^{*}p < 0.05$; $^{**}p < 0.01$; $^{***}p < 0.001$.

(C) Escape latency in seconds (s). $^{\dagger}p < 0.05$ and $^{\dagger\dagger}p < 0.01$ by two-way repeated-measures ANOVA comparing learning curves by genotype; $^{*}p < 0.05$ by Bonferroni post hoc test shows significant genotype effect within each learning day (trials). Data are mean ± SEM of 8–21 mice per group.

(D) Forced swimming test of *Ifnb*$^{+/+}$ and *Ifnb*$^{-/-}$ mice. Data represent mean ± SEM. Student's t test showed no significant differences between the genotypes.

(E and F) TUNEL staining of brain with methyl green counterstaining for nuclei showing (E) 1.5-month-old olfactory bulb areas and (F) 12-month-old *caudate putamen*. TUNEL-positive staining (arrows) are present in *Ifnb*$^{-/-}$ but not in *Ifnb*$^{+/+}$. CP: caudate putamen; LS nu: lateral septal nucleus; LV: left ventricle; 3V:3rd ventricle; fi: fimbria. Scale bar, 200 μm.

(G) IF images showing TUNEL staining of cerebellar granular neurons (CGNs) from *Ifnb*$^{+/+}$ and *Ifnb*$^{-/-}$ mice. Scale bar, 50 μm.

(H) Percentage of TUNEL-positive neurons (in vitro cultured CGNs) representing mean ± SEM from n = 3; 6-10 images were quantified from each experiment. $^{**}p < 0.01$ by unpaired Student's t test.

(I) 3D projected confocal images of CGNs with or without 24 hr of recombinant(r)IFN-β treatment (100 U/ml) showing NF200 (green). Scale bar, 40 μm.

(J) Quantification of neurite length per neuron, mean neurite length, and mean branching length of cultured CGN from *Ifnb*$^{+/+}$, *Ifnb*$^{-/-}$, and *Ifnb*$^{-/-}$ treated with rIFN-β (30U/ml) for the whole culture period. Graphs represents mean ± SEM of one representative experiment where 4-5 images containing 3-21 individual neurons were quantified. $^{*}p < 0.05$ by Student's t test.

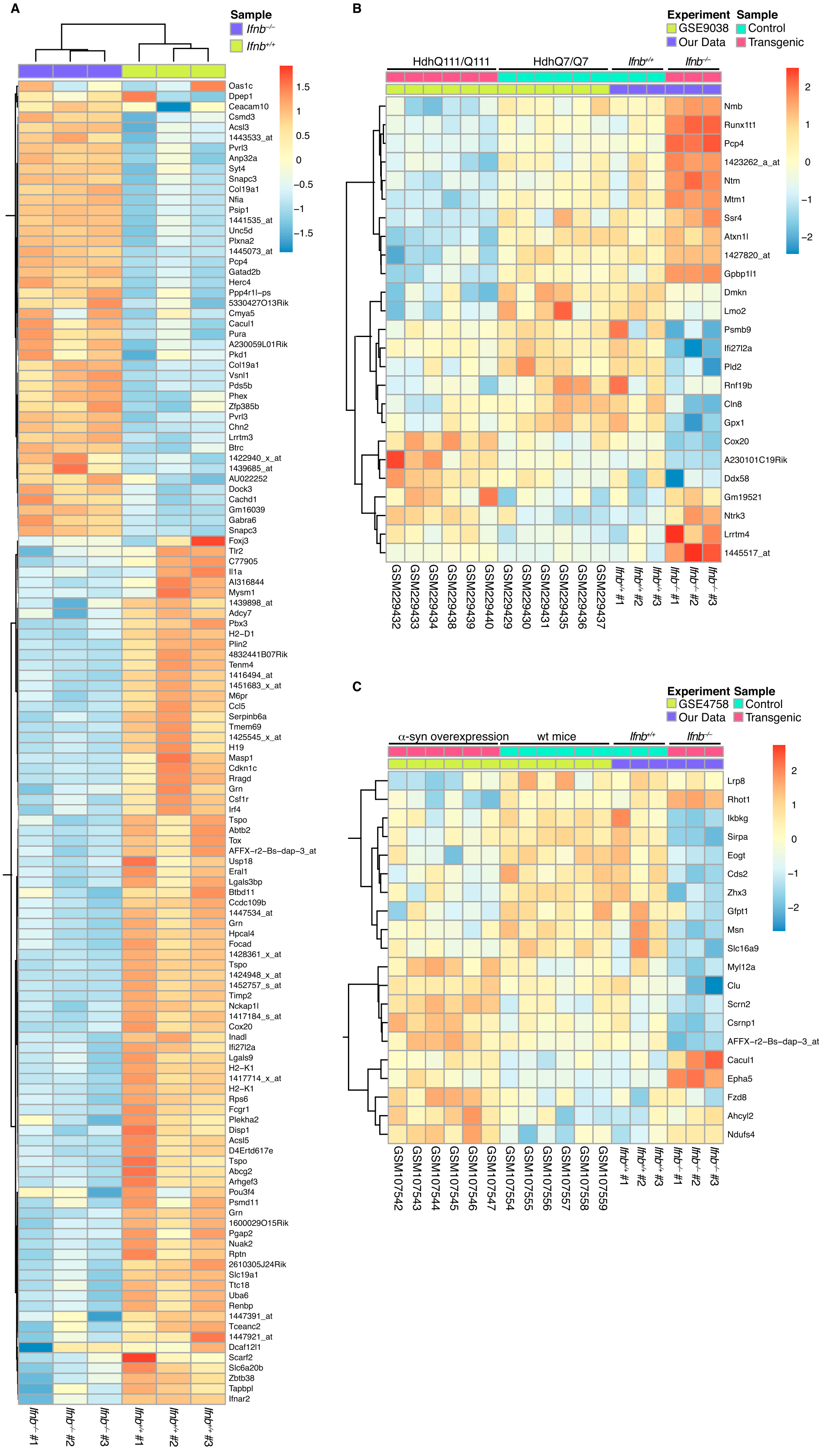

(legend on next page)

Figure S2. Gene Signature of *Ifnb*$^{-/-}$ Neurons and Heatmaps Comparing *Ifnb*$^{-/-}$ Mice with Mice Models of Huntington's and Parkinson's Disease, Related to Figure 2

(A) Heatmap of genes differentially expressed in *Ifnb*$^{-/-}$ and *Ifnb*$^{+/+}$ cerebellar granular neurons (CGNs).

(B and C) Heatmaps comparing differentially expressed genes in *Ifnb*$^{-/-}$ and *Ifnb*$^{+/+}$ CGNs with (B) Huntington's disease affymetrix data (GSE9038) and (C) Parkinson's disease affymetrix data (GSE4758). Identification of differential expressed genes was performed individually within each experiment (in (A) $p < 0.001$; in B-C) $p < 0.01$); n = 3-5.

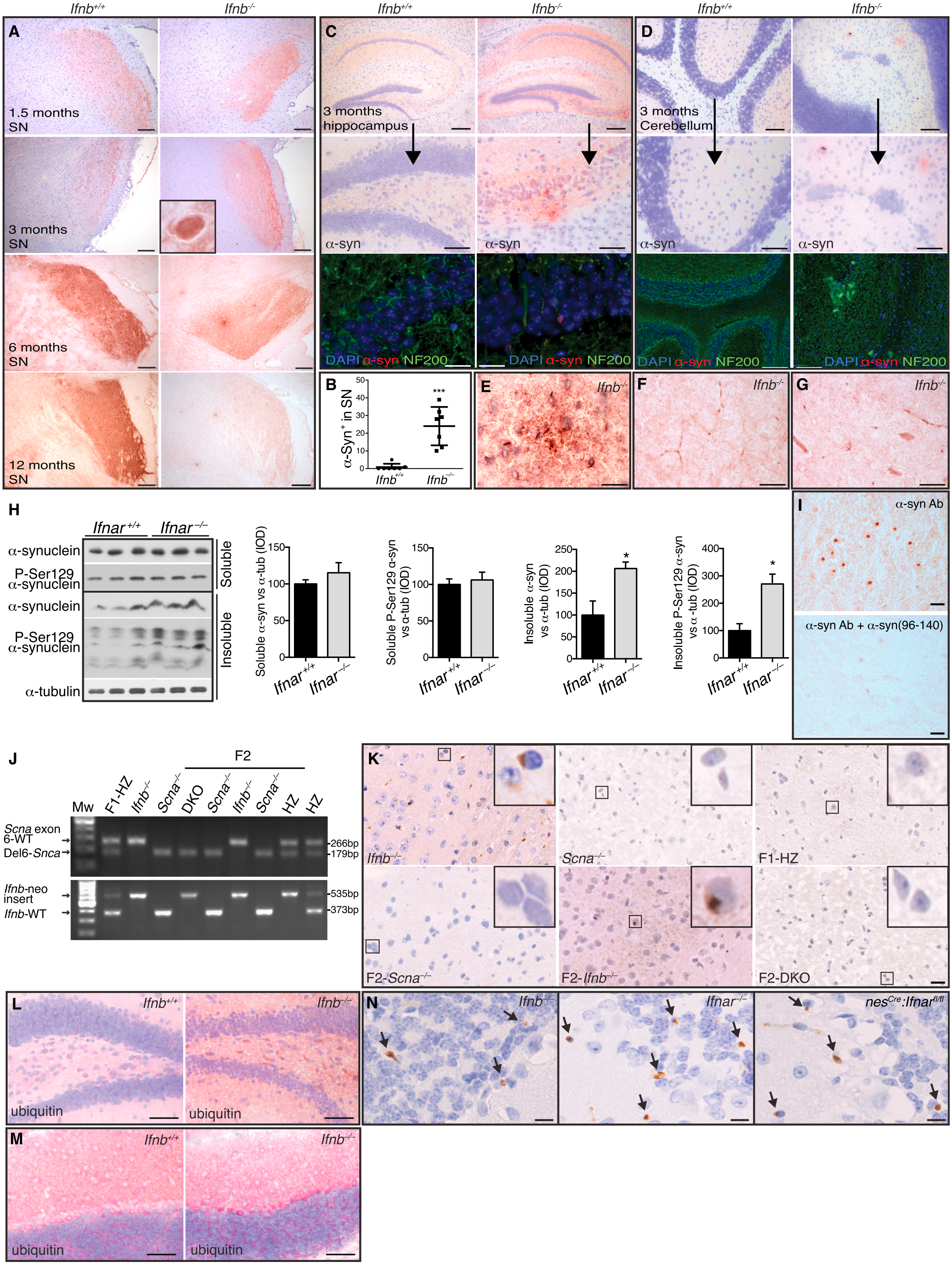

(legend on next page)

Cell

Figure S3. Lack of IFN-β Signaling Causes Widespread α-syn Accumulation in the Brain, Related to Figure 3

(A) α-syn immunostaining (IHC) of snap frozen brains from *Ifnb*$^{+/+}$ and *Ifnb*$^{-/-}$ showing images of substantia nigra (SN) at 1.5, 3, 6 and 12 months of age. Scale bar, 100 μm.

(B) Quantification of α-syn aggregates in *Ifnb*$^{+/+}$ and *Ifnb*$^{-/-}$ SN at 3 months.

(C and D) α-syn IHC staining (upper panels) and IF (lower panels) for α-syn (red), NF200 (green) and DAPI (blue) in hippocampus and cerebellum of *Ifnb*$^{+/+}$ and *Ifnb*$^{-/-}$ at 3 months. Scale bar, 100 μm; upper panels, and 20 μm; lower panels IHC and IF.

(E) α-syn aggregates in thalamus of 3 months old *Ifnb*$^{-/-}$ mice. Scale bar, 10 μm.

(F and G) α-syn^{+} neurites in brainstem and subthalamic region, respectively. Scale bar, 10 μm.

(H) WB (TX-100 soluble and insoluble) from basal ganglia of 8-month-old *Ifnar*$^{+/+}$ and *Ifnar*$^{-/-}$ mice, and quantified integrated optical density (IOD) of α-syn and phosphorylated (Ser129) α-syn normalized to α-tubulin. Graphs represents mean $\pm$ SEM, n = 3.

(I) 12 months old *Ifnb*$^{-/-}$ thalamus stained for α-syn and lower panel stained with excessive α-syn$_{96\text{-}140}$ peptide to block binding of the two utilized antibodies toward α-syn; i.e., sheep anti-mouse (Millipore) (I) and rabbit anti-α-syn (Abcam) (not shown). Scale bar, 20 μm.

(J) Genomic PCR screening of mice for *Scna* and *Ifnb* genes.

(K) IHC images showing α-syn staining of snap-frozen brain cryosections of parental; *Ifnb*$^{-/-}$, *Scna*$^{-/-}$, F1 Heterozygots (F1-HZ) and F2; DKO (*Ifnb*$^{-/-}$*Scna*$^{-/-}$ mice), *Ifnb*$^{-/-}$, and *Scna*$^{-/-}$ littermates. Scale bar, 10 μm.

(L and M) Ubiquitin IHC of snap frozen brains from *Ifnb*$^{+/+}$ and *Ifnb*$^{-/-}$ showing hippocampus (L) and cerebellum (M) at 3 months of age. Scale bar, 100 μm.

(N) IHC images of perfused and paraffin-embedded brain sections showing α-syn positive staining (yellowish brown) in cerebellar molecular and granular layers (blue hematoxyline counter staining) of *Ifnb*$^{-/-}$, *Ifnar*$^{-/-}$ and *nes*^{Cre}:*Ifnar*$^{fl/fl}$ mice.

Scale bar, 10 μm. For (B) and (H) $^{*}p < 0.05$ and $^{***}p < 0.001$ by unpaired Student's t test.

Cell

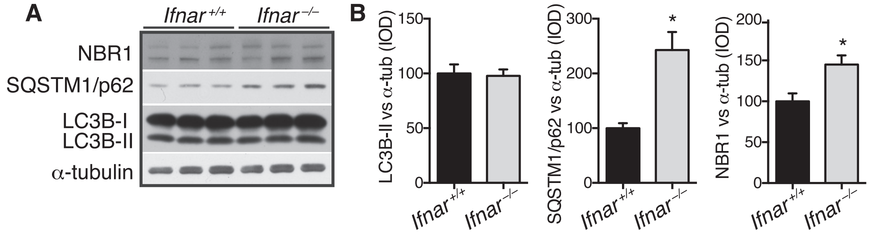

Figure S4. Late-Stage Degradative Autophagosomes Accumulate in *Ifnar*$^{-/-}$ Neurons, Related to Figure 4

(A and B) (A) WB of brain extracts from 8-month-old *Ifnar*$^{+/+}$ and *Ifnar*$^{-/-}$ mice immunoblotted against SQSTM1/p62, LC3B, α-tubulin, and NBR1 and (B) quantified IOD. Data are mean ± SEM of n = 3, *p < 0.05 by unpaired Students t test.

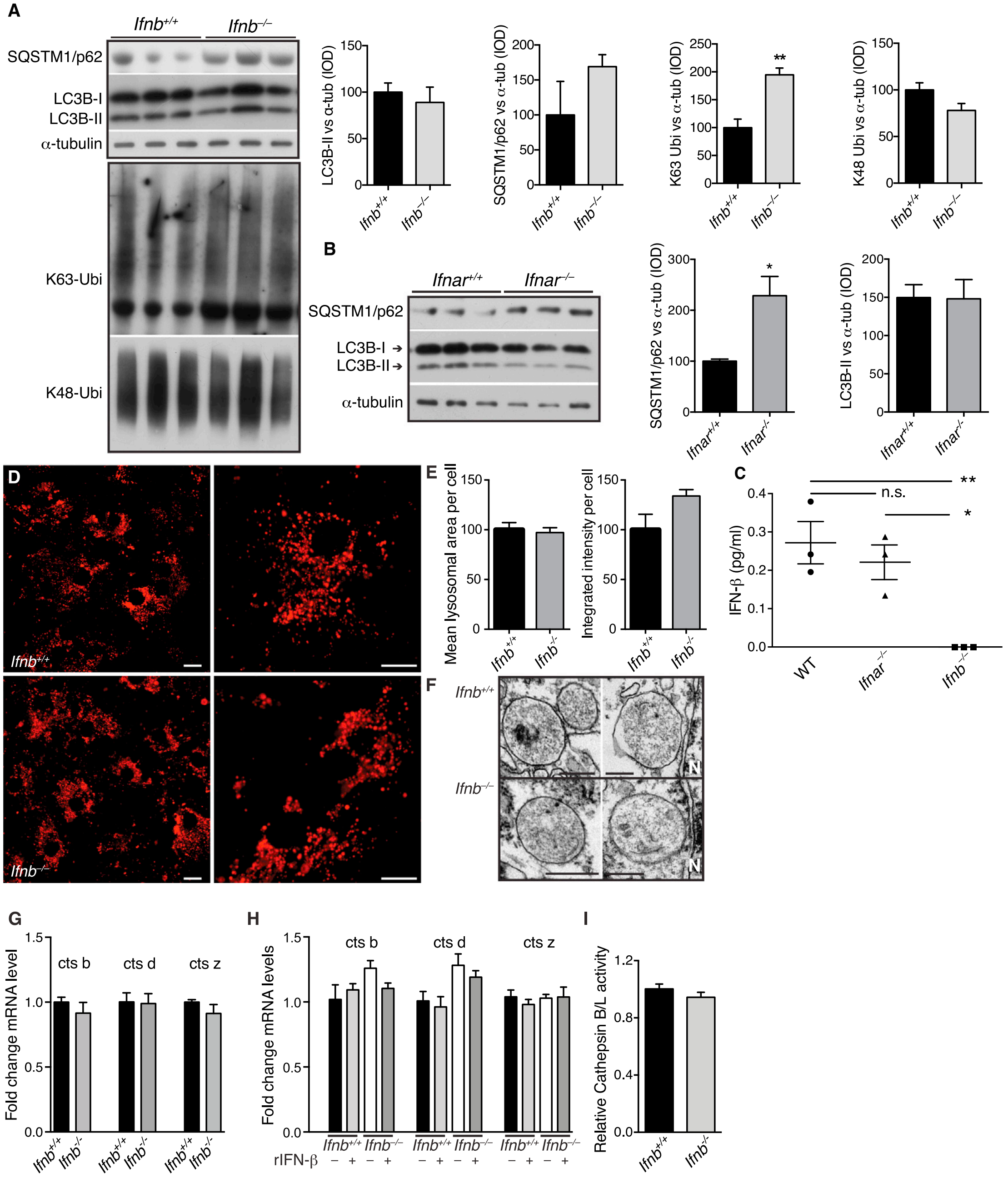

Figure S5. Lysosomes Do Not Change Morphology nor Activity in *Ifnb*$^{-/-}$ Mice, Related to Figure 5

(A) WB of CGN from *Ifnb*$^{+/+}$ and *Ifnb*$^{-/-}$ mice immunoblotted against SQSTM1/p62, LC3B, α-tubulin, K63- and K48-linked ubiquitin. Graphs show mean integrated optical density (IOD) ± SEM, n = 3 of WB bands normalized to α-tubulin.

(B) WB of cultured CGNs from *Ifnar*$^{+/+}$ and *Ifnar*$^{-/-}$ mice. Graphs show mean IOD ± SEM, n = 3.

In (A) and (B) *$p < 0.05$, **$p < 0.01$ by unpaired Student's t test.

(C) ELISA analysis of secreted IFN-β in the culture supernatant from WT, *Ifnar*$^{-/-}$, and *Ifnb*$^{-/-}$ cortical neurons (CN). Graph shows mean pg/ml ± SEM of n = 3 independent CN cultures where each sample was analyzed in triplicates. *$p < 0.05$ and **$p < 0.01$ by one-way ANOVA.

(D) Live cell fluorescence images of 21-day-old cortical neurons from *Ifnb*$^{+/+}$ and *Ifnb*$^{-/-}$ mice loaded with LysoTracker DND-99. Scale bar, 10 μm.

(E) Quantified mean area of LysoTracker DND-99 per cell and integrated intensity per cell. Data represent mean ± SEM of n = 2.

(F) Transmission electron microscopy images showing lysosomes in neurons from thalamus of 9-month-old *Ifnb*$^{+/+}$ and *Ifnb*$^{-/-}$ mice. Scale bar, 200 nm.

(G and H) RT-PCR of cathepsin b, d, and z showing fold changes in ΔCt of *Ifnb*$^{+/+}$ and *Ifnb*$^{-/-}$ obtained from (G) brains or (H) CGN's with or without treatment with rIFN-β (100 U/ml) for 24 hr.

(I) Relative activity of cathepsin B and L in CGN's from *Ifnb*$^{+/+}$ and *Ifnb*$^{-/-}$ mice.

(G–I) Data represent mean ± SEM of n = 3.

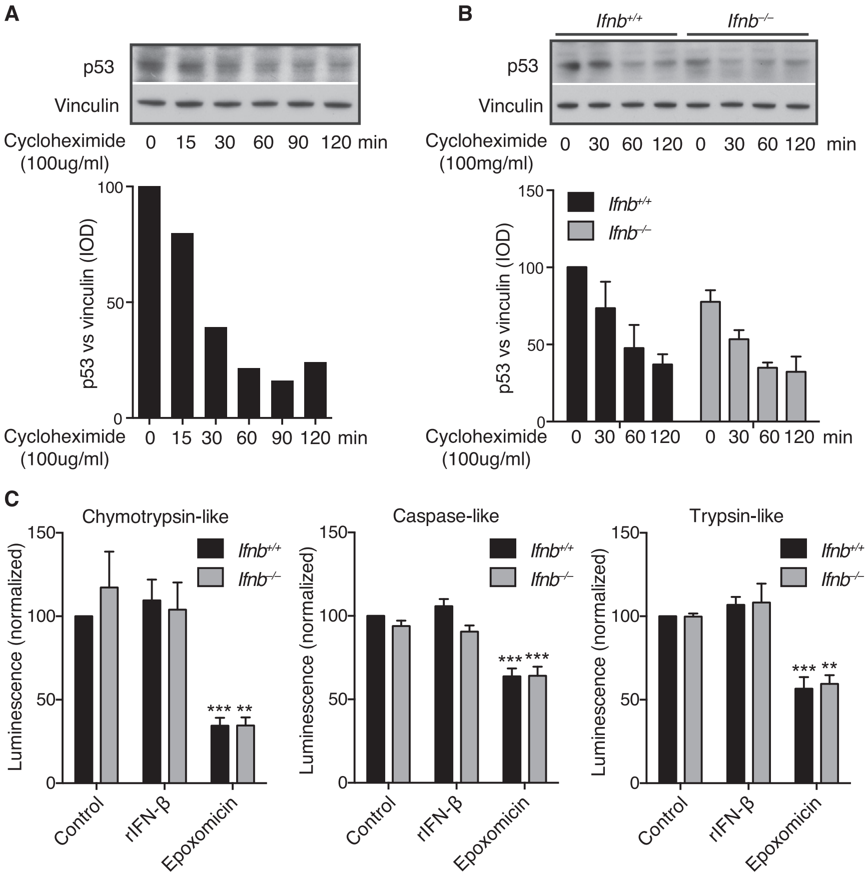

Figure S6. Proteasomal Flux and Activity Are Not Affected by Lack of IFN-β, Related to Figure 6

(A–C) Cortical neurons (CN) cultured for 21 days obtained from *Ifnb*$^{+/+}$ and *Ifnb*$^{-/-}$ mice.

(A-B) WB showing proteasomal kinetic of p53 turn-over in (A) *Ifnb*$^{+/+}$ and (B) *Ifnb*$^{+/+}$ and *Ifnb*$^{-/-}$ CN treated with cycloheximide (100mg/ml) for different time periods as indicated. Graphs show quantified integral optical density (IOD) of p53 WB bands normalized to vinculin and represent (A) one experiment or (B) mean ± SEM of n = 3.

(C) Proteasomal Caspase-like, Trypsin-like, and Chymotrypsin-like activity in CN treated with rIFN-β (100 U/ml), epoxomicin (10 μg/ml), or left untreated, measured by relative luminescence units normalized to control. Data represent mean ± SEM of n = 3; $^{**}p < 0.01$, $^{***}p < 0.001$ by one-way ANOVA.

Cell
Supplemental Information

Lack of Neuronal IFN-β-IFNAR Causes Lewy Body- and Parkinson's Disease-like Dementia

Patrick Ejlerskov, Jeanette Göransdotter Hultberg, JunYang Wang, Robert Carlsson, Malene Ambjørn, Martin Kuss, Yawei Liu, Giovanna Porcu, Kateryna Kolkova, Carsten Friis Rundsten, Karsten Ruscher, Bente Pakkenberg, Tobias Goldmann, Desiree Loreth, Marco Prinz, David C. Rubinsztein, and Shohreh Issazadeh-Navikas

Supplemental Experimental Procedures

Mice and cell culture

Ifnb$^{-/-}$ mice (Erlandsson et al., 1998) were backcrossed 20 generations to B10.RIII or C57BL6, *Ifnar*$^{-/-}$ and *nes*^{Cre}*:Ifnar*$^{fl/fl}$ C57BL6 mice (Prinz et al., 2008). WT were *Ifnb*$^{+/-}$, *Ifnb*$^{+/+}$ littermates or *Ifnar*$^{+/+}$ C57BL6 mice. Mice were housed in standard facilities. Gender- (males and females) and weight-matched mice were used in experiments. Experiments were performed in accordance with the ethical committees in Copenhagen, Denmark, and approved by the respective Institutional Review Boards.

Cerebellar granular neurons (CGN) were obtained from 6- or 7-day-old cerebella and cultured for three or four days on poly-D-lysine coated culture dishes (Liu et al., 2013; Liu et al., 2014). For cortical neuron (CN) cultures the cortex were dissected from 1-day-old mice pups and processed as previously described (Brewer and Torricelli, 2007). Neurons were cultured on poly-D-lysine coated plates (137,500 cells/cm^2) in Neurobasal medium (Gibco) containing B27 (2%) and gentamicin for 21 days. Half of the medium was change every 3rd or 4th day and for long-term treatment, IFN-β (30U/ml) was added at each change.

Generation of Scna$^{-/-}$Ifnb$^{-/-}$ double knock out mice

To generate double knock out mice, C57BL/6JOlaHsd mice (Harlan Laboratories, The Netherlands) with a spontaneous deletion encompassing part of the α-synuclein (*Snca*) gene (Specht and Schoepfer 2004) were purchased. We intercrossed mice lacking α-synuclein (*Scna*$^{-/-}$) with *Ifnb*$^{-/-}$ mice (F_1/heterozygotes). Next we generated double KO mice (*Scna*$^{-/-}$*Ifnb*$^{-/-}$) by intercrossing F_1 mice and genotyping the F_2 mice (Figure S3J).

Antibodies and reagents

Primary antibodies used; polyclonal rabbit NF200 (Sigma), monoclonal mouse LC3B (Cosmo Bio Co LTD, IF), polyclonal rabbit LC3 (Sigma, WB); monoclonal mouse vinculin (Sigma); monoclonal rabbit K-63-linked ubiquitin (Millipore); polyclonal rabbit K-48-linked ubiquitin (Millipore); polyclonal rabbit SQSTM1/p62 (MBL); monoclonal mouse Beclin1 (BD Transduction lab), polyclonal rabbit NBR1 (Proteintech); polyclonal rabbit Atg7 (ProSci); rat monoclonal LAMP1 (BD Biosciences); monoclonal rabbit α-synuclein [EP1646Y] (AbCam, WB and IF); monoclonal rabbit phosphorylated S129 α-synuclein [EP1536Y] (AbCam); polyclonal sheep α-synuclein (Millipore, IF); monoclonal mouse against human α-synuclein (4B12; ThermoScientific); monoclonal mouse against α-synuclein (NCL-L-ASYN; Leica); polyclonal rabbit tyrosine hydroxylase (Millipore); polyclonal rabbit anti-p53 (CM5) (Leica); monoclonal mouse BrdU (RPN202; GE Healthcare), polyclonal goat Doublecortin (C-18; Santa-Cruz); monoclonal mouse α-tubulin (Sigma); polyclonal rabbit phosphorylated tau (T205) (AbCam); polyclonal rabbit GAPDH [EPR1977Y] (AbCam); monoclonal mouse multi-ubiquitin (Medical and Biological Laboratories); mouse monoclonal ubiquitin (Chemicon); monoclonal mouse NeuN, clone A60 (Millipore).

Secondary antibodies used; biotinylated rabbit anti-sheep antibody (Vector Laboratories) and streptavidin-CY3 (1:200, US Biological), goat fluorescein-coupled anti-rabbit antibody (1:200, BD Pharmingen), goat anti-mouse, -rabbit, -rat, -sheep coupled to Alexa Fluor-488, -568, and -633 (1:1000, Invitrogen). Nuclei were stained with DAPI (1:30.000, DAKO) or Hoechst (1µg/ml, Life Technologies).

The specificity of antibodies against α-synuclein for IHC staining was confirmed by blocking the antibodies using saturating concentration of specific recombinant peptides and analyzing immunoreactivity in brain tissues of *Snca*$^{-/-}$*Ifnb*$^{-/-}$ mice.

Reagents used: Oligomycin, Carbonyl cyanide 3-chlorophenylhydrazone (CCCP), Rapamycin, Trehalose (all from Sigma); cycloheximide, epoxomicin, and Ammonium chloride (NH_4Cl) (all from Merck; Calbiochem); and IFN-β (PBL Biomedical Laboratories).

Behavioral measurements

Groups were sex- and weight-matched. Mice were daily habituated to the experimental arena for 3 days. The experimenter was blind to the genotype of the mice. Motor-coordination and -learning were evaluated in an accelerating RotaRod (TSE Systems GmbH). After pre-training at 4 rpm for one minute (min), the speed was gradually increased from 4 to 40 rpm over 3 min, and kept at 40 rpm for additionally 2 min. Time before falling was automatically recorded with a maximum duration of 5 min. Four trials were performed with at least 10 min rest in between. The 3rd trials (peak performances) were considered for statistical comparisons of retention time / motor-coordination in all ages and groups.

Neuromuscular strength was tested by placing the forelimbs of the mouse on a 40 cm elevated stainless steel bar (2mm in diameter), and subsequently measuring the seconds spent hanging for a maximum of 60 seconds (sec). The graph represents an average of two trials with 2 min rest.

Tail-flick was measured by letting mice voluntarily enter a tapered plastic cylinder with air holes. One third of the free-hanging tail was immersed in 50°C or 0°C water and tail-flick latency was recorded with a maximum of 10 sec at 50ºC, or 20 sec at 0ºC. Four trials were conducted with 3-5 min intervals and graph represents mean of the last three trials performed.

The forced swimming test; the mice were individually forced to swim for 5 min inside a 16 cm in diameter glass cylinder tank containing water about 15 cm deep (the subjected individual was not able to stand on the bottom but its tail was able to reach the bottom of the tank) at 25°C, approximately 2°C above RT. The water was changed between sessions to eliminate odour cues. For evaluation of activity or immobility the mice were observed and movements recorded using a stable camera placed in front of the transparent swimming tank. The read-outs were five different behavioral states, i.e. 1) swimming, i.e. active swimming motions, e.g., moving around in the cylinder, 2) diving, i.e. when the whole body of the mouse, including the head is submersed, 3) climbing, i.e. vigorous movements with the forepaws in and out the water, usually directed against the wall of the cylinder, 4)

immobility, i.e. the lack of motion of the whole body, except for small irregular movements necessary to keep its head above water and 5) complete immobility, i.e. the lack of motion of the whole body. The duration of each behavior was recorded, as well as the latency of immobility, a measure that gives information about which group is first to give up on finding an escape. Also, the numbers of faecal boli deposited in the water tank were counted after every session. Rodents that are relatively inactive and have high defecation scores are regarded as fearful, whereas animals with high activity and low defecation scores are considered to be less fearful. The mice were immediately removed from the water tank after the test, dried with paper towels and allowed to dry and warm up for 15 min in a heated container before being returned to their home cages.

Spatial learning and reference memory was assessed in a Morris Water Maze as described (Vorhees and Williams, 2006) with slight modifications. A white plastic water tank (120 cm diameter, ~60 cm high) was filled with water (25°C ±1°C) and made opaque with powdered milk. A circular platform (10 cm in diameter) was submerged 1 cm below the water surface, in a distance of 30 cm from the rim and center of the maze. An automated video tracking system, Ethovision 3.1 (Noldus Information Technology), recorded the time taken to reach the hidden platform during the learning trials and the frequency of platform position crossings during the probe tests. After habituation to the tank, the procedure entailed two learning trial blocks executed on day 1–4 and 6–8 and two probe trials on day 5 and 9. During learning trials (4 per day), mice were placed at one of the 4 starting points that varied semi-randomly between trials and day. Time taken to find the hidden platform (escape latency) and swimming distance were recorded for a maximum of 60 sec. After each trial the mice were left on the platform for 20 sec or placed there manually if they failed to find it within 60 sec. Subsequently, all mice were dried and kept warm. Trial interval was 20 min ±5 min. During probe trials the platform was removed and the number of platform position crossings (14-cm diameter annulus of the platform center) was recorded. The platform was returned immediately after probe 1 and the mice were allowed to stay on it for 20 sec. Statistical analysis was conducted with GraphPad Prism. Two-way repeated-measure ANOVA with Bonferroni *post-hoc* test was conducted to assess the effects of genotype, learning days, and genotype differences within each learning day (trials). Unpaired and paired Student's *t*-test was used to test for significance between *Ifnb*$^{+/+}$ and *Ifnb*$^{-/-}$ mice within the same probe day and between the first and second probe day within each genotype, respectively. Collectively, the prevalence of these deficits was calculated as the behavioral test scores of *Ifnb*$^{-/-}$ mice deviating from the norm; i.e., mean value – (SD/2) of the *Ifnb*$^{+/+}$ group.

Cylinder test was conducted by asymmetrical forelimb use during vertical exploration to provide a validated measure of akinesia in hemiparkinsonian rodents.

Cloning

Mouse *Ifnb* (pCR4IFNb)) was obtained from transOMIC (BC119395). *Ifnb* was transferred to pCSII-GW via pCR8TOPOGW (Invitrogen) with conventional cloning techniques to generate pCSII-

IFNb. pCSII- (without insert) was generated by LR-recombination with an empty pCR8GW vector. Plasmid inserts were verified by sequencing.

Lentiviral production

293FT cells (80% confluent) were transfected with 34.4 μg pCSII- or pCSII-IFNb, 11.8μg pCMV-VSV-G-RSV-Rev (RIKEN BRC), and 23.6μg pMDLg/pRRE (Addgene) with lipofectamine 2000, according to the manufacturers protocol (Invitrogen). After 24 and 48 hours the media was harvested and filtered through a 0.45μm filter (Satorius). The virus supernatant was then precipitated with PEG6000 and titers were determined according to production, concentration and titration of pseudotyped HIV-1-based lentiviral vectors (Kutner et al., 2009).

Surgical procedure with AAV6-hSCNA

In total 30 adult female Sprague Dawley rats (225-250 g at the time of surgery) were used (Taconic). For general anaesthesia we used an isofluorane anesthesia (4% for induction, 1.7-1.9% to maintain anesthesia). Rats were placed in a stereotaxic frame (Stoelting) and virus was injected using a 10 μl Hamilton syringe fitted with a glass capillary with an outer diameter of 250 μm. Three microliters of AAV6-GFP or AAV6-human α-synuclein (hSNCA-WPRE; Vector Biolabs) were injected unilaterally in SN, together with lentiviral vectors pCSII-IFNb (over-expression *Ifnb*) or pCSII control and 10 days later SN were processed for WB. Rats used for cylinder test, IF and IHC staining were injected with hSNCA and control lentivirus in the left brain hemisphere and hSNCA and pCSII-IFNb in the right brain hemisphere of the same rat. Solutions were infused at a rate of 0.2 μl/min and the needle was left in place for an additional 1 min period before it was slowly retracted. Injection was carried out above the SN, at the following coordinates (flat skull position): antero-posterior: -5.3 mm, medio-lateral: -1.7mm, dorso-ventral: -7.2 mm below dural surface as calculated relative to bregma according to the stereotaxic atlas.

Immunohistochemistry, immunofluorescence, and electron microscopy

Fresh brain tissue was snap-frozen and cryosectioned (6-10μm) for immunohistochemistry (IHC) and immunofluorescence (IF) as described (Liu et al., 2006). In some experiments, mice were perfused and brain tissues fixed in 4% paraformaldehyde (PFA), dehydrated in ethanol and embedded in paraffin before sectioning. *In vitro* cell cultures and cryosections were fixed in 4% PFA for 10 and 20 min, respectively. *In vitro* cultures, paraffin- and cryosections were incubated in blocking buffer (0.3% TX100, 5% normal goat serum (NGS), and 2% BSA in phosphate buffered saline (PBS)) for 15-30 minutes, and subsequently in blocking buffer containing primary antibodies overnight at room temperature (RT) in a humidified container. The next day cells were washed three times in PBS, incubated in blocking buffer containing Alexa Fluor® 488, -568, and -633 secondary antibodies where appropriate for 1 hour, and subsequently in PBS containing DAPI for 5 min. Finally, the slides were washed three times in PBS, one time in MQ-water, and mounted with ProLong® Gold antifade

reagent (Life technologies) and cover slip.

For neurogenesis analysis mice were injected with BrdU (75µg/g body weight) i.p. once per day for five consecutive days and sacrificed 2 hours after the last injection. The brain slides were incubation in blocking buffer (0.3% TX100, 5% BSA in PBS$^-$) for 20 min, incubated in 1M HCl, 37°C, for 1 hour, washed 2x5 min in 0.1M sodium borate (pH 8.5), washed 3 times in PBS$^-$ before proceeding with IF staining as above with antibodies against BrdU and doublecortin.

Brain sections for IHC were fixed in either 4% PFA or ice cold acetone for 30 or 5 minutes, respectively, washed in PBS$^-$ and incubated in 3% H_2O_2 for 20 min to quench endogenous peroxidases. Slides were then washed three times in PBS and added blocking buffer (0.3% TX100, 5% NGS, and 2% BSA in PBS) for 30 min, followed by avidin and biotin blocking (Vector labs), and subsequently incubated with primary antibodies over night at 4°C. The following day the slides were washed three times in PBS$^-$ and added IgG specific secondary antibodies coupled to biotin for 1.5 hours at RT, washed three times in PBS$^-$, and subsequently added ABC peroxidase solution (Vector labs) for 1 hour. DAB solution (10 mg DAB, 0.024% H_2O_2, and 10mM NiCl in 15 ml PBS) or NovaRed was added and when sufficient reaction product had developed the slides were washed 20 min in PBS$^-$, dehydrated in ethanol and xylene and cover slips mounted with Pertex. In some experiments the slides were stained with hematoxylene (15 seconds) and washed repeatedly in water prior to the dehydration steps.

Confocal microscopy images were acquired with a Zeiss LSM510 confocal laser scanning microscope with a C-Apochromat ×63, 1.4 NA oil immersion objective, using the diode 405 nm, the argon 488 nm, the helium–neon 543 nm and 633 nm laser lines for excitation of DAPI/Hoechst, Alexa Fluor® 488, 568, and 633, respectively. Confocal sections of 0.8-1.0µm were collected and saved as 512x512-pixel or 1024x1024-pixel images at 12-bit resolution before import to Adope Photoshop CS6, Zeiss Zen, or ImageJ for compilation and quantification. For 3D imaging 15-20µm thick sections were superimposed in the z-plane using the Zeiss Zen software.

IHC images were acquired in a NanoZoomer 2.0-HT digital slide scanner or with an Olympus BX51 microscope using ColorView IIIu software. Neurite length, branches and numbers (from superimposed 3D images), and stereological analysis of tyrosine hydroxylase IHC staining of striatum and substantia nigra were quantified in ImageJ (Fiji version) using Simple Neurite Tracer and Analyze Calibrate O.D., respectively, and for the latter a custom made macro for cell body count per area of substantia nigra. IF staining of brain slices with antibodies against TH were imaged in an IN Cell Analyzer 2200 automated microscope and images quantified using the IN Cell Investigator software and stitched together with CellProfiler software from Broad Institute.

Mitochondrial membrane potential was measured by adding TMRE (20 nM) and Hoechst (Life Technologies) to the medium of primary cortical neurons for 20 min at 37°C. Images were acquired with an IN Cell Analyzer 2200 automated microscope, and number of cells and mean TMRE area were analyzed with CellProfiler software. Neurons were either left untreated or treated with rIFN-

β (100U/ml) for 24 hours, CCCP (16 μM) (added together with TMRE), or oligomycin (5 μg/ml) (added just prior to imaging).

Lysosomes were labeled with 50 nM LysoTracker® Red DND-99 (Life Technologies) for 20 min before live cell imaging in a LSM510 zeiss confocal microscope. HEPES (20 mM) was added to the cultures before imaging. Images were acquired with an IN Cell Analyzer 1000 microscope for quantification of area and integrated fluorescence intensity per cell (90-177 cells were imaged per experiment) using CellProfiler software.

Terminal deoxynucleotidyl transferase dUTP nick end labeling (TUNEL) staining was performed with an *in situ* apoptosis detection kit (R&D Systems) with DAB substrate and methyl green counterstaining, or a TUNEL kit (Calbiochem) with Hoechst counterstaining. Nuclei and $TUNEL^+$ cells were counted from images in a blinded manner.

For transmission electron microscopy (TEM) analysis mice were cardiac perfused with PBS^- followed by a phosphate buffer (PB) containing 2% PFA and 0.2% glutaraldehyde, pH 7.4, and the paratenial and central medial thalamic nuclei were dissected from the brain and processed for epon embedding and ultrathin sectioning on a vibratome. For immuno-EM 12-month-old mice were deeply anesthetized and transcardially perfused with 0.9% saline followed by a fixative containing 4% PFA and 0,05% glutaraldehyde in 0.1 M PB, pH 7.4. Brains were removed and postfixed in the same fixative for 24 h. Tissue blocks were washed with PB and horizontal sections (50 μm) were cut on a vibratome. Sections were treated with 3% H_2O_2 for 10 min. After washing in PB, the sections were incubated in blocking solution containing 5% NGS in 0.1 M PB for 1 h, followed by incubation with primary antibodies (1:20 α-synuclein (NCL-L-ASYN; Leica); for 24 h at 4°C. After washing with PB, the appropriate biotinylated (Vector Laboratories) or gold-labeled secondary antibodies (1.4 nm gold, Nanoprobes) were applied to the sections for 24h. Sections incubated with biotinylated antibodies were washed, incubated for 1 h at RT with ABC solution (Vector Elite Kit; Vector Laboratories) and visualized by using peroxidase reaction with DAB (diaminobenzidine) as the substrate. Sections incubated with Gold-conjugated antibodies were treated with HQ Silver™ Enhancement Kit (Nanoprobes). Finally, the sections were treated with OsO_4, stained with uranyl acetate, dehydrated, and flat-embedded in epoxy resin (Sigma). Ultrathin sections were cut and analyzed with a Philips CM100 electron microscope equipped with a digital camera.

Transfection and plasmid

Autophagy flux was evaluated by lipofectamine™ 2000 (Life Technologies) transfecting cortical neurons (on culture day 19) with the mRFP-GFP-LC3 tandem construct (provided by David Rubinsztein) according to manufacturer's description. HEPES (20 mM) was added to neurons on culture day 21 and the culture dish was placed in a preheated (37°C) microscope stage for live cell confocal imaging.

Stereological analysis

The optical fractionator method was used to estimate the total number of neurons and glia in the hippocampus of the brain sections (80 μm) of each mouse stained with H&E. Point counting techniques, based on the Cavalieri principle, were used to estimate the volume of the hippocampus on one side of the brain.

Western blotting

Cells were lysed in lysis buffer (100 mM NaCl, 50 mM Tris-HCl, 1 mM EGTA, 10 mM $MgCl_2$, pH 7.2) containing 1% Triton X-100, phosphatase and protease inhibitor cocktail (Sigma) for 5 min at RT and hereafter kept on ice. Cell lysates were centrifuged at 16,100g for 5 min at 4ºC, and protein concentrations of supernatants were determined with BCA assay (Pierce). Lysates were added 4xLDS containing 100 mM DTT and equal amounts of protein were loaded and separated on 4-12% or 16% SDS-PAGE gels, and transferred to Hybond C extra or PVDF membranes. Membranes were incubated with primary antibodies over night, washed three times in washing buffer (0.1% Tween-20 in PBS), incubated with horse radish peroxidase (HRP) coupled secondary antibodies for 1 hour, washed three times in washing buffer, and developed using chemiluminescent HRP detection substrate (Millipore). When extracting proteins from brain tissue, one half brain (cut sagittally) or the basal ganglia were added lysis buffer (same as above) and homogenized in a cell douncer. The homogenate was then centrifuged two consecutive times at 16.100g for 10 min at 4°C, and the final supernatant was collected and processed as above. The TX-100 insoluble pellets from the two centrifugations steps were lysed in UREA buffer (8M Urea, 5% SDS and 1 mM EGTA) and subsequently sonicated. The cell lysates were then processed as above.

Enzyme-Linked ImmunoSorbent Assay (ELISA)

Supernatant of CN cultures from WT, *Ifnb*$^{-/-}$ and *Ifnar*$^{-/-}$ were collected after 3 days of culture for ELISA. Mouse IFN-β ELISA was performed according to manufacturer (VeriKine-HS^{TM}) mouse IFN-β Serum ELISA kit (Catalog No.42410, PBL assay science). Medium from rIFN-β treated CNs served as a positive control.

Quantitative real time (RT)-PCR

Total RNA was isolated using a total RNA purification kit (RNeasy Plus Mini Kit, 74134, QIAGEN) and reverse transcribed into cDNA (iScript cDNA Synthesis kit, 170-8891, Bio-Rad). qPCR reactions were performed by Maxima™ SYBR Green/ROX qPCR Master Mix (K0002, Fermentas)

Relative mRNA expression was calculated using *Gapdh* and *Actb* (beta-actin) gene as endogenous reference for mouse and rat brain tissue, respectively. The primers were purchased from SABioscience, QIAGEN, as following: Mouse primers: *Snca,* PPM25867E; *Gapdh,* PPM02946E; p62: for (5’-CCT ATA CCC ACA TCT CCC AC-3’); rev (5’-TAC AAG AAT GCC AAG ACA

CTG-3’); CatB: for (5’-CTG CTT ACC ATA CAC CAT CC-3’); rev (5’-ATC TCC TTC ACA CTG TTA GAC-3’); CatD: for (5’-CTG TAT CGG TTC CAT GTA AGT-3’); rev (5’-CCA AGC ATT AGT TCT CCT CC-3’); CatZ: for (5’-CCG TTA TCA ACC ACA TCA TCT C-3’); rev (5’-ATC CTC ATC CAG CCT TTC TC-3’).

The following primers were used for genotyping *Snca*$^{-/-}$ mice containing the Del(6)Snca1Slab deletion and *Ifnb*$^{-/-}$ mice containing the neo-insert: *Snca* exon VI 5': AAG ACT ATG AGC CTG AAG CCT AAG; *Snca* exon VI 3': AGT GTG AAG CCA CAA CAA TAT CC; D6Slab17-pA: TTG ATA GTT CCA CTG TTC TGG C; D6Slab17-pB: GTA ACA ATA CAG CAA GAG ATA C; *Ifnb* sense: TAT CTT CAG GGC TGT CTC CTT TCT; *Ifnb* anti-sense: ACC TGT TGT TCA TGA TGG AAG CCA; λ2 anti-sense: GGC ATA GTT ACT AGT TGT AAC AGC.

Determination of Dopamine and its Metabolites by HPLC

The striatal tissues of the rats were dissected using stereotaxic coordination, homogenized in perchloric acid 0.1 N, centrifuged at 14,000 Å~ g for 30 min and 200 µl of the supernatant was filtered through a glass 0.22 µm filter (Avantec; 13CP020AS). Filtered supernatant (20 µl) was examined for dopamine (DA), 3,4-dihydroxyphenylacetic acid (DOPAC), and homovanillic acid (HVA) levels by reversed-phase HPLCy (RPHPLC) with electrochemical detection. Typically, 20 µl sample was injected onto a Prodigy C18 column (100 Å~ 2 mm ID, 3-µm particle size; Phenomenex). The mobile phase consisted of 93% (wt/vol) of 94.2 mM NaH2- PO4, 0.98 mM octanesulfonic acid, 0.06 mM Na2EDTA, adjusted to pH 3.2 with 1M phosphoric acid and 9% acetonitrile (vol/vol). The flow-rate was 0.15 ml/min.

Cathepsin activity

Cathepsin activity was obtained from whole brain lysates. Mice were sacrificed at day 7 and dissected brains were snap-frozen in liquid nitrogen. The tissue was homogenized by 15 firm strokes using a cell douncer in acidic Mantle-Buffer (50 mM NaAcetate, 100 mM NaCl, 1 mM DTT, pH 5.5) (1:10 weight to volume ratio) on ice. Lysates were incubated at 4°C for 15 minutes while rotating. After centrifugation at 10,000 rpm for 15 minutes at 4°C, the supernatants were taken and protein concentration determined. For measurements of cathepsin activity, 50 µl brain lysate was mixed with 50 µl of cathepsin reaction buffer (50 mM NaAcetate, 4 mM EDTA, 8 mM DTT, and 0.5 mM Pefablock) in a black 96-well plate. The cathepsin reaction buffer contained a cathepsin B/L specific substrate Z-phe-arg-AFC (MP Biomedicals). The plate was incubated at 37°C in a FluoStar Optima plate reader and kinetic fluorescence (excitation filter at 400nm and emission filter at 480nm, recording once a minute) was measured for 30-35 minutes. Enzymatic activity was determined by averaging 4-5 time points at the steepest point of the fluorescence slope. For each animal the assay was run in triplicates. As controls, cathepsin inhibitors were used at indicated concentrations, namely: zFA-fmk, ALLN and CA-074-ME.

Proteasomal activity and flux

Proteasomal activity, hereunder caspase-like, trypsin-like, and chymotrypsin-like activity, was measured in cortical neurons with Proteasome-Glo^{tm} (Promega cat. no. 1180) according to manufactures description. CN were left untreated or pre-treated with rIFN-β (100 U/ml) or epoxomicin (10 nM) for 24 hours before adding Proteasome-Glo^{tm}. The plate was read in Glomax multi detection system (Promega) set for luminescence detection. Flux and degradation of the proteasomal substrate p53, was assessed by treating CN with cycloheximide (100 μg/ml) for 15 to 120 minutes, and the p53 levels were subsequently measured by WB.

Expression Affymetrix microarrays

RNA was extracted with TRI reagent (Sigma) and DNAse I (Invitrogen) from 3-day-old *Ifnb*$^{+/+}$ and *Ifnb*$^{-/-}$ CGN cultures in triplicates with or without rIFN-β (100 U/ml) treatment for the last 24 hours. We assessed RNA quality and concentration with an Agilent 2100 Bioanalyzer and Nanodrop ND-1000. Data from Affymetrix 430 2.0 microarray chips (SCIBLU, Affymetrix) was analyzed with Arraystar 3 software (DNA STAR Inc.), which were quantile-normalized and processed by the RMA (Affymetrix) algorithm. We log_2-transformed the intensity values and normal-distributed data were tested in unpaired, two-tailed Student's *t*-tests, assuming equal variance and filtering for differential regulation confidence of 95% (P<0.05). Venn diagrams were created with oneChannelGUI (Bioconductor) from normalized RMA-analyzed data. Hybridization quality graphs are available on request. Quantile-normalized RMA-treated data selected using a 1.4-fold cut-off were analyzed with Ingenuity Pathway Analysis software. All raw microarray data are deposited at the Gene Expression Omnibus (GEO) database under the accession number GSE63815. Venn diagrams from normalized data were created with oneChannelGUI (www.bioconductor.org).

Heatmaps of the GSEA data were generated by extracting lists of core enriched genes from Autophagy (hereunder Lysosome and mTOR), Parkinson's, Huntington's, Alzheimer's, or Prion disease pathways of the GSEA analysis in R (Data File S1). Fold change gene expression values were calculated for the lists and the resulting tables were fed into Heatmap2 in R to construct the heatmaps.

Comparison between gene profile of *Ifnb*$^{-/-}$ CGNs and published models for PD, and HD disease affymetrix data (GSE4758, GSE9038) were quantile normalized together and summarized in R using the RMA algorithms from the affy-package. Identification of differential expression was performed individually within each experiment for our data as well as the published PD and HD models by comparing control/wild-type samples with transgenic samples using a standard ANOVA. In total, three sets of differentially expressed genes were generated and depicted in heatmaps: 1) Genes differentially expressed in our data (p<0.001); 2) Genes differentially expressed in both our data and the HD set (p<0.01); 3) Genes differentially expressed in both our and the PD set (p<0.01). The corresponding heatmaps were created using the pheatmap function in R. The settings were always the

same; Hierarchical clustering with Wards method on Euclidean distances where clustering was performed.

Statistical analysis

Data were analyzed with unpaired and paired two-tailed Student's *t*-tests, ANOVA, Mann-Whitney U test and Kruskal-Wallis test, with $^{*}P<0.05$; $^{**}P<0.01$; $^{***}P<0.001$ as significant values. Error bars are SEM.

Table S1 | Lack of *Ifnb* Gene is Associated with Increasing Penetrance in Behavioral Impairments. Related to Figure 1.

Age (months)	No. of mice	MWM*	No. of mice	RotaRod	Hanging test	Heat-induced pain	Cold-induced pain
1.5	8–15	11%	9–10	0%	0%	0%	0%
3	8–17	44%	8–11	62.5%	70%	87.5%	80%
6	8–16	49%	9–13	70%	92.3%	90%	78%
12	10–21	69%	10–13	69.2%	ND	ND	ND

Penetrance; i.e. the percentage of affected mice were calculated as the behavioral test scores of *Ifnb*$^{-/-}$ mice deviating from the norm; i.e., mean value – (SD/2) of the *Ifnb*$^{+/+}$ group. Two-way ANOVA were used to assess the age-dependent cognitive impairment in *Ifnb*$^{-/-}$ mice (*) (11%, 44%, 49%, 60%, indicate percentage of mice that did not improve their performance between probe 1 and 2 in each age group). MWM = Morris Water Maze, ND = not done.

Table S2 | Canonical KEGG Pathways from GSEA Analysis of *Ifnb*$^{-/-}$ Neurons (+/–) Recombinant IFN-β. Related to Figure 4.

	Gene set	Size	ES	NES	NOM p-val	FDR q-val	FWER p-val	Rank at max
1	KEGG FC GAMMA R MEDIATED PHAGOCYTOSIS	85	0,41	1,52	0,0000	0,2660	0,3153	2162
2	KEGG NEUROTROPHIN SIGNALING PATHWAY	115	0,30	1,49	0,0000	0,1847	0,3875	1397
**3**	**KEGG MTOR SIGNALING PATHWAY**	**44**	**0,34**	**1,38**	**0,0000**	**0,3694**	**0,6319**	**2121**
4	KEGG ENDOCYTOSIS	146	0,27	1,28	0,0000	0,6215	0,6611	4958
5	KEGG RENAL CELL CARCINOMA	67	0,25	1,22	0,1465	1,0000	1,0000	5308
6	KEGG B CELL RECEPTOR SIGNALING PATHWAY	70	0,32	1,22	0,1910	0,6208	1,0000	5723
7	KEGG TYPE II DIABETES MELLITUS	43	0,32	1,21	0,0708	0,5576	1,0000	1951
**8**	**KEGG LYSOSOME**	**109**	**0,24**	**1,20**	**0,0785**	**0,5243**	**1,0000**	**4992**
9	KEGG TYPE I DIABETES MELLITUS	20	0,42	1,17	0,1340	0,5688	1,0000	1075
10	KEGG AXON GUIDANCE	125	0,23	1,16	0,0970	0,5403	1,0000	5879
11	KEGG ALDOSTERONE REGULATED SODIUM REABSORPTION	36	0,39	1,11	0,1681	0,6792	1,0000	2074
12	KEGG ADIPOCYTOKINE SIGNALING PATHWAY	64	0,23	1,09	0,1444	0,6854	1,0000	1774
13	KEGG GLYCOSYLPHOSPHATIDYLINOSITOL GPI ANCHOR	22	0,28	1,07	0,1597	1,0000	1,0000	1695
14	KEGG ALANINE ASPARTATE AND GLUTAMATE METABOLISM	30	0,30	1,06	0,2208	0,6861	1,0000	1330
15	KEGG LEUKOCYTE TRANSENDOTHELIAL MIGRATION	102	0,28	1,05	0,2028	0,6681	1,0000	2057
16	KEGG FC EPSILON RI SIGNALING PATHWAY	76	0,28	1,02	0,2854	0,6840	1,0000	1767
17	KEGG LEISHMANIA INFECTION	49	0,30	1,01	0,1431	0,6653	1,0000	2057
18	KEGG NUCLEOTIDE EXCISION REPAIR	43	0,21	1,01	0,3563	0,6319	1,0000	2579
**19**	**KEGG REGULATION OF AUTOPHAGY**	**24**	**0,32**	**1,00**	**0,2618**	**0,6215**	**1,0000**	**3614**
20	KEGG RNA DEGRADATION	50	0,18	1,00	0,1958	0,6014	1,0000	2789

GSEA analysis was made on Affymetrix data that was quantile normalized and summarized for each comparison using RMA algorithm. ES = enrichment score; NES = normalized enrichment score; NOM p-val = nominal p-value; FDR q-val = False Discovery Rate; FWER p-val = family-wise error rate; RANK AT MAX = position in the ranked list at which the maximum enrichment score occurred.

Supplemental References

Brewer, G.J., and Torricelli, J.R. (2007). Isolation and culture of adult neurons and neurospheres. Nat Protoc *2*, 1490-1498.

Erlandsson, L., Blumenthal, R., Eloranta, M.L., Engel, H., Alm, G., Weiss, S., and Leanderson, T. (1998). Interferon-beta is required for interferon-alpha production in mouse fibroblasts. Curr Biol *8*, 223-226.

Kutner, R.H., Zhang, X.Y., and Reiser, J. (2009). Production, concentration and titration of pseudotyped HIV-1-based lentiviral vectors. Nat Protoc *4*, 495-505.

Liu, Y., Carlsson, R., Ambjorn, M., Hasan, M., Badn, W., Darabi, A., Siesjo, P., and Issazadeh-Navikas, S. (2013). PD-L1 expression by neurons nearby tumors indicates better prognosis in glioblastoma patients. J Neurosci *33*, 14231-14245.

Liu, Y., Carlsson, R., Comabella, M., Wang, J., Kosicki, M., Carrion, B., Hasan, M., Wu, X., Montalban, X., Dziegiel, M.H., *et al.* (2014). FoxA1 directs the lineage and immunosuppressive properties of a novel regulatory T cell population in EAE and MS. Nat Med *20*, 272-282.

Liu, Y., Teige, I., Birnir, B., and Issazadeh-Navikas, S. (2006). Neuron-mediated generation of regulatory T cells from encephalitogenic T cells suppresses EAE. Nat Med *12*, 518-525.

Prinz, M., Schmidt, H., Mildner, A., Knobeloch, K.P., Hanisch, U.K., Raasch, J., Merkler, D., Detje, C., Gutcher, I., Mages, J., *et al.* (2008). Distinct and nonredundant in vivo functions of IFNAR on myeloid cells limit autoimmunity in the central nervous system. Immunity *28*, 675-686.

Vorhees, C.V., and Williams, M.T. (2006). Morris water maze: procedures for assessing spatial and related forms of learning and memory. Nat Protoc *1*, 848-858.
